# Unknown Areas of Activity of Human Ribonuclease Dicer: A Putative Deoxyribonuclease Activity

**DOI:** 10.3390/molecules25061414

**Published:** 2020-03-20

**Authors:** Marta Wojnicka, Agnieszka Szczepanska, Anna Kurzynska-Kokorniak

**Affiliations:** Department of Ribonucleoprotein Biochemistry, Institute of Bioorganic Chemistry Polish Academy of Sciences, 61-704 Poznan, Poland; mwojnicka@ibch.poznan.pl (M.W.); aszczepanska@ibch.poznan.pl (A.S.)

**Keywords:** ribonuclease Dicer, PAZ domain, RNase activity, DNase activity, substrate binding pockets, non-canonical Dicer substrates

## Abstract

The Dicer ribonuclease plays a crucial role in the biogenesis of small regulatory RNAs (srRNAs) by processing long double-stranded RNAs and single-stranded hairpin RNA precursors into small interfering RNAs (siRNAs) and microRNAs (miRNAs), respectively. Dicer-generated srRNAs can control gene expression by targeting complementary transcripts and repressing their translation or inducing their cleavage. Human Dicer (hDicer) is a multidomain enzyme comprising a putative helicase domain, a DUF283 domain, platform, a PAZ domain, a connector helix, two RNase III domains (RNase IIIa and RNase IIIb) and a dsRNA-binding domain. Specific, ~20-base pair siRNA or miRNA duplexes with 2 nucleotide (nt) 3’-overhangs are generated by Dicer when an RNA substrate is anchored within the platform-PAZ-connector helix (PPC) region. However, increasing number of reports indicate that in the absence of the PAZ domain, binding of RNA substrates can occur by other Dicer domains. Interestingly, truncated variants of Dicer, lacking the PPC region, have been found to display a DNase activity. Inspired by these findings, we investigated how the lack of the PAZ domain, or the entire PPC region, would influence the cleavage activity of hDicer. Using immunopurified 3xFlag-hDicer produced in human cells and its two variants: one lacking the PAZ domain, and the other lacking the entire PPC region, we show that the PAZ domain deletion variants of hDicer are not able to process a pre-miRNA substrate, a dsRNA with 2-nt 3ʹ-overhangs, and a blunt-ended dsRNA. However, the PAZ deletion variants exhibit both RNase and DNase activity on short single-stranded RNA and DNAs, respectively. Collectively, our results indicate that when the PAZ domain is absent, other hDicer domains may contribute to substrate binding and in this case, non-canonical products can be generated.

## 1. Introduction

Ribonuclease Dicer is one of the key enzymes involved in the biogenesis of small regulatory RNAs (srRNAs), such as microRNAs (miRNAs) and small interfering RNAs (siRNAs) [1]. It cleaves 50–70-nucleotide (nt) single-stranded pre-miRNAs with hairpin structures or double-stranded RNAs (dsRNAs) into functional ~22-nt miRNAs or siRNAs, respectively. The Dicer-produced miRNA and siRNA duplexes are loaded into a multi-protein complex referred to as the RNA-induced silencing complex (RISC) [2]. During RISC activation, one strand of the RNA duplex is discarded (designated as “passenger”) while the mature miRNA or siRNA strand (designated as “guide”) remains in the RISC. The activated RISC targets the cellular mRNA complementary to the guide strand [2,3,4]. Depending on the degree of complementarity between the small RNA and the target molecule, RISC binding results in either mRNA cleavage and degradation or translational repression [5]. srRNAs play significant roles in many biological processes, including developmental timing, growth control, differentiation [6], apoptosis, chromatin remodeling [7] and genome rearrangements [8] and viral defense [9,10]. In humans, the vast majority of srRNAs are miRNAs. They have been found to control the expression of most human protein-coding genes through the miRNA pathway [11,12]. Thus, the cellular levels of miRNAs and other components of miRNA pathways must be tightly controlled. Aberrant regulation of miRNA levels can initiate pathological processes, including carcinogenesis [13,14], neurodegenerative [15], immune system and rheumatic disorders [16].

Dicer-type proteins belong to the ribonuclease III (RNase III) family enzymes [17]. Human Dicer (hDicer) is a 220-kDa multi-domain protein comprising an amino (N)-terminal putative helicase domain, a DUF283 domain (domain of unknown function), platform, Piwi–Argonaute–Zwille (PAZ) domain, a connector helix, two RNase III domains (RNase IIIa and RNase IIIb) and a dsRNA-binding domain (dsRBD) [18,19,20,21]. The RNase IIIa and RNase IIIb domains form a single dsRNA-cleavage center. Each RNase III domain cleaves one strand of the RNA duplex, generating products with 2-nt 3ʹ-overhangs on both ends [21]. The structural and biochemical analyses of hDicer indicate that an RNA substrate is recognized and anchored within the platform-PAZ-connector helix region, referred as the “PPC” region [22,23,24,25,26]. The PPC contains two adjacent pockets, a 2-nt 3′-overhang-binding pocket (designated as “3′-pocket”) and a phosphate-binding pocket (designated as “5′-pocket”) [23,27]. Binding of a substrate within the 5′-pocket has been proposed to be efficient only when the substrate ends are less stably base-paired, which is characteristic for miRNA precursors [27]. Moreover, available structures and models of Dicer-type proteins indicate that the distance from the PAZ domain to the RNase III domains determines the length of the RNA produced by Dicer [24,25,26]. The structural element that determines this distance is probably the α helix located in the connector helix domain [26]. Therefore, the connector helix positioned between the PAZ and RNase III domains is considered as a molecular ruler measuring the distance of approximately ~22 nt from the substrate ends [19,26]. In addition, spatial orientation of the connector helix, platform and PAZ relative to the RNase III domains seems to be crucial for measuring dsRNA of the defined length [28].

Interestingly, it has been demonstrated that under specific circumstances hDicer can produce RNA species shorter or longer than characteristic ~22-nt RNAs [29,30]. For example, during inflammation hDicer has been shown to generate ~55-nt and ~10- to 12-nt RNAs from a pre-miRNA substrate due to the interactions with the inflammation-induced protein, 5-lipooxygenase [29]. Moreover, engineered hDicer variants comprising only RNase III domains, and the dsRBD have been demonstrated to produce ~15-nt RNAs [30]. These data suggest that when the PAZ domain is not available or absent, other Dicer domains can be involved in substrate anchoring, and as a consequence products distinct from ~22-nt miRNA or siRNA can be generated. Interestingly, in 2010 Nakagawa et al. reported that in *Caenorhabditis elegans* during apoptosis a CED-3 caspase cleaved Dicer within the RNase IIIa domain yielding a short carboxyl (C)-terminal fragment with one intact RNase III domain (RNase IIIb) [31]. The authors showed that the cleavage of Dicer by a CED-3 caspase abolished its RNase activity, but they found that the C-terminal fragment of Dicer exhibited a DNase activity. Such a truncated form of *C. elegans* Dicer lacked the PPC region [31,32]. These findings have inspired us to investigate how the lack of the PAZ domain, or the entire PPC region, would influence the cleavage activity of hDicer.

## 2. Results

### 2.1. Production of the hDicer PAZ Domain Deletion Variants

**Figure 1:**
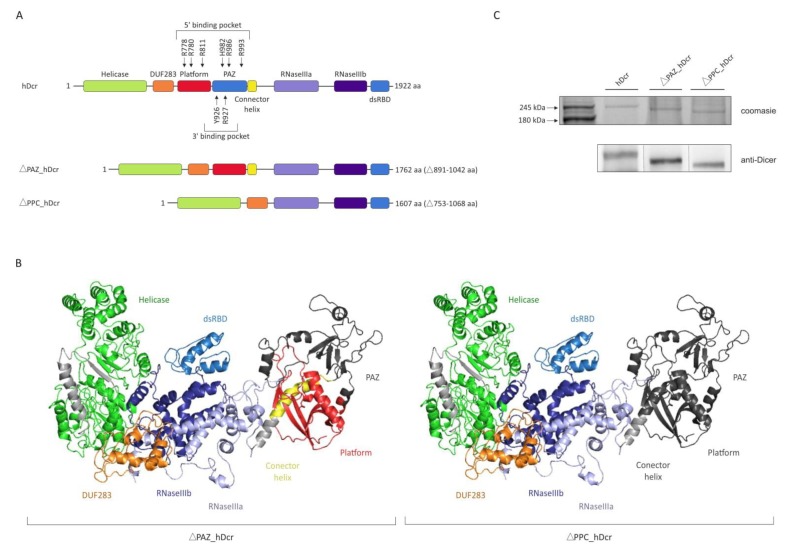
**Full-length hDicer and the PAZ domain deletion variants**. (**A**) Schematic representation of hDicer domain architecture and the PAZ domain deletion variants: ΔPAZ_hDcr and ΔPPC_hDcr. (**B**) The 3D structure of hDicer (PDB entry 5ZAL) visualized by PyMOL. The removed domains in ΔPAZ_hDcr (**left panel**) and ΔPPC_hDcr (**right panel**) are indicated in dark grey. (**C**) PAGE analysis of hDcr, ΔPAZ_hDcr and ΔPPC_hDcr preparations. The C-terminally 3xFlag-tagged proteins were expressed in 293T NoDice cells, then they were purified by immunoprecipitation and analyzed by SDS-PAGE followed by: Coomassie Blue Staining (**upper panel**) or Western blotting with anti-Dicer antibodies specific to the 1701-1912 aa hDicer region (**bottom panel**).

To assess the contribution of the PAZ domain to the cleavage activity of hDicer, we obtained a recombinant hDicer protein (called “hDcr”) and its two variants, one with a deletion of the PAZ domain, including the 3′-pocket, spanning amino acids 891-1042 (called “ΔPAZ_hDcr”), and the other, with a deletion of the entire PPC region, including both substrate binding pockets, spanning amino acids 753-1068 (called “ΔPPC_hDcr”). The PAZ domain and the PPC region, which were deleted in ΔPAZ_hDcr and ΔPPC_hDcr variants respectively, display the structural integrity (Figure 1A,B). The coding sequences of the full-length wild type hDicer and variants lacking either the PAZ domain or the PPC region, were obtained in PCR reactions using full-length cDNA encoding transcript variant 2 of *DICER1* (NM_030621) as a template (for details please see Materials and Methods). Then, the cDNA sequences were cloned into the SureVector expression vector to form fusion proteins with the 3xFlag peptide. 3xFlag-tag-bearing expression plasmids were used for the transfection of 293T NoDice cells (Dicer-deficient cells) [33]. 72 h after transfection, hDcr, ΔPAZ_hDcr and ΔPPC_hDcr proteins were isolated and purified from cell extracts by immunoprecipitation, with anti-Flag antibody conjugated to agarose beads (Figure 1).

### 2.2. RNase Activity of Full-Length hDicer and the PAZ Domain Deletion Variants

**Figure 2:**
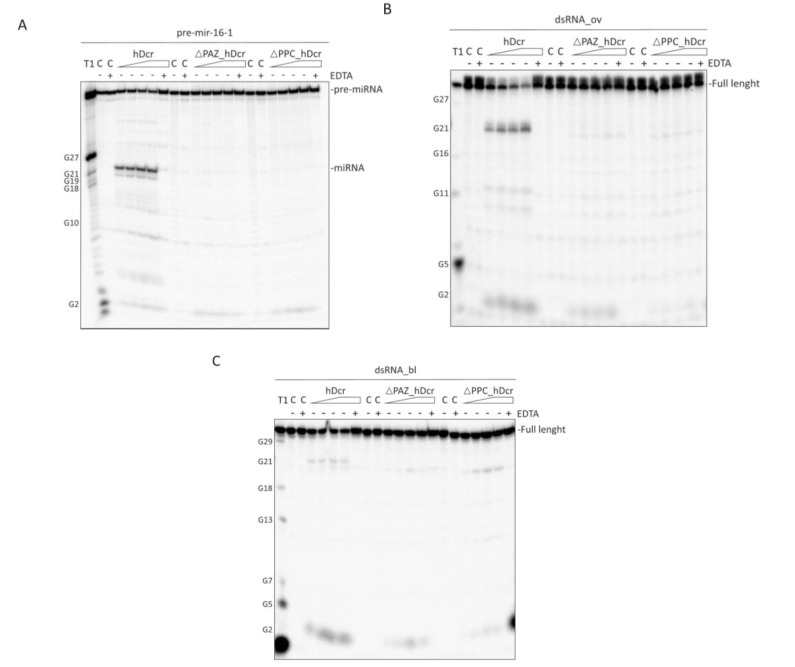
**Comparison of the RNase activity of full-length hDicer (hDcr) and the PAZ domain deletion variants ΔPAZ_hDcr and ΔPPC_hDcr on Dicer canonical substrates.** (**A**–**C**) The results of the RNA-cleavage assays involving: pre-mir-16-1 (**A**), 30-bp dsRNA with 2-nt 3ʹ-overhangs (**B**), 30-bp blunt-ended dsRNA (**C**), and increasing amounts of the respective protein (hDcr, ΔPAZ_hDcr or ΔPPC_hDcr): 12.5, 25, 50, 75 nM (represented by a triangle). (*C-*) controls incubated without a protein. (*C+EDTA*) controls incubated without a protein but with 25 mM EDTA. (*+EDTA*) supplementation of the reaction buffer with 25 mM EDTA. EDTA by chelating the Mg2+ cations present in the reaction buffer inhibits the RNase III-specific cleavage. (T1) G-ladder generated with RNase T1.

Obtained protein preparations: hDcr, ΔPAZ_hDcr and ΔPPC_hDcr were assayed for the RNase activity using a pre-miRNA substrate (pre-mir-16-1) and two 30-base pair (bp) RNA duplexes: one having 2-nt 3ʹ-overhanging ends (called “dsRNA_ov”), and the other, blunt-ended (called “dsRNA_bl”). The cleavage assays involved increasing amount of the protein (12.5, 25, 50, 75 nM) and ~5 nM either 5′-^32^P-labeled pre-mir-16-1 or a dsRNA substrate (dsRNA_ov or dsRNA_bl), in which one of the two strands was 5′-^32^P-labeled. Two control reactions without the protein were also prepared: one containing only the substrate in the reaction buffer (*C-*), and the other containing the substrate in the reaction buffer with addition of 25 mM EDTA (*C+EDTA*). Another control reaction included the substrate, 75 nM protein and 25 mM EDTA in the reaction buffer *(+EDTA*). All reactions were carried out for 30 min at 37 °C, and then they were halted by adding 1 volume of 10 M urea loading buffer, and heating for 5 min at 95 °C. Reaction mixtures were separated on a 15% polyacrylamide gel with 7 M urea and 1×TBE, (PAGE) and visualized by phosphorimaging (Figure 2).

In the assay with pre-mir-16-1, hDcr efficiently produced 22-nt RNAs and, less efficiently, 21-nt RNAs. As expected, we did not observe any miRNA-size products in the reactions carried out with ΔPAZ_hDcr or ΔPPC_hDcr (Figure 2A). We neither observed any cleavage products in all control reactions. In the case of the assays involving the dsRNA substrate with 2-nt 3ʹ-overhanging ends (dsRNA_ov), hDcr produced very efficiently 21-nt RNAs, and no such products were observed in the reactions conducted with ΔPAZ_hDcr or ΔPPC_hDcr (Figure 2B). 21-nt RNA products were also observed in the assays carried out with hDcr and the blunt-ended substrate (dsRNA_bl) (Figure 2C). However, in this case hDcr generated 21-nt RNAs less efficiently, compared with the efficiency of 21-nt RNA production in the assays involving dsRNA_ov. Additionally, in reactions with both 2-nt 3ʹ-overhang (Figure 2B) and blunt ended dsRNA (Figure 2C), we observed very short cleavage products (~2-3 nt) that accumulated with an increase of the hDcr amount. These products, although much less abundant, were also observed in reactions with ΔPAZ_hDcr and ΔPPC_hDcr variants, and in the reactions involving a pre-mir-16-1 substrate (please, compare Figure 2A–C). ~2-3-nt RNA products were not produced in all control reactions. It is important to mention that short RNA products in the range of ~2-5 nt, which resulted from Dicer cleavage of both short hairpin RNAs and dsRNAs, were also observed by other groups [34].

**Figure 3:**
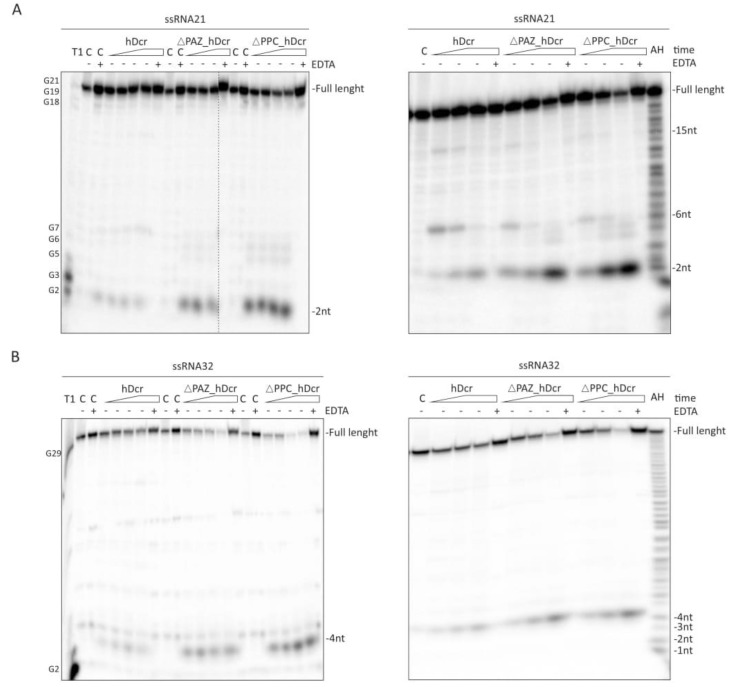
**Comparison of the RNase activity of full-length hDicer (hDcr) and the PAZ domain deletion variants ΔPAZ_hDcr and ΔPPC_hDcr on short ssRNA substrates.** (**A**,**B**; **left panels**) The results of the RNA-cleavage assays involving: 21-nt ssRNA (**A**; **left panel**) and 32-nt ssRNA (**B**; **left panel**), and increasing amounts of the respective protein (hDcr, ΔPAZ_hDcr or ΔPPC_hDcr): 12.5, 25, 50, 75 nM (represented by a triangle). (**A**,**B**; **right panels**) The results of the time-dependent cleavage of 21-nt ssRNA (**A**; **right panel**) and 32-nt ssRNA (**B**; **right panel**). Triangles represent time points: 15 min, 30 min, 1h. Reactions were carried out with 50 nM protein (hDcr, ΔPAZ_hDcr or ΔPPC_hDcr). (*C-*) controls incubated without a protein. (*C+EDTA*) controls incubated without a protein but with 25 mM EDTA. (*+EDTA*) supplementation of the reaction buffer with 25 mM EDTA. EDTA by chelating the Mg2+ cations present in the reaction buffer, inhibits the RNase III-specific cleavage. (T1) G-ladder generated with RNase T1. (AH) Ladder generated by alkaline hydrolysis of 5′-^32^P-labeled ssRNAs.

Previously we have shown that hDicer, apart from the canonical substrates, binds structurally diverse short ssRNA molecules [35,36,37,38]. Consequently, we decided to test whether hDcr and its variants lacking the PAZ domain would cleave short ssRNAs. For these studies, we applied 21-nt RNA (called “RNA21”) and 32-nt RNA (called “RNA32”). Cleavage assays involved ~5 nM 5′-^32^P-labeled ssRNA and increasing amount of the protein (12.5, 25, 50, 75 nM). Three control reactions were prepared, as mentioned above. Under the applied reaction conditions, all proteins: hDcr, ΔPAZ_hDcr and ΔPPC_hDcr, cut out ~2-nt RNA products from the 5′-end of RNA21 (Figure 3A; left panel), and ~4-nt RNA products from the 5′-end of RNA32 (Figure 3B; left panel). These products were not observed in all control reactions. We also noticed that ~2-nt and ~4-nt RNAs were produced more abundantly in reactions with ΔPAZ_hDcr and ΔPPC_hDcr, compared to the respective reactions involving hDcr (please, compare Figure 3A,B). Next, we investigated the accumulation of the cleavage products released from the 5′-^32^P labeled substrates at different time points, i.e., 15 min, 30 min and 1h after addition of 50 nM protein to the reaction mixture. In the case of the RNA21 substrate, during the initial 15 min of incubations we observed 15-nt, 6-nt and 2-nt RNA products; however, the abundance of these products differed between reactions carried out with the wild type protein and the PAZ domain deletion variants (Figure 3A; right panel). Wild-type protein produced the most efficiently 6-nt RNAs, while ΔPAZ_hDcr and ΔPPC_hDcr generated the most efficiently 2-nt RNA products. With incubation time, for all proteins, we observed disappearance of 15-nt and 6-nt RNA products, and accumulation of 2-nt RNAs. Based on these results, we conclude that 2-nt RNAs were cut off from 15-nt and/or 6-nt RNAs. Consequently, the observed 15-nt and 6-nt RNAs can be defined as primary cleavage products, while 2-nt RNAs, as secondary cleavage products. In the case of the RNA32 substrate, irrespective of the applied protein, with incubation time we only observed accumulation of 4-nt RNA products (Figure 3B; right panel). Collected data indicate that both domain deletion variants, ΔPAZ_hDcr and ΔPPC_hDcr, exhibit similar RNA-cleavage properties with the same RNA substrates.

Altogether, the results presented here prove that the entire PPC region of hDicer, including the 3′-pocket and the 5′-pocket, is indispensable for generation of miRNA/siRNA-size products. Moreover, the collected data suggest that hDicer can further cleave miRNA/siRNA-size RNAs, and this process is more efficient when the PAZ domain of hDicer does not contribute to the substrate binding.

### 2.3. DNase Activity of Full-Length hDicer and the PAZ Domain Deletion Variants

**Figure 4:**
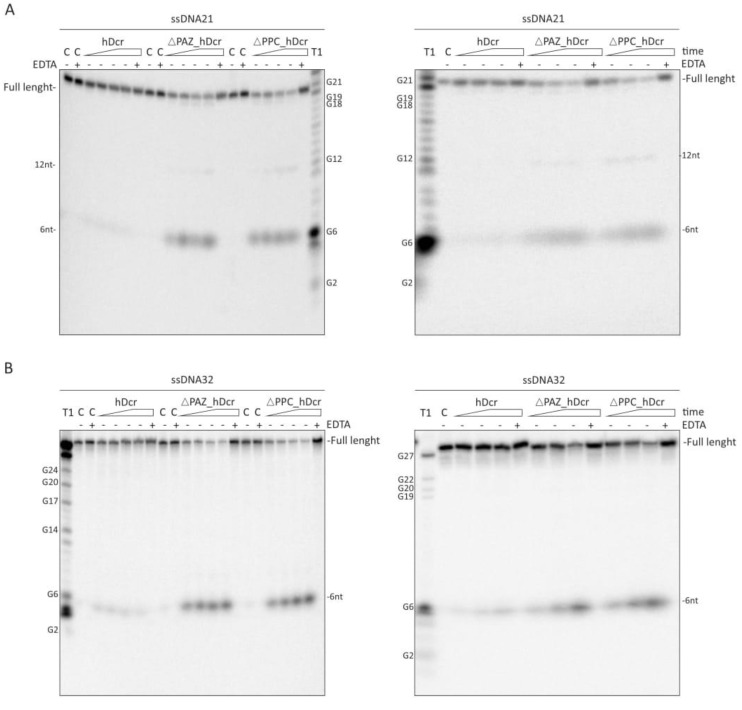
**Comparison of the DNase activity of full-length hDicer (hDcr) and the PAZ domain deletion variants ΔPAZ_hDcr and ΔPPC_hDcr on short ssDNA substrates.** (**A**,**B**; **left panels**) The results of the DNA-cleavage assays involving: 21-nt ssDNA (**A**; **left panel**) and 32-nt ssDNA (**B**; **left panel**), and increasing amounts of the respective protein (hDcr, ΔPAZ_hDcr or ΔPPC_hDcr): 12.5, 25, 50, 75 nM (represented by a triangle). (**A**,**B**; **right panels**) The results of the time-dependent cleavage of 21-nt ssDNA (**A**; **right panel**) and 32-nt ssDNA (**B**; **right panel**). Triangles represent time points: 15 min, 30 min, 1h. Reactions were carried out with 50 nM protein (hDcr, ΔPAZ_hDcr or ΔPPC_hDcr). (*C-*) controls incubated without a protein. (*C+EDTA*) controls incubated without a protein but with 25 mM EDTA. (*+EDTA*) supplementation of the reaction buffer with 25 mM EDTA. (T1) G-ladder generated with RNase T1.

The truncated form of *C. elegans* Dicer lacking the N-terminal domains, including the PPC region, exhibits a DNase activity [31]. Consequently, we asked whether the hDicer variants lacking the pre-miRNA and dsRNA substrate recognition domains, could also display a DNase activity. To test this hypothesis, we conducted the DNase cleavage assays, in which we applied DNA counterparts of RNA21 and RNA32; i.e., DNA21 and DNA32. We found that in reactions with DNA21 (Figure 4A), 6-nt products were efficiently generated by ΔPAZ_hDcr and ΔPPC_hDcr, and much less efficiently by hDcr. These products were not seen in all control reactions, including the control with 25 mM EDTA (Figure 4A; left panel). We also tested accumulation of 6-nt products at different time points, i.e., 15 min, 30 min and 1h, after 50 nM protein was added to the reaction mixture. In reactions with ΔPAZ_hDcr and ΔPPC_hDcr, 6-nt products increased with time, and we almost did not see these products in reactions with hDcr (Figure 4A; right panel). Interestingly, contrary to the reactions carried out with RNA21, we did not detect 2-nt products. Weak bands, representing 12-nt long DNA products, can be observed for reactions carried out with ΔPAZ_hDcr and ΔPPC_hDcr. For DNA32 substrate (Figure 4B), we observed 6-nt cleavage products in reactions with ΔPAZ_hDcr and ΔPPC_hDcr. The 6-nt DNA products were inefficiently generated by hDcr (Figure 4B; left panel). We also found that 6-nt products accumulated with time (Figure 4B; right panel). These short products did not appear in all control reactions. Collected results suggest that a putative DNase activity of hDicer, on ssDNA substrates, increases when its PAZ domain is not available for substrate binding. The same conclusions can apply to the RNase activity of hDicer on short ssRNA substrates (please, compare Figure 3 and Figure 4).

Collectively, our results suggest that domains other than PAZ may be involved in binding of non-canonical Dicer substrates like short ssRNAs and ssDNAs. Presumably, the hDicer domain that recognizes and binds a nucleic acid molecule, dictates the substrate cleavage pattern.

## 3. Discussion

The human Dicer-coding gene (*DICER1*) is located on chromosome 14 and comprises 29 exons [39]. *DICER1* is predicted to produce multiple hDicer transcript variants as a result of the initiation of transcription from alternative promoters and alternative splicing [39,40,41,42]. So far, four mRNA variants have been found to encode full-length hDicer protein (comprising 1922-amino acid residues) (https://www.ensembl.org/Homo_sapiens/Gene/Summary?db=core;g=ENSG00000100697;r=14:95086228-95158010). These four mRNA variants differ in their 5ʹ and 3′-untranslated regions, while their coding regions remain unchanged. Moreover, numerous shorter variants have been identified. One of the examples is Dicer1e transcript variant encoding an 820 amino acid (93-kDa) truncated form of hDicer [43]. This truncated hDicer variant comprises only RNase IIIa and IIIb domains, and the dsRBD. Although the biochemical activity of the Dicer1e variant protein has not been yet elucidated, a similarly engineered hDicer variant, containing both RNase III domains and dsRBD, has been found to generate in vitro ~15-nt RNA products from pre-miRNA and dsRNA substrates [30]. Additionally, it has been demonstrated that association of hDicer and 5-lipooxygenase, an enzyme involved in the biosynthesis of inflammatory mediators, modifies hDicer processing specificity towards pre-miRNAs, favoring production of ~55-nt and ~10 to 12-nt long RNA species [29]. It has also been shown that a truncated variant of hDicer containing the RNase IIIb domain and dsRBD, can produce ~55-nt and ~10-12-nt RNAs [29]. Interestingly, a truncated Dicer form comprising only one complete RNase III domain and dsRBD is produced in *C. elegans* by the cell death protease CED-3 [31]. Cleavage of *C. elegans* Dicer by CED-3 abolishes Dicer’s RNase activity. Instead, such a truncated Dicer variant displays a DNase activity and produces 3ʹ hydroxyl DNA breaks on chromosomes, which promotes apoptosis. The DNase activity of *C. elegans* Dicer [31], similarly as the cleavage activity of the RNase III-type nucleases [44], has been shown to depend on Mg^2+^ cations. A common characteristic of all abovementioned Dicer variants is the lack of the PAZ domain, which is required for anchoring Dicer canonical substrates. Here, we demonstrated that in vitro-generated hDicer variants lacking the PAZ domain are not able to process pre-miRNA and dsRNA substrates (Figure 2). However, such hDicer variants can cleave miRNA/siRNA-size RNAs to shorter fragments (Figure 3). Although we cannot yet explain the molecular mechanism that leads to production of such short RNA species, and the significance of this observation, we did observe that their generation was Mg^2+^-dependent, suggesting an RNase III-dependent mechanism. Based on the collected results, we can speculate that Dicer is involved in the miRNA turnover by promoting degradation of miRNAs, and the PPC region of Dicer is not involved in this process. Moreover, short RNA species (12-nt-long and shorter) have been shown to be generated by Dicer along the miRNA pathway [45]. Such truncated miRNA species have been suggested to compete with mature miRNAs for binding sites within target mRNAs.

The PPC cassette of hDicer has been shown to bind ~20-nt RNA and ~20-bp dsRNA substrates with high affinity (a Kd value of ~20 nM) [27], whereas other hDicer domains bind similar substrates with much lower affinity; i.e., hDicer DUF283 binds ~20-nt RNAs with a Kd value of ~10  μM [46], and hDicer dsRBD binds ~20-bp dsRNA with a Kd value 6.5 μM< [47,48]. Therefore, when the PAZ domain is present in Dicer, it predominantly contributes to substrate binding, that in a consequence results in generation of miRNA/siRNA-size products. However, when the PAZ domain is absent, or it is masked by other proteins [29], other Dicer domains can contribute to substrate binding. In this case, non-canonical products can be generated. Under specific circumstances, the removal of some domains from hDicer or masking their activities by other factors, can activate a DNase activity. The results of the initial studies presented in this manuscript strongly imply that human ribonuclease Dicer has a potential to act as a DNase.

## 4. Materials and Methods

### 4.1. Oligonucleotides

DNA and RNA oligonucleotides (Table 1) were purchased from Genomed (Warsaw, Poland) and FutureSynthesis (Poznan, Poland), respectively.

### 4.2. The 5ʹ-end Labeling of Oligonucleotides

Nucleic acid molecules were radiolabeled by phosphorylation of the 5ʹ end by using T4 Polynucleotide Kinase (Thermo Fisher Scientific, Waltham, MA, USA.) and γ(^32^P) ATP (Hartman Analytic, Braunschweig, Germany). The composition and reaction conditions were in accordance with the manufacturer’s instructions. Before preparing the reaction mixture, the RNA or DNA was denatured by heating to 90 °C and rapidly cooling on ice. The reaction products were separated on an 8% polyacrylamide gel under denaturing conditions. The cut-out gel fragments containing the desired length of RNA or DNA were ground, transferred to 400 μL 0.3 M sodium acetate (pH 5.0) and incubated for 16 h at 4 °C with gentle mixing. The samples were then centrifuged at 13,000 rpm for 1 min at 4 °C, and the supernatant was transferred to a new tube. RNA present in the supernatant was precipitated by the addition of 3 volumes of 96% ethanol (−20 °C) and freezing at −20 °C, overnight. Then, the preparations were centrifuged at 13,000 rpm for 30 min at 4 °C; the solution was decanted, and the pellet was suspended in 200 μL of 70% ethanol (−20 °C). The samples were centrifuged again at 13,000 rpm for 30 min at 4 °C. After removing the ethanol, the precipitate was dried and dissolved in RNase-free water. The amount of the radiolabeled oligonucleotide was adjusted to 10,000 cpm/μL. The homogeneity of the material after gel purification was checked by electrophoresis in 15% PAA under denaturing conditions. Gels were exposed to a phosphorimager plate, which was subsequently scanned with FLA-5100 Fluorescent Image Analyzer (Fujifilm, Minato, Tokyo, Japan) to visualize the bands.

### 4.3. Preparation of dsRNA

To prepare dsRNA substrates, non-labeled strand (RNA32_sense) was hybridized, at a molar ratio of approximately 1:1, with ^32^P-labeled complementary strand (RNA32_ov or RNA32_bl) in buffer containing 50 mM NaCl, 2.5 mM MgCl2 and 20 mM Tris-HCl, pH 7.5, by heating up to 95 °C and then slowly cooling down to room temperature. Next, the reaction mixtures were analyzed on 10% native polyacrylamide gels to check whether the double-stranded complexes were free of single-stranded species.

### 4.4. Preparation of Expression Plasmids

All primers were designed based on the full-length cDNA encoding transcript variant 2 of *DICER1* (NM_030621.4). The sequence of full-length hDicer was amplified using following primers: hDicer-F (forward) and hDicer-R (reverse). In order to obtain cDNA of two hDicer variants, we used two step-PCR amplification. In the first step, we performed PCR reactions using following primer sets, (i) for ΔPAZ_hDcr: hDicer-F (forward I) and ΔPAZ-R (reverse I), ΔPAZ-F (forward II) and hDicer-R (reverse II); (ii) for ΔPPC_hDcr: hDicer-F (forward I) and ΔPPC-R (reverse I), ΔPPC-F (forward II) and hDicer-R (reverse II). In the second step, the PCR products obtained in the first step were used as templates in an overlap extension PCR reaction (OE-PCR) [49]. All PCR amplifications involved *Pfu* Ultra II Fusion polymerase (Agilent, Santa Clara, CA, USA.).

The obtained PCR products were applied to prepare expression plasmids using SureVector system (Agilent), according to the manufacturer’s instructions. SureVector included sequences encoding the CMV promoter and the 3×Flag fusion peptide (at the C-terminus of the protein). All prepared constructs, encoding: hDcr, ΔPAZ_hDcr and ΔPPC_hDcr, were sequenced by Sanger method (Genomed, Warsaw, Poland). The results of sequencing reactions are presented in Figure S1.

### 4.5. Cell Culture and Transfection

293T NoDice cells [33] were cultured in DMEM (Gibco, Thermo Fisher Scientific, Waltham, MA, USA.) supplemented with 10% FBS (Gibco), Penicillin-Streptomycin (100 U/mL of penicillin and 100 µg/mL of streptomycin, Gibco) and 1 mM Sodium Pyruvate (Gibco), as described in Bogerd et al. [33]. Transfection was carried out by DharmaFECT kb DNA Transfection Reagent (Dharmacon, Lafayette, CO, USA.), according to the manufacturer’s instructions. 293T NoDice cell lines were kindly provided by Prof. Bryan R. Cullen.

### 4.6. Immunoprecipitation

Cells were harvested after 72 h by centrifugation at 1500 rpm for 3 min and suspended in lysis buffer (30 mM Hepes pH 7.4, 100 mM KCl, 5 mM MgCl2, 10% glycerol, 0,5 mM DTT and 0.2% Tergitol) containing 1× protease inhibitor mix without EDTA (Roche, Warsaw, Poland) and broken by passing through a 0.9 × 40 mm needle. Lysates were centrifuged at 13,000 rpm for 5 min at 4 °C. Supernatant was incubated overnight on a rotator with ANTI-FLAG^®^ M2 Affinity Gel (Merck, Darmstadt, Germany) that was pre-washed with TBS buffer. After incubation the beads were washed five times with TBS buffer. Isolated proteins were eluted with 100 μg/mL 3XFLAG Peptide (Sigma, Kawasaki, Japan). Purified proteins were suspended in buffer (20 mM Tris pH 7.5, 50 mM NaCl, 10% glycerol, and 0.25% Triton X-100). Protein concentration was estimated by the Bradford assay (BioRad, Irvine, CA, USA.), relative to a BSA standard curve, and on SDS-PAGE with BSA as a standard.

### 4.7. Western Blot Analysis

Obtained proteins were separated on 8% SDS-PAGE and electro-transferred onto a PVDF membrane (Thermo Fisher Scientific, Waltham, MA, USA.). For hDicer detection, the blots were probed with a mouse monoclonal primary anti-Dicer antibody mapping at the C-terminus of hDicer (1:300, Santa Cruz Biotechnology, Dallas, TX, USA.) and subsequently with HRP-conjugated secondary antibody, anti-mouse (1:1000, Jackson ImmunoResearch Laboratories, Inc., Cambridgeshire, UK). The immunoreactions were detected using SuperSignal^TM^ West Pico PLUS Chemiluminescent Substrat (Thermo Fisher Scientific, Waltham, MA, USA.).

### 4.8. hDicer Cleavage Assay

The cleavage assay was performed in 10 μL volumes in buffer containing 50 mM NaCl, 2.5 mM MgCl2 and 20 mM Tris-HCl, pH 7.5. The reaction mixture included ~5 nM ^32^P-labeled substrate and dilutions of the protein preparation (12.5, 25, 50, 75 nM). In addition, a reaction mixture without protein was prepared as a control. In controls including EDTA, the reaction buffer was supplemented with the chelating agent to the final concentration of 25 mM. Reactions were carried out for 30 min at 37 °C with the addition of the commercial RNase-inhibitor cocktail (NEB, Ipswich, USA.). The reactions were stopped by the addition of 1 volume of 10 M urea loading buffer (10 M urea dissolved in water at 50 °C) and heating for 5 min at 95 °C. Samples were separated on a 15% denaturing polyacrylamide gel 1×TBE running buffer.

### 4.9. Gel Imaging and Analysis

The data were collected using a Fujifilm FLA-5100 Fluorescent Image Analyzer and analyzed using MultiGauge 3.0 software (Fujifilm, Minato, Tokyo, Japan).

## Figures and Tables

**Table 1:** Oligonucleotide sequences.

Name	Sequence (5ʹ→3ʹ)
hDicer-F (1-20 nt)	CCTTGTTTAAACTTTAAGAGGAGGGCCACCATGAAAAGCCCTGCTTTGCA
hDicer-R (5749-5766 nt)	ACTTCCACCGCCTCCAGAACCTCCGCCACCGCTATTGGGAACCTGAGG
ΔPAZ-F (3127-3150 nt)	**TGACTCCAGCACTTTG**ATTCCAGCATCACTGTGGAGAAAA
ΔPAZ-R (2649-2670 nt)	**CAGTGATGCTGGAAT**CAAAGTGCTGGAGTCATTAACA
ΔPPC-F (3205-3223 nt)	**TACCCAAAAGCA**GAGCTAAGAGCCCAGACTG
ΔPPC-R (2238-2256 nt)	**GGCTCTTAGCTC**TGCTTTTGGGTAGCACTGC
DNA21	TCGAAGTATTCCGCGTACGTG
DNA32	ACCAGAACATGCAATGCAACTACAATGCACAT
pre-mir-16-1	UAGCAGCACGUAAAUAUUGGCGUUAAGAUUCUAAAAUUAUCUCCAGUAUUAACUGUGCUGCUGAA
RNA21	UCGAAGUAUUCCGCGUACGUG
RNA32_sense	GUGCAUUGUAGUUGCAUUGCAUGUUCUGGUCA
RNA32_bl	UGACCAGAACAUGCAAUGCAACUACAAUGCAC
RNA32_ov*	ACCAGAACAUGCAAUGCAACUACAAUGCACAU

Note: an asterisk (*) indicates the oligonucleotide used in the cleavage assays with a ssRNA substrate. The overlap sequences compatible with SureVector system are underlined. Overlapping sequences for OE-PCR are in bold. Nucleotide positions of primers in relation to the cDNA sequence of hDicer are indicated in brackets.

## Supplementary Materials

All the supplementary materials are available online.
