# Supplementary material for: Unknown Areas of Activity of Human Ribonuclease Dicer: A Putative Deoxyribonuclease Activity
Source: Molecules. 2020 Mar 20;25(6):1414. doi: 10.3390/molecules25061414 (PMC7144382; doi:10.3390/molecules25061414)
Supplement: Supplementary file 1 [file molecules-25-01414-s001.pdf]

SUPPLEMENTARY MATERIALS

Unknown areas of activity of human ribonuclease Dicer: a putative deoxyribonuclease activity

Marta Wojnicka [1], Agnieszka Szczepanska [1] and Anna Kurzynska-Kokorniak [1,*]

[1] Department of Ribonucleoprotein Biochemistry, Institute of Bioorganic Chemistry Polish Academy of Sciences, Poznan, 61-704, Poland

* Correspondence: akurzyns@ibch.poznan.pl

Figure S1. Analysis of sequencing data.

Sanger sequence analysis of the obtained genetic constructs: hDcr, ΔPAZ_hDcr and ΔPPC_hDcr in SureVector system. All primers used for sequencing were designed based on the full-length cDNA encoding transcript variant 2 of *DICER1* (NM_030621.4). Sequence data analysis was carried out using DNADynamo DNA Sequencing and Analysis Software. Note: alignment of the full-lenght hDcr sequence (pages:2-33), alignment of ΔPAZ_hDcr sequence (pages:34-65) and alignment of ΔPPC_hDcr sequence (pages:66-97). The reference sequence is full-lenght hDcr cDNA (indicated as *ref*).

```
                                   AatII
                             ZraI
ref/sequencing hDcr  CCAAGTCTCCACCCCATTGACGTCAATGGGAGTTTGTTTTGGCACCAAAATCAACGGGACTTTCCAAAATGTCGTAACAACTCCGCCCCATTGACGCAAA
                     P  S  L  H  P  I  D  V  N  G  S  L  F  W  H  Q  N  Q  R  D  F  P  K  C  R  N  N  S  A  P  L  T  Q

3_Seq1.ab1           CCAAGTCTCCACCCCATTGACGTCAATGGGAGTTTGTTTTGGCACCAAAATCAACGGGACTTTCCAAAATGTCGTAACAACTCCGCCCCATTGACGCAAA
kol3_1.ab1           - - - - - - - - - - - - - - - - - - - - - - - - - - - - - - - - - - - - - - - - - - - - - - - - - - -
kol3_P3.ab1          - - - - - - - - - - - - - - - - - - - - - - - - - - - - - - - - - - - - - - - - - - - - - - - - - - -
kol3_P4.ab1          - - - - - - - - - - - - - - - - - - - - - - - - - - - - - - - - - - - - - - - - - - - - - - - - - - -
kol3_P6.ab1          - - - - - - - - - - - - - - - - - - - - - - - - - - - - - - - - - - - - - - - - - - - - - - - - - - -
kol3_P8.ab1          - - - - - - - - - - - - - - - - - - - - - - - - - - - - - - - - - - - - - - - - - - - - - - - - - - -
kol3_P10.ab1         - - - - - - - - - - - - - - - - - - - - - - - - - - - - - - - - - - - - - - - - - - - - - - - - - - -
kol3_P12.ab1         - - - - - - - - - - - - - - - - - - - - - - - - - - - - - - - - - - - - - - - - - - - - - - - - - - -
3_P13.ab1            - - - - - - - - - - - - - - - - - - - - - - - - - - - - - - - - - - - - - - - - - - - - - - - - - - -
```

```
                                                                                           BglII
ref/sequencing hDcr  TGGGCGGTAGGCGTGTACGGTGGGAGGTCTATATAAGCAGAGCTGGTTTAGTGAACCGTCAGATCAGATCTTTGTCGATCCTACCATCCACTCGACACAC
                     M  G  G  R  R  V  R  W  E  V  Y  I  S  R  A  G  L  V  N  R  Q  I  R  S  L  S  I  L  P  S  T  R  H  T
                     Start

3_Seq1.ab1           TGGGCGGTAGGCGTGTACGGTGGGAGGTCTATATAAGCAGAGCTGGTTTAGTGAACCGTCAGATCAGATCTTTGTCGATCCTACCATCCACTCGACACAC
kol3_1.ab1           - - - - - - - - - - - - - - - - - - - - - - - - - - - - - - - - - - - - - - - - - - - - - - - - - - -
kol3_P3.ab1          - - - - - - - - - - - - - - - - - - - - - - - - - - - - - - - - - - - - - - - - - - - - - - - - - - -
kol3_P4.ab1          - - - - - - - - - - - - - - - - - - - - - - - - - - - - - - - - - - - - - - - - - - - - - - - - - - -
kol3_P6.ab1          - - - - - - - - - - - - - - - - - - - - - - - - - - - - - - - - - - - - - - - - - - - - - - - - - - -
kol3_P8.ab1          - - - - - - - - - - - - - - - - - - - - - - - - - - - - - - - - - - - - - - - - - - - - - - - - - - -
kol3_P10.ab1         - - - - - - - - - - - - - - - - - - - - - - - - - - - - - - - - - - - - - - - - - - - - - - - - - - -
kol3_P12.ab1         - - - - - - - - - - - - - - - - - - - - - - - - - - - - - - - - - - - - - - - - - - - - - - - - - - -
3_P13.ab1            - - - - - - - - - - - - - - - - - - - - - - - - - - - - - - - - - - - - - - - - - - - - - - - - - - -
```

```
                                                 SacI                    KpnI
                      NotI          HindIII    EcoICRI EcoRI        Acc65I           DraI
ref/sequencing hDcr  CCGCCAGCGGCCGCTGCCAAGCTTCCGAGCTCTCGAATTCAAAGGAGGTACCCACCCTTGTTTAAACTTTAAGAGGAGGGCCACCATGAAAAGCCCTGCT
                      R  Q  R  P  L  P  S  F  R  A  L  E  F  K  G  G  T  H  P  C  L  N  F  K  R  R  A  T  M  K  S  P  A

3_Seq1.ab1           CCGCCAGCGGCCGCTGCCAAGCTTCCGAGCTCTCGAATTCAAAGGAGGTACCCACCCTTGTTTAAACTTTAAGAGGAGGGCCACCATGAAAAGCCCTGCT
kol3_1.ab1           ----------------------------------------------------------------------------------------------------
kol3_P3.ab1          ----------------------------------------------------------------------------------------------------
kol3_P4.ab1          ----------------------------------------------------------------------------------------------------
kol3_P6.ab1          ----------------------------------------------------------------------------------------------------
kol3_P8.ab1          ----------------------------------------------------------------------------------------------------
kol3_P10.ab1         ----------------------------------------------------------------------------------------------------
kol3_P12.ab1         ----------------------------------------------------------------------------------------------------
3_P13.ab1            ----------------------------------------------------------------------------------------------------
```

```
                              Bpu10I       StuI  PstI                                              NcoI
ref/sequencing hDcr  TTGCAACCCCTCAGCATGGCAGGCCTGCAGCTCATGACCCCTGCTTCCTCACCAATGGGTCCTTTCTTTGGACTGCCATGGCAACAAGAAGCAATTCATG
                     L  Q  P  L  S  M  A  G  L  Q  L  M  T  P  A  S  S  P  M  G  P  F  F  G  L  P  W  Q  Q  E  A  I  H

3_Seq1.ab1           TTGCAACCCCTCAGCATGGCAGGCCTGCAGCTCATGACCCCTGCTTCCTCACCAATGGGTCCTTTCTTTGGACTGCCATGGCAACAAGAAGCAATTCATG
kol3_1.ab1           -----------------------------------GACCCCTGCTTCCTCACCAATGGGTCCTTTCTTTGGACTGCCATGGCAACAAGAAGCAATTCATG
kol3_P3.ab1          ----------------------------------------------------------------------------------------------------
kol3_P4.ab1          ----------------------------------------------------------------------------------------------------
kol3_P6.ab1          ----------------------------------------------------------------------------------------------------
kol3_P8.ab1          ----------------------------------------------------------------------------------------------------
kol3_P10.ab1         ----------------------------------------------------------------------------------------------------
kol3_P12.ab1         ----------------------------------------------------------------------------------------------------
3_P13.ab1            ----------------------------------------------------------------------------------------------------
```

```
                                                                                                DraI          Bpu10I
ref/sequencing hDcr  ATAACATTTATACGCCAAGAAAATATCAGGTTGAACTGCTTGAAGCAGCTCTGGATCATAATACCATCGTCTGTTTAAACACTGGCTCAGGGAAGACATT
                     D  N  I  Y  T  P  R  K  Y  Q  V  E  L  L  E  A  A  L  D  H  N  T  I  V  C  L  N  T  G  S  G  K  T  F

3_Seq1.ab1           ATAACATTTATACGCCAAGAAAATATCAGGTTGAACTGCTTGAAGCAGCTCTGGATCATAATACCATCGTCTGTTTAAACACTGGCTCAGGGAAGACATT
kol3_1.ab1           ATAACATTTATACGCCAAGAAAATATCAGGTTGAACTGCTTGAAGCAGCTCTGGATCATAATACCATCGTCTGTTTAAACACTGGCTCAGGGAAGACATT
kol3_P3.ab1          ----------------------------------------------------------------------------------------------------
kol3_P4.ab1          ----------------------------------------------------------------------------------------------------
kol3_P6.ab1          ----------------------------------------------------------------------------------------------------
kol3_P8.ab1          ----------------------------------------------------------------------------------------------------
kol3_P10.ab1         ----------------------------------------------------------------------------------------------------
kol3_P12.ab1         ----------------------------------------------------------------------------------------------------
3_P13.ab1            ----------------------------------------------------------------------------------------------------
```

```
                              ScaI
ref/sequencing hDcr  TATTGCAGTACTACTCACTAAAGAGCTGTCCTATCAGATCAGGGGAGACTTCAGCAGAAATGGAAAAAGGACGGTGTTCTTGGTCAACTCTGCAAACCAG
                       I  A  V  L  L  T  K  E  L  S  Y  Q  I  R  G  D  F  S  R  N  G  K  R  T  V  F  L  V  N  S  A  N  Q

3_Seq1.ab1           TATTGCAGTACTACTCACTAAAGAGCTGTCCTATCAGATCAGGGGAGACTTCAGCAGAAATGGAAAAAGGACGGTGTTCTTGGTCAACTCTGCAAACCAG
kol3_1.ab1           TATTGCAGTACTACTCACTAAAGAGCTGTCCTATCAGATCAGGGGAGACTTCAGCAGAAATGGAAAAAGGACGGTGTTCTTGGTCAACTCTGCAAACCAG
kol3_P3.ab1          ----------------------------------------------------------------------------------------------------
kol3_P4.ab1          ----------------------------------------------------------------------------------------------------
kol3_P6.ab1          ----------------------------------------------------------------------------------------------------
kol3_P8.ab1          ----------------------------------------------------------------------------------------------------
kol3_P10.ab1         ----------------------------------------------------------------------------------------------------
kol3_P12.ab1         ----------------------------------------------------------------------------------------------------
3_P13.ab1            ----------------------------------------------------------------------------------------------------
```

```
                     PvuII              BglII                                      NsiI
ref/sequencing hDcr GTTGCTCAACAAGTGTCAGCTGTCAGAACTCATTCAGATCTCAAGGTTGGGGAATACTCAAACCTAGAAGTAAATGCATCTTGGACAAAAGAGAGATGGA
                     V  A  Q  Q  V  S  A  V  R  T  H  S  D  L  K  V  G  E  Y  S  N  L  E  V  N  A  S  W  T  K  E  R  W

3_Seq1.ab1          GTTGCTCAACAAGTGTCAGCTGTCAGAACTCATTCAGATCTCAAGGTT----------------------------------------------------
kol3_1.ab1          GTTGCTCAACAAGTGTCAGCTGTCAGAACTCATTCAGATCTCAAGGTTGGGGAATACTCAAACCTAGAAGTAAATGCATCTTGGACAAAAGAGAGATGGA
kol3_P3.ab1         ----------------------------------------------------------------------------------------------------
kol3_P4.ab1         ----------------------------------------------------------------------------------------------------
kol3_P6.ab1         ----------------------------------------------------------------------------------------------------
kol3_P8.ab1         ----------------------------------------------------------------------------------------------------
kol3_P10.ab1        ----------------------------------------------------------------------------------------------------
kol3_P12.ab1        ----------------------------------------------------------------------------------------------------
3_P13.ab1           ----------------------------------------------------------------------------------------------------
```

```
ref/sequencing hDcr ACCAAGAGTTTACTAAGCACCAGGTTCTCATTATGACTTGCTATGTCGCCTTGAATGTTTTGAAAAATGGTTACTTATCACTGTCAGACATTAACCTTTT
                    N  Q  E  F  T  K  H  Q  V  L  I  M  T  C  Y  V  A  L  N  V  L  K  N  G  Y  L  S  L  S  D  I  N  L  L

3_Seq1.ab1          ----------------------------------------------------------------------------------------------------
kol3_1.ab1          ACCAAGAGTTTACTAAGCACCAGGTTCTCATTATGACTTGCTATGTCGCCTTGAATGTTTTGAAAAATGGTTACTTATCACTGTCAGACATTAACCTTTT
kol3_P3.ab1         ----------------------------------------------------------------------------------------------------
kol3_P4.ab1         ----------------------------------------------------------------------------------------------------
kol3_P6.ab1         ----------------------------------------------------------------------------------------------------
kol3_P8.ab1         ----------------------------------------------------------------------------------------------------
kol3_P10.ab1        ----------------------------------------------------------------------------------------------------
kol3_P12.ab1        ----------------------------------------------------------------------------------------------------
3_P13.ab1           ----------------------------------------------------------------------------------------------------
```

```
ref/sequencing hDcr GGTGTTTGATGAGTGTCATCTTGCAATCCTAGACCACCCCTATCGAGAAATTATGAAGCTCTGTGAAAATTGTCCATCATGTCCTCGCATTTTGGGACTA
                      V  F  D  E  C  H  L  A  I  L  D  H  P  Y  R  E  I  M  K  L  C  E  N  C  P  S  C  P  R  I  L  G  L

3_Seq1.abl         ----------------------------------------------------------------------------------------------------
kol3_1.abl         GGTGTTTGATGAGTGTCATCTTGCAATCCTAGACCACCCCTATCGAGAAATTATGAAGCTCTGTGAAAATTGTCCATCATGTCCTCGCATTTTGGGACTA
kol3_P3.abl        ----------------------------------------------------------------------------------------------------
kol3_P4.abl        ----------------------------------------------------------------------------------------------------
kol3_P6.abl        ----------------------------------------------------------------------------------------------------
kol3_P8.abl        ----------------------------------------------------------------------------------------------------
kol3_P10.abl       ----------------------------------------------------------------------------------------------------
kol3_P12.abl       ----------------------------------------------------------------------------------------------------
3_P13.abl          ----------------------------------------------------------------------------------------------------
```

```
                                  |DraI                                                           |AflII
ref/sequencing hDcr ACTGCTTCCATTTTAAATGGGAAATGTGATCCAGAGGAATTGGAAGAAAAGATTCAGAAACTAGAGAAAATTCTTAAGAGTAATGCTGAAACTGCAACTG
                     T  A  S  I  L  N  G  K  C  D  P  E  E  L  E  E  K  I  Q  K  L  E  K  I  L  K  S  N  A  E  T  A  T

3_Seq1.abl          ----------------------------------------------------------------------------------------------------
kol3_1.abl          ACTGCTTCCATTTTAAATGGGAAATGTGATCCAGAGGAATTGGAAGAAAAGATTCAGAAACTAGAGAAAATTCTTAAGAGTAATGCTGAAACTGCAACTG
kol3_P3.abl         ----------------------------------------------------------------------------------------------------
kol3_P4.abl         ----------------------------------------------------------------------------------------------------
kol3_P6.abl         ----------------------------------------------------------------------------------------------------
kol3_P8.abl         ----------------------------------------------------------------------------------------------------
kol3_P10.abl        ----------------------------------------------------------------------------------------------------
kol3_P12.abl        ----------------------------------------------------------------------------------------------------
3_P13.abl           ----------------------------------------------------------------------------------------------------
```

```
ref/sequencing hDcr ACCTGGTGGTCTTAGACAGGTATACTTCTCAGCCATGTGAGATTGTGGTGGATTGTGGACCATTTACTGACAGAAGTGGGCTTTATGAAAGACTGCTGAT
                    D  L  V  V  L  D  R  Y  T  S  Q  P  C  E  I  V  V  D  C  G  P  F  T  D  R  S  G  L  Y  E  R  L  L  M

3_Seq1.ab1          ---------------------------------------------------------------------------------------------------
kol3_1.ab1          ACCTGGTGGTCTTAGACAGGTATACTTCTCAGCCATGTGAGATTGTGGTGGATTGTGGACCATTTACTGACAGAAGTGGGCTTTATGAAAGACTGCTGAT
kol3_P3.ab1         ---------------------------------------------------------------------------------------------------
kol3_P4.ab1         ---------------------------------------------------------------------------------------------------
kol3_P6.ab1         ---------------------------------------------------------------------------------------------------
kol3_P8.ab1         ---------------------------------------------------------------------------------------------------
kol3_P10.ab1        ---------------------------------------------------------------------------------------------------
kol3_P12.ab1        ---------------------------------------------------------------------------------------------------
3_P13.ab1           ---------------------------------------------------------------------------------------------------
```

```
ref/sequencing hDcr GGAATTAGAAGAAGCACTTAATTTTATCAATGATTGTAATATATCTGTACATTCAAAAGAAAGAGATTCTACTTTAATTTCGAAACAGATACTATCAGAC
                     E  L  E  E  A  L  N  F  I  N  D  C  N  I  S  V  H  S  K  E  R  D  S  T  L  I  S  K  Q  I  L  S  D

3_Seq1.ab1          ---------------------------------------------------------------------------------------------------
kol3_1.ab1          GGAATTAGAAGAAGCACTTAATTTTATCAATGATTGTAATATATCTGTACATTCAAAAGAAAGAGATTCTACTTTAATTTCGAAACAGATACTATCAGAC
kol3_P3.ab1         ---------------------------------------------------------------------------------------------------
kol3_P4.ab1         ---------------------------------------------------------------------------------------------------
kol3_P6.ab1         ---------------------------------------------------------------------------------------------------
kol3_P8.ab1         ---------------------------------------------------------------------------------------------------
kol3_P10.ab1        ---------------------------------------------------------------------------------------------------
kol3_P12.ab1        ---------------------------------------------------------------------------------------------------
3_P13.ab1           ---------------------------------------------------------------------------------------------------
```

```
ref/sequencing hDcr TGTCGTGCCGTATTGGTAGTTCTGGGACCCTGGTGTGCAGATAAAGTAGCTGGAATGATGGTAAGAGAACTACAGAAATACATCAAACATGAGCAAGAGG
                     C  R  A  V  L  V  V  L  G  P  W  C  A  D  K  V  A  G  M  M  V  R  E  L  Q  K  Y  I  K  H  E  Q  E

3_Seq1.ab1         ----------------------------------------------------------------------------------------------------
kol3_1.ab1         TGTCGTGCCGTATTGGTAGTTCTGGGACCCTGGTGTGCAGATAAAGTAGCTGGAATGATGGTAAGAGAACTACAGAAATACATCAAACATGAGCAAGAGG
kol3_P3.ab1        -------------------------------------------------------TGATGGTAAGAGAACTACAGAAATACATCAAACATGAGCAAGAGG
kol3_P4.ab1        ----------------------------------------------------------------------------------------------------
kol3_P6.ab1        ----------------------------------------------------------------------------------------------------
kol3_P8.ab1        ----------------------------------------------------------------------------------------------------
kol3_P10.ab1       ----------------------------------------------------------------------------------------------------
kol3_P12.ab1       ----------------------------------------------------------------------------------------------------
3_P13.ab1          ----------------------------------------------------------------------------------------------------
```

```
                                                                           DraIII
ref/sequencing hDcr AGCTGCACAGGAAATTTTTATTGTTTACAGACACTTTCCTAAGGAAAATACATGCACTATGTGAAGAGCACTTCTCACCTGCCTCACTTGACCTGAAATT
                   E  L  H  R  K  F  L  L  F  T  D  T  F  L  R  K  I  H  A  L  C  E  E  H  F  S  P  A  S  L  D  L  K  F

3_Seq1.ab1         ----------------------------------------------------------------------------------------------------
kol3_1.ab1         AGCTGCACAGGAAATTTTTATTGTTTACAGACACTTTCCTAAGGAAAATACATGCACTATGTGAAGAGCACTTCTCACCTGCCTCACTTGACCTGAAATT
kol3_P3.ab1        AGCTGCACAGGAAATTTTTATTGTTTACAGACACTTTCCTAAGGAAAATACATGCACTATGTGAAGAGCACTTCTCACCTGCCTCACTTGACCTGAAATT
kol3_P4.ab1        ----------------------------------------------------------------------------------------------------
kol3_P6.ab1        ----------------------------------------------------------------------------------------------------
kol3_P8.ab1        ----------------------------------------------------------------------------------------------------
kol3_P10.ab1       ----------------------------------------------------------------------------------------------------
kol3_P12.ab1       ----------------------------------------------------------------------------------------------------
3_P13.ab1          ----------------------------------------------------------------------------------------------------
```

```
                                                                                           NdeI
ref/sequencing hDcr TGTAACTCCTAAAGTAATCAAACTGCTCGAAATCTTACGCAAATATAAACCATATGAGCGACAGCAGTTTGAAAGCGTTGAGTGGTATAATAATAGAAAT
                      V  T  P  K  V  I  K  L  L  E  I  L  R  K  Y  K  P  Y  E  R  Q  Q  F  E  S  V  E  W  Y  N  N  R  N

3_Seq1.ab1          ----------------------------------------------------------------------------------------------------
kol3_1.ab1          TGTAACTCCTAAAGTAATCAAACTGCTCGAAATCTTACGCAAATATAAACCATATGAGCGACAGCAGTTTGAAAGCGTTGAGTGGTATAATAATAGAAAT
kol3_P3.ab1         TGTAACTCCTAAAGTAATCAAACTGCTCGAAATCTTACGCAAATATAAACCATATGAGCGACAGCAGTTTGAAAGCGTTGAGTGGTATAATAATAGAAAT
kol3_P4.ab1         ----------------------------------------------------------------------------------------------------
kol3_P6.ab1         ----------------------------------------------------------------------------------------------------
kol3_P8.ab1         ----------------------------------------------------------------------------------------------------
kol3_P10.ab1        ----------------------------------------------------------------------------------------------------
kol3_P12.ab1        ----------------------------------------------------------------------------------------------------
3_P13.ab1           ----------------------------------------------------------------------------------------------------
```

```
ref/sequencing hDcr CAGGATAATTATGTGTCATGGAGTGATTCTGAGGATGATGATGAGGATGAAGAAATTGAAGAAAAAGAGAAGCCAGAGACAAATTTTCCTTCTCCTTTTA
                     Q  D  N  Y  V  S  W  S  D  S  E  D  D  D  E  D  E  E  I  E  E  K  E  K  P  E  T  N  F  P  S  P  F

3_Seq1.ab1          ----------------------------------------------------------------------------------------------------
kol3_1.ab1          CAGGATAATTATGTGTCATGGAGTGATTCTGAGGATGATGATGAGGATGAAGAAA---------------------------------------------
kol3_P3.ab1         CAGGATAATTATGTGTCATGGAGTGATTCTGAGGATGATGATGAGGATGAAGAAATTGAAGAAAAAGAGAAGCCAGAGACAAATTTTCCTTCTCCTTTTA
kol3_P4.ab1         ----------------------------------------------------------------------------------------------------
kol3_P6.ab1         ----------------------------------------------------------------------------------------------------
kol3_P8.ab1         ----------------------------------------------------------------------------------------------------
kol3_P10.ab1        ----------------------------------------------------------------------------------------------------
kol3_P12.ab1        ----------------------------------------------------------------------------------------------------
3_P13.ab1           ----------------------------------------------------------------------------------------------------
```

```
ref/sequencing hDcr CCAACATTTTGTGCGGAATTATTTTTGTGGAAAGAAGATACACAGCAGTTGTCTTAAACAGATTGATAAAGGAAGCTGGCAAACAAGATCCAGAGCTGGC
                   T  N  I  L  C  G  I  I  F  V  E  R  R  Y  T  A  V  V  L  N  R  L  I  K  E  A  G  K  Q  D  P  E  L  A

3_Seq1.ab1         ----------------------------------------------------------------------------------------------------
kol3_1.ab1         ----------------------------------------------------------------------------------------------------
kol3_P3.ab1        CCAACATTTTGTGCGGAATTATTTTTGTGGAAAGAAGATACACAGCAGTTGTCTTAAACAGATTGATAAAGGAAGCTGGCAAACAAGATCCAGAGCTGGC
kol3_P4.ab1        -----------------------------------------------------------------------------------CAAGATCCAGAGCTGGC
kol3_P6.ab1        ----------------------------------------------------------------------------------------------------
kol3_P8.ab1        ----------------------------------------------------------------------------------------------------
kol3_P10.ab1       ----------------------------------------------------------------------------------------------------
kol3_P12.ab1       ----------------------------------------------------------------------------------------------------
3_P13.ab1          ----------------------------------------------------------------------------------------------------
```

```
                                                                                             EcoRI
ref/sequencing hDcr TTATATCAGTAGCAATTTCATAACTGGACATGGCATTGGGAAGAATCAGCCTCGCAACAAACAGATGGAAGCAGAATTCAGAAAACAGGAAGAGGTACTT
                     Y  I  S  S  N  F  I  T  G  H  G  I  G  K  N  Q  P  R  N  K  Q  M  E  A  E  F  R  K  Q  E  E  V  L

3_Seq1.ab1         ----------------------------------------------------------------------------------------------------
kol3_1.ab1         ----------------------------------------------------------------------------------------------------
kol3_P3.ab1        TTATATCAGTAGCAATTTCATAACTGGACATGGCATTGGGAAGAATCAGCCTCGCAACAAACAGATGGAAGCAGAATTCAGAAAACAGGAAGAGGTACTT
kol3_P4.ab1        TTATATCAGTAGCAATTTCATAACTGGACATGGCATTGGGAAGAATCAGCCTCGCAACAAACAGATGGAAGCAGAATTCAGAAAACAGGAAGAGGTACTT
kol3_P6.ab1        ----------------------------------------------------------------------------------------------------
kol3_P8.ab1        ----------------------------------------------------------------------------------------------------
kol3_P10.ab1       ----------------------------------------------------------------------------------------------------
kol3_P12.ab1       ----------------------------------------------------------------------------------------------------
3_P13.ab1          ----------------------------------------------------------------------------------------------------
```

```
ref/sequencing hDcr AGGAAATTTCGAGCACATGAGACCAACCTGCTTATTGCAACAAGTATTGTAGAAGAGGGTGTTGATATACCAAAATGCAACTTGGTGGTTCGTTTTGATT
                     R  K  F  R  A  H  E  T  N  L  L  I  A  T  S  I  V  E  E  G  V  D  I  P  K  C  N  L  V  V  R  F  D

3_Seq1.ab1         ----------------------------------------------------------------------------------------------------
kol3_1.ab1         ----------------------------------------------------------------------------------------------------
kol3_P3.ab1        AGGAAATTTCGAGCACATGAGACCAACCTGCTTATTGCAACAAGTATTGTAGAAGAGGGTGTTGATATACCAAAATGCAACTTGGTGGTTCGTTTTGATT
kol3_P4.ab1        AGGAAATTTCGAGCACATGAGACCAACCTGCTTATTGCAACAAGTATTGTAGAAGAGGGTGTTGATATACCAAAATGCAACTTGGTGGTTCGTTTTGATT
kol3_P6.ab1        ----------------------------------------------------------------------------------------------------
kol3_P8.ab1        ----------------------------------------------------------------------------------------------------
kol3_P10.ab1       ----------------------------------------------------------------------------------------------------
kol3_P12.ab1       ----------------------------------------------------------------------------------------------------
3_P13.ab1          ----------------------------------------------------------------------------------------------------
```

```
                                 |ClaI*
ref/sequencing hDcr TGCCCACAGAATATCGATCCTATGTTCAATCTAAAGGAAGAGCAAGGGCACCCATCTCTAATTATATAATGTTAGCGGATACAGACAAAATAAAAAGTTT
                   L  P  T  E  Y  R  S  Y  V  Q  S  K  G  R  A  R  A  P  I  S  N  Y  I  M  L  A  D  T  D  K  I  K  S  F

3_Seq1.ab1         ----------------------------------------------------------------------------------------------------
kol3_1.ab1         ----------------------------------------------------------------------------------------------------
kol3_P3.ab1        TGCCCACAGAATATCGATCCTATGTTCAATCTAAAGGAAGAGCAAGGGCACCCATCTCTAATTATATAATGTTAGCGGATACAGACAAAATAAAAAGTTT
kol3_P4.ab1        TGCCCACAGAATATCGATCCTATGTTCAATCTAAAGGAAGAGCAAGGGCACCCATCTCTAATTATATAATGTTAGCGGATACAGACAAAATAAAAAGTTT
kol3_P6.ab1        ----------------------------------------------------------------------------------------------------
kol3_P8.ab1        ----------------------------------------------------------------------------------------------------
kol3_P10.ab1       ----------------------------------------------------------------------------------------------------
kol3_P12.ab1       ----------------------------------------------------------------------------------------------------
3_P13.ab1          ----------------------------------------------------------------------------------------------------
```

```
                                                   BglII
ref/sequencing hDcr TGAAGAAGACCTTAAAACCTACAAAGCTATTGAAAAGATCTTGAGAAACAAGTGTTCCAAGTCGGTTGATACTGGTGAGACTGACATTGATCCTGTCATG
                     E  E  D  L  K  T  Y  K  A  I  E  K  I  L  R  N  K  C  S  K  S  V  D  T  G  E  T  D  I  D  P  V  M

3_Seq1.ab1          ----------------------------------------------------------------------------------------------------
kol3_1.ab1          ----------------------------------------------------------------------------------------------------
kol3_P3.ab1         TGAAGAAGACCTTAAA------------------------------------------------------------------------------------
kol3_P4.ab1         TGAAGAAGACCTTAAAACCTACAAAGCTATTGAAAAGATCTTGAGAAACAAGTGTTCCAAGTCGGTTGATACTGGTGAGACTGACATTGATCCTGTCATG
kol3_P6.ab1         ----------------------------------------------------------------------------------------------------
kol3_P8.ab1         ----------------------------------------------------------------------------------------------------
kol3_P10.ab1        ----------------------------------------------------------------------------------------------------
kol3_P12.ab1        ----------------------------------------------------------------------------------------------------
3_P13.ab1           ----------------------------------------------------------------------------------------------------
```

```
                                            NdeI        StuI
ref/sequencing hDcr GATGATGATGACGTTTTCCCACCATATGTGTTGAGGCCTGACGATGGTGGTCCACGAGTCACAATCAACACGGCCATTGGACACATCAATAGATACTGTG
                     D  D  D  D  V  F  P  P  Y  V  L  R  P  D  D  G  G  P  R  V  T  I  N  T  A  I  G  H  I  N  R  Y  C

3_Seq1.ab1          ----------------------------------------------------------------------------------------------------
kol3_1.ab1          ----------------------------------------------------------------------------------------------------
kol3_P3.ab1         ----------------------------------------------------------------------------------------------------
kol3_P4.ab1         GATGATGATGACGTTTTCCCACCATATGTGTTGAGGCCTGACGATGGTGGTCCACGAGTCACAATCAACACGGCCATTGGACACATCAATAGATACTGTG
kol3_P6.ab1         ----------------------------------------------------------------------------------------------------
kol3_P8.ab1         ----------------------------------------------------------------------------------------------------
kol3_P10.ab1        ----------------------------------------------------------------------------------------------------
kol3_P12.ab1        ----------------------------------------------------------------------------------------------------
3_P13.ab1           ----------------------------------------------------------------------------------------------------
```

ref/sequencing hDcr	CTAGATTACCAAGTGATCCGTTTACTCATCTAGCTCCTAAATGCAGAACCCGAGAGTTGCCTGATGGTACATTTTATTCAACTCTTTATCTGCCAATTAA
	A R L P S D P F T H L A P K C R T R E L P D G T F Y S T L Y L P I N
3_Seq1.ab1	--
kol3_1.ab1	--
kol3_P3.ab1	--
kol3_P4.ab1	CTAGATTACCAAGTGATCCGTTTACTCATCTAGCTCCTAAATGCAGAACCCGAGAGTTGCCTGATGGTACATTTTATTCAACTCTTTATCTGCCAATTAA
kol3_P6.ab1	--
kol3_P8.ab1	--
kol3_P10.ab1	--
kol3_P12.ab1	--
3_P13.ab1	--

ref/sequencing hDcr	CTCACCTCTTCGAGCCTCCATTGTTGGTCCACCAATGAGCTGTGTACGATTGGCTGAAAGAGTTGTAGCTCTCATTTGCTGTGAGAAACTGCACAAAATT
	S P L R A S I V G P P M S C V R L A E R V V A L I C C E K L H K I
3_Seq1.ab1	--
kol3_1.ab1	--
kol3_P3.ab1	--
kol3_P4.ab1	CTCACCTCTTCGAGCCTCCATTGTTGGTCCACCAATGAGCTGTGTACGATTGGCTGAAAGAGTTGTAGCTCTCATTTGCTGTGAGAAACTGCACAAAATT
kol3_P6.ab1	--
kol3_P8.ab1	--
kol3_P10.ab1	--
kol3_P12.ab1	--
3_P13.ab1	--

```
ref/sequencing hDcr GGCGAACTGGATGACCATTTGATGCCAGTTGGGAAAGAGACTGTTAAATATGAAGAGGAGCTTGATTTGCATGATGAAGAAGAGACCAGTGTTCCAGGAA
                    G  E  L  D  D  H  L  M  P  V  G  K  E  T  V  K  Y  E  E  E  L  D  L  H  D  E  E  E  T  S  V  P  G

3_Seq1.ab1          ----------------------------------------------------------------------------------------------------
kol3_1.ab1          ----------------------------------------------------------------------------------------------------
kol3_P3.ab1         ----------------------------------------------------------------------------------------------------
kol3_P4.ab1         GGCGAACTGGATGACCATTTGATGCCAGTTGGGAAAGAGACTGTTAAATATGAAGAGGAGCTTGATTTGCATGATGAAGAAGAGACCAGTGTTCCAGGAA
kol3_P6.ab1         ----------------------------------------------------------------------------------------------------
kol3_P8.ab1         ----------------------------------------------------------------------------------------------------
kol3_P10.ab1        ----------------------------------------------------------------------------------------------------
kol3_P12.ab1        ----------------------------------------------------------------------------------------------------
3_P13.ab1           ----------------------------------------------------------------------------------------------------
```

```
                                                                                        BclI*
ref/sequencing hDcr GACCAGGTTCCACGAAACGAAGGCAGTGCTACCCAAAAGCAATTCCAGAGTGTTTGAGGGATAGTTATCCCAGACCTGATCAGCCCTGTTACCTGTATGT
                    R P  G  S  T  K  R  R  Q  C  Y  P  K  A  I  P  E  C  L  R  D  S  Y  P  R  P  D  Q  P  C  Y  L  Y  V

3_Seq1.ab1          ----------------------------------------------------------------------------------------------------
kol3_1.ab1          ----------------------------------------------------------------------------------------------------
kol3_P3.ab1         ----------------------------------------------------------------------------------------------------
kol3_P4.ab1         GACCAGGTTCCACGAAACGAAGGCAGTGCTACCCAAAAGCAATTCCAGAGTGTTTGAGGGATAGTTATCCCAGACCTGATCAGCCCTGTTACCTGTATGT
kol3_P6.ab1         ----------------------------------------------------------------------------------------------------
kol3_P8.ab1         ----------------------------------------------------------------------------------------------------
kol3_P10.ab1        ----------------------------------------------------------------------------------------------------
kol3_P12.ab1        ----------------------------------------------------------------------------------------------------
3_P13.ab1           ----------------------------------------------------------------------------------------------------
```

```
ref/sequencing hDcr GATAGGAATGGTTTTAACTACACCTTTACCTGATGAACTCAACTTTAGAAGGCGGAAGCTCTATCCTCCTGAAGATACCACAAGATGCTTTGGAATACTG
                   I  G  M  V  L  T  T  P  L  P  D  E  L  N  F  R  R  R  K  L  Y  P  P  E  D  T  T  R  C  F  G  I  L

3_Seq1.abl         ----------------------------------------------------------------------------------------------------
kol3_1.abl         ----------------------------------------------------------------------------------------------------
kol3_P3.abl        ----------------------------------------------------------------------------------------------------
kol3_P4.abl        GATAGGAATGGTTTTAACTACACCTTTACCTGATGAACTCAACTTTAGAAGGCGGAAGCTCTATCCTCCTGAAGATACCACAAGATGCTTTGGAATACTG
kol3_P6.abl        -----------------------------------------------------------------------------------------------TACTG
kol3_P8.abl        ----------------------------------------------------------------------------------------------------
kol3_P10.abl       ----------------------------------------------------------------------------------------------------
kol3_P12.abl       ----------------------------------------------------------------------------------------------------
3_P13.abl          ----------------------------------------------------------------------------------------------------
```

```
                                                                                  BstEII
ref/sequencing hDcr ACGGCCAAACCCATACCTCAGATTCCACACTTTCCTGTGTACACACGCTCTGGAGAGGTTACCATATCCATTGAGTTGAAGAAGTCTGGTTTCATGTTGT
                    T  A  K  P  I  P  Q  I  P  H  F  P  V  Y  T  R  S  G  E  V  T  I  S  I  E  L  K  K  S  G  F  M  L

3_Seq1.abl         ----------------------------------------------------------------------------------------------------
kol3_1.abl         ----------------------------------------------------------------------------------------------------
kol3_P3.abl        ----------------------------------------------------------------------------------------------------
kol3_P4.abl        ACGGCCAAACCCATACCTCAGATTCCACACTTTCCTGTGTACACACGCTCTGGAGAGGTTACCATATCCATTGAGTTGAAGAAGTCTGGTTTCATGTTGT
kol3_P6.abl        ACGGCCAAACCCATACCTCAGATTCCACACTTTCCTGTGTACACACGCTCTGGAGAGGTTACCATATCCATTGAGTTGAAGAAGTCTGGTTTCATGTTGT
kol3_P8.abl        ----------------------------------------------------------------------------------------------------
kol3_P10.abl       ----------------------------------------------------------------------------------------------------
kol3_P12.abl       ----------------------------------------------------------------------------------------------------
3_P13.abl          ----------------------------------------------------------------------------------------------------
```

```
                                                                                                       DraI
ref/sequencing hDcr CTCTACAAATGCTTGAGTTGATTACAAGACTTCACCAGTATATATTCTCACATATTCTTCGGCTTGAAAAACCTGCACTAGAATTTAAACCTACAGACGC
                     S  L  Q  M  L  E  L  I  T  R  L  H  Q  Y  I  F  S  H  I  L  R  L  E  K  P  A  L  E  F  K  P  T  D  A

3_Seq1.ab1          ----------------------------------------------------------------------------------------------------
kol3_1.ab1          ----------------------------------------------------------------------------------------------------
kol3_P3.ab1         ----------------------------------------------------------------------------------------------------
kol3_P4.ab1         CTCTACAAATGCTTGAGTTGATTACAAGACTTCACCAGTATATATTCTCACATATTCTTCGGCTTGAAAAACCTGCACTAGAATTTAAACCTACAGACGC
kol3_P6.ab1         CTCTACAAATGCTTGAGTTGATTACAAGACTTCACCAGTATATATTCTCACATATTCTTCGGCTTGAAAAACCTGCACTAGAATTTAAACCTACAGACGC
kol3_P8.ab1         ----------------------------------------------------------------------------------------------------
kol3_P10.ab1        ----------------------------------------------------------------------------------------------------
kol3_P12.ab1        ----------------------------------------------------------------------------------------------------
3_P13.ab1           ----------------------------------------------------------------------------------------------------
```

```
                                                                       BstXI           DraI
ref/sequencing hDcr TGATTCAGCATACTGTGTTCTACCTCTTAATGTTGTTAATGACTCCAGCACTTTGGATATTGACTTTAAATTCATGGAAGATATTGAGAAGTCTGAAGCT
                      D  S  A  Y  C  V  L  P  L  N  V  V  N  D  S  S  T  L  D  I  D  F  K  F  M  E  D  I  E  K  S  E  A

3_Seq1.ab1          ----------------------------------------------------------------------------------------------------
kol3_1.ab1          ----------------------------------------------------------------------------------------------------
kol3_P3.ab1         ----------------------------------------------------------------------------------------------------
kol3_P4.ab1         TGATTCAGCATACTG-------------------------------------------------------------------------------------
kol3_P6.ab1         TGATTCAGCATACTGTGTTCTACCTCTTAATGTTGTTAATGACTCCAGCACTTTGGATATTGACTTTAAATTCATGGAAGATATTGAGAAGTCTGAAGCT
kol3_P8.ab1         ----------------------------------------------------------------------------------------------------
kol3_P10.ab1        ----------------------------------------------------------------------------------------------------
kol3_P12.ab1        ----------------------------------------------------------------------------------------------------
3_P13.ab1           ----------------------------------------------------------------------------------------------------
```

BsmI DraI EcoRV

ref/sequencing hDcr CGCATAGGCATTCCCAGTACAAAGTATACAAAAGAAACACCCTTTGTTTTTAAATTAGAAGATTACCAAGATGCCGTTATCATTCCAAGATATCGCAATT

R I G I P S T K Y T K E T P F V F K L E D Y Q D A V I I P R Y R N

3_Seq1.ab1 --
kol3_1.ab1 --
kol3_P3.ab1 --
kol3_P4.ab1 --
kol3_P6.ab1 CGCATAGGCATTCCCAGTACAAAGTATACAAAAGAAACACCCTTTGTTTTTAAATTAGAAGATTACCAAGATGCCGTTATCATTCCAAGATATCGCAATT
kol3_P8.ab1 --
kol3_P10.ab1 --
kol3_P12.ab1 --
3_P13.ab1 --

BclI* ClaI SspI

ref/sequencing hDcr TTGATCAGCCTCATCGATTTTATGTAGCTGATGTGTACACTGATCTTACCCCACTCAGTAAATTTCCTTCCCCTGAGTATGAAACTTTTGCAGAATATTA

F D Q P H R F Y V A D V Y T D L T P L S K F P S P E Y E T F A E Y Y

3_Seq1.ab1 --
kol3_1.ab1 --
kol3_P3.ab1 --
kol3_P4.ab1 --
kol3_P6.ab1 TTGATCAGCCTCATCGATTTTATGTAGCTGATGTGTACACTGATCTTACCCCACTCAGTAAATTTCCTTCCCCTGAGTATGAAACTTTTGCAGAATATTA
kol3_P8.ab1 --
kol3_P10.ab1 --
kol3_P12.ab1 --
3_P13.ab1 --

```
ref/sequencing hDcr TAAAACAAAGTACAACCTTGACCTAACCAATCTCAACCAGCCACTGCTGGATGTGGACCACACATCTTCAAGACTTAATCTTTTGACACCTCGACATTTG
                     K  T  K  Y  N  L  D  L  T  N  L  N  Q  P  L  L  D  V  D  H  T  S  S  R  L  N  L  L  T  P  R  H  L

3_Seq1.ab1          ----------------------------------------------------------------------------------------------------
kol3_1.ab1          ----------------------------------------------------------------------------------------------------
kol3_P3.ab1         ----------------------------------------------------------------------------------------------------
kol3_P4.ab1         ----------------------------------------------------------------------------------------------------
kol3_P6.ab1         TAAAACAAAGTACAACCTTGACCTAACCAATCTCAACCAGCCACTGCTGGATGTGGACCACACATCTTCAAGACTTAATCTTTTGACACCTCGACATTTG
kol3_P8.ab1         ----------------------------------------------------------------------------------------------------
kol3_P10.ab1        ----------------------------------------------------------------------------------------------------
kol3_P12.ab1        ----------------------------------------------------------------------------------------------------
3_P13.ab1           ----------------------------------------------------------------------------------------------------
```

```
                                     Eco47III                                                 PstI
ref/sequencing hDcr AATCAGAAGGGGAAAGCGCTTCCTTTAAGCAGTGCTGAGAAGAGGAAAGCCAAATGGGAAAGTCTGCAGAATAAACAGATACTGGTTCCAGAACTCTGTG
                    N  Q  K  G  K  A  L  P  L  S  S  A  E  K  R  K  A  K  W  E  S  L  Q  N  K  Q  I  L  V  P  E  L  C

3_Seq1.ab1          ----------------------------------------------------------------------------------------------------
kol3_1.ab1          ----------------------------------------------------------------------------------------------------
kol3_P3.ab1         ----------------------------------------------------------------------------------------------------
kol3_P4.ab1         ----------------------------------------------------------------------------------------------------
kol3_P6.ab1         AATCAGAAGGGGAAAGCGCTTCCTTTAAGCAGTGCTGAGAAGAGGAAAGCCAAATGGGAAAGTCTGCAGAATAAACAGATACTGGTTCCAGAACTCTGTG
kol3_P8.ab1         ----------------------------------------------------------------------------------------------------
kol3_P10.ab1        ----------------------------------------------------------------------------------------------------
kol3_P12.ab1        ----------------------------------------------------------------------------------------------------
3_P13.ab1           ----------------------------------------------------------------------------------------------------
```

```
                                                                                                PstI
ref/sequencing hDcr CTATACATCCAATTCCAGCATCACTGTGGAGAAAAGCTGTTTGTCTCCCCAGCATACTTTATCGCCTTCACTGCCTTTTGACTGCAGAGGAGCTAAGAGC
                    A  I  H  P  I  P  A  S  L  W  R  K  A  V  C  L  P  S  I  L  Y  R  L  H  C  L  L  T  A  E  E  L  R  A

3_Seq1.ab1          ----------------------------------------------------------------------------------------------------
kol3_1.ab1          ----------------------------------------------------------------------------------------------------
kol3_P3.ab1         ----------------------------------------------------------------------------------------------------
kol3_P4.ab1         ----------------------------------------------------------------------------------------------------
kol3_P6.ab1         CTATACATCCAATTCCAGCATCACTGTGGAGAAAAGCTGTTTGTCTCCCCAGCATACTTTATCGCCTTCACTGCCTTTTGACTGCAGAGGAGCTAAGAGC
kol3_P8.ab1         ----------------------------------------------------------------------------------------------------
kol3_P10.ab1        ----------------------------------------------------------------------------------------------------
kol3_P12.ab1        ----------------------------------------------------------------------------------------------------
3_P13.ab1           ----------------------------------------------------------------------------------------------------
```

```
ref/sequencing hDcr CCAGACTGCCAGCGATGCTGGCGTGGGAGTCAGATCACTTCCTGCGGATTTTAGATACCCTAACTTAGACTTCGGGTGGAAAAAATCTATTGACAGCAAA
                     Q  T  A  S  D  A  G  V  G  V  R  S  L  P  A  D  F  R  Y  P  N  L  D  F  G  W  K  K  S  I  D  S  K

3_Seq1.ab1          ----------------------------------------------------------------------------------------------------
kol3_1.ab1          ----------------------------------------------------------------------------------------------------
kol3_P3.ab1         ----------------------------------------------------------------------------------------------------
kol3_P4.ab1         ----------------------------------------------------------------------------------------------------
kol3_P6.ab1         CCAGACTGCCAGCGATGCTGGCGTGGGAGTCAGATCACTTCCTGCGGATTTTAGATACCCTAACTTAGACTTCGGGTGGAAAAAATCTATTGACAGCAAA
kol3_P8.ab1         ----------------------------------------------------------------------------------------------------
kol3_P10.ab1        ----------------------------------------------------------------------------------------------------
kol3_P12.ab1        ----------------------------------------------------------------------------------------------------
3_P13.ab1           ----------------------------------------------------------------------------------------------------
```

		PvuII (at CAGCTG); MunI (at CAATTG)
ref/sequencing hDcr		TCTTTCATCTCAATTTCTAACTCCTCTTCAGCTGAAAATGATAATTACTGTAAGCACAGCACAATTGTCCCTGAAAATGCTGCACATCAAGGTGCTAATA
		S F I S I S N S S S A E N D N Y C K H S T I V P E N A A H Q G A N
3_Seq1.ab1		--
kol3_1.ab1		--
kol3_P3.ab1		--
kol3_P4.ab1		--
kol3_P6.ab1		TCTTTCATCTCAATTTCTAACTCCTCTTCAGCTGAAAATGATAATTACTGTAAGCACAGCACAATTGTCCCTGAAAATGCTGCACATCAAGGTGCTAATA
kol3_P8.ab1		TCTTTCATCTCAATTTCTAACTCCTCTTCAGCTGAAAATGATAATTACTGTAAGCACAGCACAATTGTCCCTGAAAATGCTGCACATCAAGGTGCTAATA
kol3_P10.ab1		--
kol3_P12.ab1		--
3_P13.ab1		--

		XbaI (at TCTAGA); PstI (at CTGCAG); AclI (at AACGTT); BglII (at AGATCT)
ref/sequencing hDcr		GAACCTCCTCTCTAGAAAATCATGACCAAATGTCTGTGAACTGCAGAACGTTGCTCAGCGAGTCCCCTGGTAAGCTCCACGTTGAAGTTTCAGCAGATCT
		R T S S L E N H D Q M S V N C R T L L S E S P G K L H V E V S A D L
3_Seq1.ab1		--
kol3_1.ab1		--
kol3_P3.ab1		--
kol3_P4.ab1		--
kol3_P6.ab1		GAACCTCCTCTCTAGAAAATCATGACCAAATGTCTGTGAACTGCAGAACGTTGCTCAGCGAGTCCCCTGGTAAGCTCCACGTTGAAGTTTCAGCAGATCT
kol3_P8.ab1		GAACCTCCTCTCTAGAAAATCATGACCAAATGTCTGTGAACTGCAGAACGTTGCTCAGCGAGTCCCCTGGTAAGCTCCACGTTGAAGTTTCAGCAGATCT
kol3_P10.ab1		--
kol3_P12.ab1		--
3_P13.ab1		--

VspI

ref/sequencing hDcr	TACAGCAATTAATGGTCTTTCTTACAATCAAAATCTCGCCAATGGCAGTTATGATTTAGCTAACAGAGACTTTTGCCAAGGAAATCAGCTAAATTACTAC
	T A I N G L S Y N Q N L A N G S Y D L A N R D F C Q G N Q L N Y Y
3_Seq1.ab1	-
kol3_1.ab1	-
kol3_P3.ab1	-
kol3_P4.ab1	-
kol3_P6.ab1	TACAGCAATTAATGGTCTTTCTTACAATCAAAATCTCGCCAATGGCAGTTATGATTTAGCTAACAGAGACTTTTGCCAAGGAAATCAGCTAAATTACTAC
kol3_P8.ab1	TACAGCAATTAATGGTCTTTCTTACAATCAAAATCTCGCCAATGGCAGTTATGATTTAGCTAACAGAGACTTTTGCCAAGGAAATCAGCTAAATTACTAC
kol3_P10.ab1	-
kol3_P12.ab1	-
3_P13.ab1	-

ref/sequencing hDcr	AAGCAGGAAATACCCGTGCAACCAACTACCTCATATTCCATTCAGAATTTATACAGTTACGAGAACCAGCCCCAGCCCAGCGATGAATGTACTCTCCTGA
	K Q E I P V Q P T T S Y S I Q N L Y S Y E N Q P Q P S D E C T L L
3_Seq1.ab1	-
kol3_1.ab1	-
kol3_P3.ab1	-
kol3_P4.ab1	-
kol3_P6.ab1	AAGCAGGAAATACCCGTGCAACCAACTACCTCATATTCCATTCAGAATTTATACAGTTACGAGAACCAGCCCCAGCCCAGCGATGAATGTACTCTCCTGA
kol3_P8.ab1	AAGCAGGAAATACCCGTGCAACCAACTACCTCATATTCCATTCAGAATTTATACAGTTACGAGAACCAGCCCCAGCCCAGCGATGAATGTACTCTCCTGA
kol3_P10.ab1	-
kol3_P12.ab1	-
3_P13.ab1	-

```
ref/sequencing hDcr GTAATAAATACCTTGATGGAAATGCTAACAAATCTACCTCAGATGGAAGTCCTGTGATGGCCGTAATGCCTGGTACGACAGACACTATTCAAGTGCTCAA
                    S  N  K  Y  L  D  G  N  A  N  K  S  T  S  D  G  S  P  V  M  A  V  M  P  G  T  T  D  T  I  Q  V  L  K

3_Seq1.ab1          ------------------------------------------------------------------------------------------------------
kol3_1.ab1          ------------------------------------------------------------------------------------------------------
kol3_P3.ab1         ------------------------------------------------------------------------------------------------------
kol3_P4.ab1         ------------------------------------------------------------------------------------------------------
kol3_P6.ab1         GTAATAAATACCTT----------------------------------------------------------------------------------------
kol3_P8.ab1         GTAATAAATACCTTGATGGAAATGCTAACAAATCTACCTCAGATGGAAGTCCTGTGATGGCCGTAATGCCTGGTACGACAGACACTATTCAAGTGCTCAA
kol3_P10.ab1        ------------------------------------------------------------------------------------------------------
kol3_P12.ab1        ------------------------------------------------------------------------------------------------------
3_P13.ab1           ------------------------------------------------------------------------------------------------------
```

```
                                                                                                     Tth111I
ref/sequencing hDcr GGGCAGGATGGATTCTGAGCAGAGCCCTTCTATTGGGTACTCCTCAAGGACTCTTGGCCCCAATCCTGGACTTATTCTTCAGGCTTTGACTCTGTCAAAC
                      G  R  M  D  S  E  Q  S  P  S  I  G  Y  S  S  R  T  L  G  P  N  P  G  L  I  L  Q  A  L  T  L  S  N

3_Seq1.ab1          ------------------------------------------------------------------------------------------------------
kol3_1.ab1          ------------------------------------------------------------------------------------------------------
kol3_P3.ab1         ------------------------------------------------------------------------------------------------------
kol3_P4.ab1         ------------------------------------------------------------------------------------------------------
kol3_P6.ab1         ------------------------------------------------------------------------------------------------------
kol3_P8.ab1         GGGCAGGATGGATTCTGAGCAGAGCCCTTCTATTGGGTACTCCTCAAGGACTCTTGGCCCCAATCCTGGACTTATTCTTCAGGCTTTGACTCTGTCAAAC
kol3_P10.ab1        ------------------------------------------------------------------------------------------------------
kol3_P12.ab1        ------------------------------------------------------------------------------------------------------
3_P13.ab1           ------------------------------------------------------------------------------------------------------
```

	DraI SphI
ref/sequencing hDcr	GCTAGTGATGGATTTAACCTGGAGCGGCTTGAAATGCTTGGCGACTCCTTTTTAAAGCATGCCATCACCACATATCTATTTTGCACTTACCCTGATGCGC
	A S D G F N L E R L E M L G D S F L K H A I T T Y L F C T Y P D A
3_Seq1.ab1	--
kol3_1.ab1	--
kol3_P3.ab1	--
kol3_P4.ab1	--
kol3_P6.ab1	--
kol3_P8.ab1	GCTAGTGATGGATTTAACCTGGAGCGGCTTGAAATGCTTGGCGACTCCTTTTTAAAGCATGCCATCACCACATATCTATTTTGCACTTACCCTGATGCGC
kol3_P10.ab1	--
kol3_P12.ab1	--
3_P13.ab1	--

	BstXI
ref/sequencing hDcr	ATGAGGGCCGCCTTTCATATATGAGAAGCAAAAAGGTCAGCAACTGTAATCTGTATCGCCTTGGAAAAAAGAAGGGACTACCCAGCCGCATGGTGGTGTC
	H E G R L S Y M R S K K V S N C N L Y R L G K K K G L P S R M V V S
3_Seq1.ab1	--
kol3_1.ab1	--
kol3_P3.ab1	--
kol3_P4.ab1	--
kol3_P6.ab1	--
kol3_P8.ab1	ATGAGGGCCGCCTTTCATATATGAGAAGCAAAAAGGTCAGCAACTGTAATCTGTATCGCCTTGGAAAAAAGAAGGGACTACCCAGCCGCATGGTGGTGTC
kol3_P10.ab1	--
kol3_P12.ab1	--
3_P13.ab1	--

```
                     SspI
ref/sequencing hDcr  AATATTTGATCCCCCTGTGAATTGGCTTCCTCCTGGTTATGTAGTAAATCAAGACAAAAGCAACACAGATAAATGGGAAAAAGATGAAATGACAAAAGAC
                      I  F  D  P  P  V  N  W  L  P  P  G  Y  V  V  N  Q  D  K  S  N  T  D  K  W  E  K  D  E  M  T  K  D

3_Seq1.ab1           ----------------------------------------------------------------------------------------------------
kol3_1.ab1           ----------------------------------------------------------------------------------------------------
kol3_P3.ab1          ----------------------------------------------------------------------------------------------------
kol3_P4.ab1          ----------------------------------------------------------------------------------------------------
kol3_P6.ab1          ----------------------------------------------------------------------------------------------------
kol3_P8.ab1          AATATTTGATCCCCCTGTGAATTGGCTTCCTCCTGGTTATGTAGTAAATCAAGACAAAAGCAACACAGATAAATGGGAAAAAGATGAAATGACAAAAGAC
kol3_P10.ab1         ----------------------------------------------------------------------------------------------------
kol3_P12.ab1         ----------------------------------------------------------------------------------------------------
3_P13.ab1            ----------------------------------------------------------------------------------------------------
```

```
                           SphI
ref/sequencing hDcr  TGCATGCTGGCGAATGGCAAACTGGATGAGGATTACGAGGAGGAGGATGAGGAGGAGGAGAGCCTGATGTGGAGGGCTCCGAAGGAAGAGGCTGACTATG
                      C  M  L  A  N  G  K  L  D  E  D  Y  E  E  E  D  E  E  E  E  S  L  M  W  R  A  P  K  E  E  A  D  Y

3_Seq1.ab1           ----------------------------------------------------------------------------------------------------
kol3_1.ab1           ----------------------------------------------------------------------------------------------------
kol3_P3.ab1          ----------------------------------------------------------------------------------------------------
kol3_P4.ab1          ----------------------------------------------------------------------------------------------------
kol3_P6.ab1          ----------------------------------------------------------------------------------------------------
kol3_P8.ab1          TGCATGCTGGCGAATGGCAAACTGGATGAGGATTACGAGGAGGAGGATGAGGAGGAGGAGAGCCTGATGTGGAGGGCTCCGAAGGAAGAGGCTGACTATG
kol3_P10.ab1         ---------------------CTGGATGAGGATTACGAGGAGGAGGATGAGGAGGAGGAGAGCCTGATGTGGAGGGCTCCGAAGGAAGAGGCTGACTATG
kol3_P12.ab1         ----------------------------------------------------------------------------------------------------
3_P13.ab1            ----------------------------------------------------------------------------------------------------
```

```
                                         BclI*
ref/sequencing hDcr  AAGATGATTTCCTGGAGTATGATCAGGAACATATCAGATTTATAGATAATATGTTAATGGGGTCAGGAGCTTTTGTAAAGAAAATCTCTCTTTCTCCTTT
                     E  D  D  F  L  E  Y  D  Q  E  H  I  R  F  I  D  N  M  L  M  G  S  G  A  F  V  K  K  I  S  L  S  P  F

3_Seq1.ab1           ----------------------------------------------------------------------------------------------------
kol3_1.ab1           ----------------------------------------------------------------------------------------------------
kol3_P3.ab1          ----------------------------------------------------------------------------------------------------
kol3_P4.ab1          ----------------------------------------------------------------------------------------------------
kol3_P6.ab1          ----------------------------------------------------------------------------------------------------
kol3_P8.ab1          AAGATGATTTCCTGGAGTATGATCAGGAACATATCAGATTTATAGATAATATGTTAATGGGGTCAGGAGCTTTTGTAAAGAAAATCTCTCTTTCTCCTTT
kol3_P10.ab1         AAGATGATTTCCTGGAGTATGATCAGGAACATATCAGATTTATAGATAATATGTTAATGGGGTCAGGAGCTTTTGTAAAGAAAATCTCTCTTTCTCCTTT
kol3_P12.ab1         ----------------------------------------------------------------------------------------------------
3_P13.ab1            ----------------------------------------------------------------------------------------------------
```

```
                                        NdeI
ref/sequencing hDcr  TTCAACCACTGATTCTGCATATGAATGGAAAATGCCCAAAAAATCCTCCTTAGGTAGTATGCCATTTTCATCAGATTTTGAGGATTTTGACTACAGCTCT
                       S  T  T  D  S  A  Y  E  W  K  M  P  K  K  S  S  L  G  S  M  P  F  S  S  D  F  E  D  F  D  Y  S  S

3_Seq1.ab1           ----------------------------------------------------------------------------------------------------
kol3_1.ab1           ----------------------------------------------------------------------------------------------------
kol3_P3.ab1          ----------------------------------------------------------------------------------------------------
kol3_P4.ab1          ----------------------------------------------------------------------------------------------------
kol3_P6.ab1          ----------------------------------------------------------------------------------------------------
kol3_P8.ab1          TTCAACCACTGATTCTGCATATGAATGGAAAATGCCCAAAAAATCCTCCTTAGGTAGTATGCCATTTTCATCAGATTTTGAGGATTTTGACTACAGCTCT
kol3_P10.ab1         TTCAACCACTGATTCTGCATATGAATGGAAAATGCCCAAAAAATCCTCCTTAGGTAGTATGCCATTTTCATCAGATTTTGAGGATTTTGACTACAGCTCT
kol3_P12.ab1         ----------------------------------------------------------------------------------------------------
3_P13.ab1            ----------------------------------------------------------------------------------------------------
```

```
                      BamHI
ref/sequencing hDcr TGGGATGCAATGTGCTATCTGGATCCTAGCAAAGCTGTTGAAGAAGATGACTTTGTGGTGGGGTTCTGGAATCCATCAGAAGAAAACTGTGGTGTTGACA
                     W  D  A  M  C  Y  L  D  P  S  K  A  V  E  E  D  D  F  V  V  G  F  W  N  P  S  E  E  N  C  G  V  D

3_Seq1.ab1          ---------------------------------------------------------------------------------------------------
kol3_1.ab1          ---------------------------------------------------------------------------------------------------
kol3_P3.ab1         ---------------------------------------------------------------------------------------------------
kol3_P4.ab1         ---------------------------------------------------------------------------------------------------
kol3_P6.ab1         ---------------------------------------------------------------------------------------------------
kol3_P8.ab1         TGGGATGCAATGTGCTATCTGGATCCTAGCAAAGCTGTTGAAGAAGATGACTTTGTGGTGGGGTTCTGGAATCCATCAGAAGAAAACT------------
kol3_P10.ab1        TGGGATGCAATGTGCTATCTGGATCCTAGCAAAGCTGTTGAAGAAGATGACTTTGTGGTGGGGTTCTGGAATCCATCAGAAGAAAACTGTGGTGTTGACA
kol3_P12.ab1        ---------------------------------------------------------------------------------------------------
3_P13.ab1           ---------------------------------------------------------------------------------------------------
```

```
ref/sequencing hDcr CGGGAAAGCAGTCCATTTCTTACGACTTGCACACTGAGCAGTGTATTGCTGACAAAAGCATAGCGGACTGTGTGGAAGCCCTGCTGGGCTGCTATTTAAC
                    T  G  K  Q  S  I  S  Y  D  L  H  T  E  Q  C  I  A  D  K  S  I  A  D  C  V  E  A  L  L  G  C  Y  L  T

3_Seq1.ab1          ---------------------------------------------------------------------------------------------------
kol3_1.ab1          ---------------------------------------------------------------------------------------------------
kol3_P3.ab1         ---------------------------------------------------------------------------------------------------
kol3_P4.ab1         ---------------------------------------------------------------------------------------------------
kol3_P6.ab1         ---------------------------------------------------------------------------------------------------
kol3_P8.ab1         ---------------------------------------------------------------------------------------------------
kol3_P10.ab1        CGGGAAAGCAGTCCATTTCTTACGACTTGCACACTGAGCAGTGTATTGCTGACAAAAGCATAGCGGACTGTGTGGAAGCCCTGCTGGGCTGCTATTTAAC
kol3_P12.ab1        ---------------------------------------------------------------------------------------------------
3_P13.ab1           ---------------------------------------------------------------------------------------------------
```

PvuII

ref/sequencing hDcr	CAGCTGTGGGGAGAGGGCTGCTCAGCTTTTCCTCTGTTCACTGGGGCTGAAGGTGCTCCCGGTAATTAAAAGGACTGATCGGGAAAAGGCCCTGTGCCCT
	S C G E R A A Q L F L C S L G L K V L P V I K R T D R E K A L C P
3_Seq1.ab1	--
kol3_1.ab1	--
kol3_P3.ab1	--
kol3_P4.ab1	--
kol3_P6.ab1	--
kol3_P8.ab1	--
kol3_P10.ab1	CAGCTGTGGGGAGAGGGCTGCTCAGCTTTTCCTCTGTTCACTGGGGCTGAAGGTGCTCCCGGTAATTAAAAGGACTGATCGGGAAAAGGCCCTGTGCCCT
kol3_P12.ab1	--
3_P13.ab1	--

BalI

ref/sequencing hDcr	ACTCGGGAGAATTTCAACAGCCAACAAAAGAACCTTTCAGTGAGCTGTGCTGCTGCTTCTGTGGCCAGTTCACGCTCTTCTGTATTGAAAGACTCGGAAT
	T R E N F N S Q Q K N L S V S C A A A S V A S S R S S V L K D S E
3_Seq1.ab1	--
kol3_1.ab1	--
kol3_P3.ab1	--
kol3_P4.ab1	--
kol3_P6.ab1	--
kol3_P8.ab1	--
kol3_P10.ab1	ACTCGGGAGAATTTCAACAGCCAACAAAAGAACCTTTCAGTGAGCTGTGCTGCTGCTTCTGTGGCCAGTTCACGCTCTTCTGTATTGAAAGACTCGGAAT
kol3_P12.ab1	--
3_P13.ab1	--

```
                                           BclI*
ref/sequencing hDcr ATGGTTGTTTGAAGATTCCACCAAGATGTATGTTTGATCATCCAGATGCAGATAAAACACTGAATCACCTTATATCGGGGTTTGAAAATTTTGAAAAGAA
                    Y  G  C  L  K  I  P  P  R  C  M  F  D  H  P  D  A  D  K  T  L  N  H  L  I  S  G  F  E  N  F  E  K  K

3_Seq1.ab1          ----------------------------------------------------------------------------------------------------
kol3_1.ab1          ----------------------------------------------------------------------------------------------------
kol3_P3.ab1         ----------------------------------------------------------------------------------------------------
kol3_P4.ab1         ----------------------------------------------------------------------------------------------------
kol3_P6.ab1         ----------------------------------------------------------------------------------------------------
kol3_P8.ab1         ----------------------------------------------------------------------------------------------------
kol3_P10.ab1        ATGGTTGTTTGAAGATTCCACCAAGATGTATGTTTGATCATCCAGATGCAGATAAAACACTGAATCACCTTATATCGGGGTTTGAAAATTTTGAAAAGAA
kol3_P12.ab1        ----------------------------------------------------------------------------------------------------
3_P13.ab1           ----------------------------------------------------------------------------------------------------
```

```
                                                                                           Eco47III Ec
ref/sequencing hDcr AATCAACTACAGATTCAAGAATAAGGCTTACCTTCTCCAGGCTTTTACACATGCCTCCTACCACTACAATACTATCACTGATTGTTACCAGCGCTTAGAA
                     I  N  Y  R  F  K  N  K  A  Y  L  L  Q  A  F  T  H  A  S  Y  H  Y  N  T  I  T  D  C  Y  Q  R  L  E

3_Seq1.ab1          ----------------------------------------------------------------------------------------------------
kol3_1.ab1          ----------------------------------------------------------------------------------------------------
kol3_P3.ab1         ----------------------------------------------------------------------------------------------------
kol3_P4.ab1         ----------------------------------------------------------------------------------------------------
kol3_P6.ab1         ----------------------------------------------------------------------------------------------------
kol3_P8.ab1         ----------------------------------------------------------------------------------------------------
kol3_P10.ab1        AATCAACTACAGATTCAAGAATAAGGCTTACCTTCTCCAGGCTTTTACACATGCCTCCTACCACTACAATACTATCACTGATTGTTACCAGCGCTTAGAA
kol3_P12.ab1        ----------------------------------------------------------------------------------------------------
3_P13.ab1           ----------------------------------------------------------------------------------------------------
```

```
                      oRI                                                                                       |SacII          |XmaI |SmaI
ref/sequencing hDcr   TTCCTGGGAGATGCGATTTTGGACTACCTCATAACCAAGCACCTTTATGAAGACCCGCGGCAGCACTCCCCGGGGGTCCTGACAGACCTGCGGTCTGCCC
                       F  L  G  D  A  I  L  D  Y  L  I  T  K  H  L  Y  E  D  P  R  Q  H  S  P  G  V  L  T  D  L  R  S  A

3_Seq1.abl            ----------------------------------------------------------------------------------------------------
kol3_1.abl            ----------------------------------------------------------------------------------------------------
kol3_P3.abl           ----------------------------------------------------------------------------------------------------
kol3_P4.abl           ----------------------------------------------------------------------------------------------------
kol3_P6.abl           ----------------------------------------------------------------------------------------------------
kol3_P8.abl           ----------------------------------------------------------------------------------------------------
kol3_P10.abl          TTCCTGGGAGATGCGATTTTGGACTACCTCATAACCAAGCACCTTTATGAAGACCCGCGGCAGCACTCCCCGGGGGTCCTGACAGACCTGCGGTCTGCCC
kol3_P12.abl          --------------------------------------------------------GCGGCAGCACTCCCCGGGGGTCCTGACAGACCTGCGGTCTGCCC
3_P13.abl             ----------------------------------------------------------------------------------------------------
```

```
                                                                                     |ScaI                    |Bpu10I |EcoICRI |SacI
ref/sequencing hDcr   TGGTCAACAACACCATCTTTGCATCGCTGGCTGTAAAGTACGACTACCACAAGTACTTCAAAGCTGTCTCTCCTGAGCTCTTCCATGTCATTGATGACTT
                      L  V  N  N  T  I  F  A  S  L  A  V  K  Y  D  Y  H  K  Y  F  K  A  V  S  P  E  L  F  H  V  I  D  D  F

3_Seq1.abl            ----------------------------------------------------------------------------------------------------
kol3_1.abl            ----------------------------------------------------------------------------------------------------
kol3_P3.abl           ----------------------------------------------------------------------------------------------------
kol3_P4.abl           ----------------------------------------------------------------------------------------------------
kol3_P6.abl           ----------------------------------------------------------------------------------------------------
kol3_P8.abl           ----------------------------------------------------------------------------------------------------
kol3_P10.abl          TGGTCAACAACACCATCTTTGCATCGCTGGCTGTAAAGTACGACTACCACAAGTACTTCAAAGCTGTCTCTCCTGAGCTCTTCCATGTCATTGATGACTT
kol3_P12.abl          TGGTCAACAACACCATCTTTGCATCGCTGGCTGTAAAGTACGACTACCACAAGTACTTCAAAGCTGTCTCTCCTGAGCTCTTCCATGTCATTGATGACTT
3_P13.abl             ----------------------------------------------------------------------------------------------------
```

```
                            Bpu10I BglII
ref/sequencing hDcr  TGTGCAGTTTCAGCTTGAGAAGAATGAAATGCAAGGAATGGATTCTGAGCTTAGGAGATCTGAGGAGGATGAAGAGAAAGAAGAGGATATTGAAGTTCCA
                      V  Q  F  Q  L  E  K  N  E  M  Q  G  M  D  S  E  L  R  R  S  E  E  D  E  E  K  E  E  D  I  E  V  P

3_Seq1.ab1           ----------------------------------------------------------------------------------------------------
kol3_1.ab1           ----------------------------------------------------------------------------------------------------
kol3_P3.ab1          ----------------------------------------------------------------------------------------------------
kol3_P4.ab1          ----------------------------------------------------------------------------------------------------
kol3_P6.ab1          ----------------------------------------------------------------------------------------------------
kol3_P8.ab1          ----------------------------------------------------------------------------------------------------
kol3_P10.ab1         TGTGCAGTTTCAGCTTGAGAAGAATGAAATGCAAGGAATGGATTCTGAGCTTAGGAGATCTGAGGAGGATGAAGAGAAAGAAGAGGATATTGAAGTTCCA
kol3_P12.ab1         TGTGCAGTTTCAGCTTGAGAAGAATGAAATGCAAGGAATGGATTCTGAGCTTAGGAGATCTGAGGAGGATGAAGAGAAAGAAGAGGATATTGAAGTTCCA
3_P13.ab1            ----------------------------------------------------------------------------------------------------
```

```
                        NcoI                                        BstXI
ref/sequencing hDcr  AAGGCCATGGGGGATATTTTTGAGTCGCTTGCTGGTGCCATTTACATGGATAGTGGGATGTCACTGGAGACAGTCTGGCAGGTGTACTATCCCATGATGC
                      K  A  M  G  D  I  F  E  S  L  A  G  A  I  Y  M  D  S  G  M  S  L  E  T  V  W  Q  V  Y  Y  P  M  M

3_Seq1.ab1           ----------------------------------------------------------------------------------------------------
kol3_1.ab1           ----------------------------------------------------------------------------------------------------
kol3_P3.ab1          ----------------------------------------------------------------------------------------------------
kol3_P4.ab1          ----------------------------------------------------------------------------------------------------
kol3_P6.ab1          ----------------------------------------------------------------------------------------------------
kol3_P8.ab1          ----------------------------------------------------------------------------------------------------
kol3_P10.ab1         AAGGCCATGGGGGATATTTTTGAGTCGCTTGCTGGTGCCATTTACATGGATAGTGGGATGTCACTGGA--------------------------------
kol3_P12.ab1         AAGGCCATGGGGGATATTTTTGAGTCGCTTGCTGGTGCCATTTACATGGATAGTGGGATGTCACTGGAGACAGTCTGGCAGGTGTACTATCCCATGATGC
3_P13.ab1            ----------------------------------------------------------------------------------------------------
```

```
ref/sequencing hDcr GGCCACTAATAGAAAAGTTTTCTGCAAATGTACCCCGTTCCCCTGTGCGAGAATTGCTTGAAATGGAACCAGAAACTGCCAAATTTAGCCCGGCTGAGAG
                    R  P  L  I  E  K  F  S  A  N  V  P  R  S  P  V  R  E  L  L  E  M  E  P  E  T  A  K  F  S  P  A  E  R

3_Seq1.ab1          ----------------------------------------------------------------------------------------------------
kol3_1.ab1          ----------------------------------------------------------------------------------------------------
kol3_P3.ab1         ----------------------------------------------------------------------------------------------------
kol3_P4.ab1         ----------------------------------------------------------------------------------------------------
kol3_P6.ab1         ----------------------------------------------------------------------------------------------------
kol3_P8.ab1         ----------------------------------------------------------------------------------------------------
kol3_P10.ab1        ----------------------------------------------------------------------------------------------------
kol3_P12.ab1        GGCCACTAATAGAAAAGTTTTCTGCAAATGTACCCCGTTCCCCTGTGCGAGAATTGCTTGAAATGGAACCAGAAACTGCCAAATTTAGCCCGGCTGAGAG
3_P13.ab1           ----------------------------TGTACCCCGTTCCCCTGTGCGAGAATTGCTTGAAATGGAACCAGAAACTGCCAAATTTAGCCCGGCTGAGAG
```

```
                                                                       DraI                                   PstI
ref/sequencing hDcr AACTTACGACGGGAAGGTCAGAGTCACTGTGGAAGTAGTAGGAAAGGGGAAATTTAAAGGTGTTGGTCGAAGTTACAGGATTGCCAAATCTGCAGCAGCA
                      T  Y  D  G  K  V  R  V  T  V  E  V  V  G  K  G  K  F  K  G  V  G  R  S  Y  R  I  A  K  S  A  A  A

3_Seq1.ab1          ----------------------------------------------------------------------------------------------------
kol3_1.ab1          ----------------------------------------------------------------------------------------------------
kol3_P3.ab1         ----------------------------------------------------------------------------------------------------
kol3_P4.ab1         ----------------------------------------------------------------------------------------------------
kol3_P6.ab1         ----------------------------------------------------------------------------------------------------
kol3_P8.ab1         ----------------------------------------------------------------------------------------------------
kol3_P10.ab1        ----------------------------------------------------------------------------------------------------
kol3_P12.ab1        AACTTACGACGGGAAGGTCAGAGTCACTGTGGAAGTAGTAGGAAAGGGGAAATTTAAAGGTGTTGGTCGAAGTTACAGGATTGCCAAATCTGCAGCAGCA
3_P13.ab1           AACTTACGACGGGAAGGTCAGAGTCACTGTGGAAGTAGTAGGAAAGGGGAAATTTAAAGGTGTTGGTCGAAGTTACAGGATTGCCAAATCTGCAGCAGCA
```

```
ref/sequencing hDcr AGAAGAGCCCTCCGAAGCCTCAAAGCTAATCAACCTCAGGTTCCCAATAGCGGTGGCGGAGGTTCTGGAGGCGGTGGAAGTGACTACAAGGACGACGATG
                    R  R  A  L  R  S  L  K  A  N  Q  P  Q  V  P  N  S  G  G  G  G  S  G  G  G  G  S  D  Y  K  D  D  D

3_Seq1.abl         ----------------------------------------------------------------------------------------------------
kol3_1.abl         ----------------------------------------------------------------------------------------------------
kol3_P3.abl        ----------------------------------------------------------------------------------------------------
kol3_P4.abl        ----------------------------------------------------------------------------------------------------
kol3_P6.abl        ----------------------------------------------------------------------------------------------------
kol3_P8.abl        ----------------------------------------------------------------------------------------------------
kol3_P10.abl       ----------------------------------------------------------------------------------------------------
kol3_P12.abl       AGAAGAGCCCTCCGAAGCCTCAAAGCTAATCAACCTCAGGTTCCCAATAGCGGTGGCGGAGGTTCTGGAGGCGGTGGAAGTGACTACAAGGACGACGATG
3_P13.abl          AGAAGAGCCCTCCGAAGCCTCAAAGCTAATCAACCTCAGGTTCCCAATAGCGGTGGCGGAGGTTCTGGAGGCGGTGGAAGTGACTACAAGGACGACGATG
```

```
                                                                                                 | XhoI
ref/sequencing hDcr ACAAGGATTACAAAGACGATGATGACAAGGACTATAAGGACGATGACGATAAGTAACATTTGGTTTAGTGTACAATATCTCCTCGAGCATTTGGTTTAGT
                   D  K  D  Y  K  D  D  D  D  K  D  Y  K  D  D  D  D  K  *  H  L  V  *  C  T  I  S  P  R  A  F  G  L  V
                                                                          Stop

3_Seq1.abl         ----------------------------------------------------------------------------------------------------
kol3_1.abl         ----------------------------------------------------------------------------------------------------
kol3_P3.abl        ----------------------------------------------------------------------------------------------------
kol3_P4.abl        ----------------------------------------------------------------------------------------------------
kol3_P6.abl        ----------------------------------------------------------------------------------------------------
kol3_P8.abl        ----------------------------------------------------------------------------------------------------
kol3_P10.abl       ----------------------------------------------------------------------------------------------------
kol3_P12.abl       ACAAGGATTACAAAGACGATGATGACAAGGACTATAAGGACGATGACGATAAGTAACATTTGGTTTAGTGTACAATATCTCCTCGA--------------
3_P13.abl          ACAAGGATTACAAAGACGATGATGACAAGGACTATAAGGACGATGACGATAAGTAACATTTGGTTTAGTGTACAATATCTCCTCGAG-------------
```

XhoI

ref/sequencing hDcr GTACAATATCTCCTCGAGGGCTCGTGGCCTCGACTGTGCCTTCTAGTTGCCAGCCATCTGTTGTTTGC
Y N I S S R A R G L D C A F * L P A I C C L

3_Seq1.ab1 --
kol3_1.ab1 --
kol3_P3.ab1 --
kol3_P4.ab1 --
kol3_P6.ab1 --
kol3_P8.ab1 --
kol3_P10.ab1 --
kol3_P12.ab1 --
3_P13.ab1 --

ref/sequencing hDcr

3_Seq1.ab1
kol3_1.ab1
kol3_P3.ab1
kol3_P4.ab1
kol3_P6.ab1
kol3_P8.ab1
kol3_P10.ab1
kol3_P12.ab1
3_P13.ab1

```
                                        AatII
                                       ZraI
ref/seq delPAZ_hDcr CCAAGTCTCCACCCCATTGACGTCAATGGGAGTTTGTTTTGGCACCAAAATCAACGGGACTTTCCAAAATGTCGTAACAACTCCGCCCCATTGACGCAAA
                     P  S  L  H  P  I  D  V  N  G  S  L  F  W  H  Q  N  Q  R  D  F  P  K  C  R  N  N  S  A  P  L  T  Q

PAZ4_SeqP2.abi      CCAAGTCTCCACCCCATTGACGTCAATGGGAGTTTGTTTTGGCACCAAAATCAACGGGACTTTCCAAAATGTCGTAACAACTCCGCCCCATTGACGCAAA
PAZ4_1.abi          ----------------------------------------------------------------------------------------------------
PAZ4_P3.abi         ----------------------------------------------------------------------------------------------------
PAZ4_P4.abi         ----------------------------------------------------------------------------------------------------
PAZ4_P6.abi         ----------------------------------------------------------------------------------------------------
PAZ4_P8.abi         ----------------------------------------------------------------------------------------------------
PAZ4_P10.abi        ----------------------------------------------------------------------------------------------------
PAZ4_P12.abi        ----------------------------------------------------------------------------------------------------
```

```
                                                                                    BglII
ref/seq delPAZ_hDcr TGGGCGGTAGGCGTGTACGGTGGGAGGTCTATATAAGCAGAGCTGGTTTAGTGAACCGTCAGATCAGATCTTTGTCGATCCTACCATCCACTCGACACAC
                    M  G  G  R  R  V  R  W  E  V  Y  I  S  R  A  G  L  V  N  R  Q  I  R  S  L  S  I  L  P  S  T  R  H  T
                    Start

PAZ4_SeqP2.abi      TGGGCGGTAGGCGTGTACGGTGGGAGGTCTATATAAGCAGAGCTGGTTTAGTGAACCGTCAGATCAGATCTTTGTCGATCCTACCATCCACTCGACACAC
PAZ4_1.abi          ----------------------------------------------------------------------------------------------------
PAZ4_P3.abi         ----------------------------------------------------------------------------------------------------
PAZ4_P4.abi         ----------------------------------------------------------------------------------------------------
PAZ4_P6.abi         ----------------------------------------------------------------------------------------------------
PAZ4_P8.abi         ----------------------------------------------------------------------------------------------------
PAZ4_P10.abi        ----------------------------------------------------------------------------------------------------
PAZ4_P12.abi        ----------------------------------------------------------------------------------------------------
```

```
                                         |SacI                 |KpnI
                    |NotI         |HindIII    |EcoICRI|EcoRI      |Acc65I          |DraI
ref/seq delPAZ_hDcr CCGCCAGCGGCCGCTGCCAAGCTTCCGAGCTCTCGAATTCAAAGGAGGTACCCACCCTTGTTTAAACTTTAAGAGGAGGGCCACCATGAAAAGCCCTGCT
                     R  Q  R  P  L  P  S  F  R  A  L  E  F  K  G  G  T  H  P  C  L  N  F  K  R  R  A  T  M  K  S  P  A

PAZ4_SeqP2.abi      CCGCCAGCGGCCGCTGCCAAGCTTCCGAGCTCTCGAATTCAAAGGAGGTACCCACCCTTGTTTAAACTTTAAGAGGAGGGCCACCATGAAAAGCCCTGCT
PAZ4_1.abi          ----------------------------------------------------------------------------------------------------
PAZ4_P3.abi         ----------------------------------------------------------------------------------------------------
PAZ4_P4.abi         ----------------------------------------------------------------------------------------------------
PAZ4_P6.abi         ----------------------------------------------------------------------------------------------------
PAZ4_P8.abi         ----------------------------------------------------------------------------------------------------
PAZ4_P10.abi        ----------------------------------------------------------------------------------------------------
PAZ4_P12.abi        ----------------------------------------------------------------------------------------------------
```

```
                              |Bpu10I     |StuI  |PstI                                             |NcoI
ref/seq delPAZ_hDcr TTGCAACCCCTCAGCATGGCAGGCCTGCAGCTCATGACCCCTGCTTCCTCACCAATGGGTCCTTTCTTTGGACTGCCATGGCAACAAGAAGCAATTCATG
                     L  Q  P  L  S  M  A  G  L  Q  L  M  T  P  A  S  S  P  M  G  P  F  F  G  L  P  W  Q  Q  E  A  I  H

PAZ4_SeqP2.abi      TTGCAACCCCTCAGCATGGCAGGCCTGCAGCTCATGACCCCTGCTTCCTCACCAATGGGTCCTTTCTTTGGACTGCCATGGCAACAAGAAGCAATTCATG
PAZ4_1.abi          ----------------------------------TGACCCCTGCTTCCTCACCAATGGGTCCTTTCTTTGGACTGCCATGGCAACAAGAAGCAATTCATG
PAZ4_P3.abi         ----------------------------------------------------------------------------------------------------
PAZ4_P4.abi         ----------------------------------------------------------------------------------------------------
PAZ4_P6.abi         ----------------------------------------------------------------------------------------------------
PAZ4_P8.abi         ----------------------------------------------------------------------------------------------------
PAZ4_P10.abi        ----------------------------------------------------------------------------------------------------
PAZ4_P12.abi        ----------------------------------------------------------------------------------------------------
```

```
                                                                                                 DraI       Bpu10I
ref/seq delPAZ_hDcr ATAACATTTATACGCCAAGAAAATATCAGGTTGAACTGCTTGAAGCAGCTCTGGATCATAATACCATCGTCTGTTTAAACACTGGCTCAGGGAAGACATT
                    D  N  I  Y  T  P  R  K  Y  Q  V  E  L  L  E  A  A  L  D  H  N  T  I  V  C  L  N  T  G  S  G  K  T  F

PAZ4_SeqP2.abi      ATAACATTTATACGCCAAGAAAATATCAGGTTGAACTGCTTGAAGCAGCTCTGGATCATAATACCATCGTCTGTTTAAACACTGGCTCAGGGAAGACATT
PAZ4_1.abi          ATAACATTTATACGCCAAGAAAATATCAGGTTGAACTGCTTGAAGCAGCTCTGGATCATAATACCATCGTCTGTTTAAACACTGGCTCAGGGAAGACATT
PAZ4_P3.abi         ----------------------------------------------------------------------------------------------------
PAZ4_P4.abi         ----------------------------------------------------------------------------------------------------
PAZ4_P6.abi         ----------------------------------------------------------------------------------------------------
PAZ4_P8.abi         ----------------------------------------------------------------------------------------------------
PAZ4_P10.abi        ----------------------------------------------------------------------------------------------------
PAZ4_P12.abi        ----------------------------------------------------------------------------------------------------
```

```
                            ScaI
ref/seq delPAZ_hDcr TATTGCAGTACTACTCACTAAAGAGCTGTCCTATCAGATCAGGGGAGACTTCAGCAGAAATGGAAAAAGGACGGTGTTCTTGGTCAACTCTGCAAACCAG
                      I  A  V  L  L  T  K  E  L  S  Y  Q  I  R  G  D  F  S  R  N  G  K  R  T  V  F  L  V  N  S  A  N  Q

PAZ4_SeqP2.abi      TATTGCAGTACTACTCACTAAAGAGCTGTCCTATCAGATCAGGGGAGACTTCAGCAGAAATGGAAAAAGGACGGTGTTCTTGGTCAACTCTGCAAACCAG
PAZ4_1.abi          TATTGCAGTACTACTCACTAAAGAGCTGTCCTATCAGATCAGGGGAGACTTCAGCAGAAATGGAAAAAGGACGGTGTTCTTGGTCAACTCTGCAAACCAG
PAZ4_P3.abi         ----------------------------------------------------------------------------------------------------
PAZ4_P4.abi         ----------------------------------------------------------------------------------------------------
PAZ4_P6.abi         ----------------------------------------------------------------------------------------------------
PAZ4_P8.abi         ----------------------------------------------------------------------------------------------------
PAZ4_P10.abi        ----------------------------------------------------------------------------------------------------
PAZ4_P12.abi        ----------------------------------------------------------------------------------------------------
```

```
                          PvuII                  BglII                                        NsiI
ref/seq delPAZ_hDcr GTTGCTCAACAAGTGTCAGCTGTCAGAACTCATTCAGATCTCAAGGTTGGGGAATACTCAAACCTAGAAGTAAATGCATCTTGGACAAAAGAGAGATGGA
                     V  A  Q  Q  V  S  A  V  R  T  H  S  D  L  K  V  G  E  Y  S  N  L  E  V  N  A  S  W  T  K  E  R  W

PAZ4_SeqP2.abi      GTTGCTCAACAAGTGTCAGCTGTCAGAACTCATTCAGATCTCAAGGTTGGG------------------------------------------------
PAZ4_1.abi          GTTGCTCAACAAGTGTCAGCTGTCAGAACTCATTCAGATCTCAAGGTTGGGGAATACTCAAACCTAGAAGTAAATGCATCTTGGACAAAAGAGAGATGGA
PAZ4_P3.abi         ---------------------------------------------------------------------------------------------------
PAZ4_P4.abi         ---------------------------------------------------------------------------------------------------
PAZ4_P6.abi         ---------------------------------------------------------------------------------------------------
PAZ4_P8.abi         ---------------------------------------------------------------------------------------------------
PAZ4_P10.abi        ---------------------------------------------------------------------------------------------------
PAZ4_P12.abi        ---------------------------------------------------------------------------------------------------
```

```
ref/seq delPAZ_hDcr ACCAAGAGTTTACTAAGCACCAGGTTCTCATTATGACTTGCTATGTCGCCTTGAATGTTTTGAAAAATGGTTACTTATCACTGTCAGACATTAACCTTTT
                    N  Q  E  F  T  K  H  Q  V  L  I  M  T  C  Y  V  A  L  N  V  L  K  N  G  Y  L  S  L  S  D  I  N  L  L

PAZ4_SeqP2.abi      ---------------------------------------------------------------------------------------------------
PAZ4_1.abi          ACCAAGAGTTTACTAAGCACCAGGTTCTCATTATGACTTGCTATGTCGCCTTGAATGTTTTGAAAAATGGTTACTTATCACTGTCAGACATTAACCTTTT
PAZ4_P3.abi         ---------------------------------------------------------------------------------------------------
PAZ4_P4.abi         ---------------------------------------------------------------------------------------------------
PAZ4_P6.abi         ---------------------------------------------------------------------------------------------------
PAZ4_P8.abi         ---------------------------------------------------------------------------------------------------
PAZ4_P10.abi        ---------------------------------------------------------------------------------------------------
PAZ4_P12.abi        ---------------------------------------------------------------------------------------------------
```

```
ref/seq delPAZ_hDcr GGTGTTTGATGAGTGTCATCTTGCAATCCTAGACCACCCCTATCGAGAAATTATGAAGCTCTGTGAAAATTGTCCATCATGTCCTCGCATTTTGGGACTA
                      V  F  D  E  C  H  L  A  I  L  D  H  P  Y  R  E  I  M  K  L  C  E  N  C  P  S  C  P  R  I  L  G  L

PAZ4_SeqP2.abi      ----------------------------------------------------------------------------------------------------
PAZ4_1.abi          GGTGTTTGATGAGTGTCATCTTGCAATCCTAGACCACCCCTATCGAGAAATTATGAAGCTCTGTGAAAATTGTCCATCATGTCCTCGCATTTTGGGACTA
PAZ4_P3.abi         ----------------------------------------------------------------------------------------------------
PAZ4_P4.abi         ----------------------------------------------------------------------------------------------------
PAZ4_P6.abi         ----------------------------------------------------------------------------------------------------
PAZ4_P8.abi         ----------------------------------------------------------------------------------------------------
PAZ4_P10.abi        ----------------------------------------------------------------------------------------------------
PAZ4_P12.abi        ----------------------------------------------------------------------------------------------------
```

```
                                   DraI                                                   AflIII
ref/seq delPAZ_hDcr ACTGCTTCCATTTTAAATGGGAAATGTGATCCAGAGGAATTGGAAGAAAAGATTCAGAAACTAGAGAAAATTCTTAAGAGTAATGCTGAAACTGCAACTG
                     T  A  S  I  L  N  G  K  C  D  P  E  E  L  E  E  K  I  Q  K  L  E  K  I  L  K  S  N  A  E  T  A  T

PAZ4_SeqP2.abi      ----------------------------------------------------------------------------------------------------
PAZ4_1.abi          ACTGCTTCCATTTTAAATGGGAAATGTGATCCAGAGGAATTGGAAGAAAAGATTCAGAAACTAGAGAAAATTCTTAAGAGTAATGCTGAAACTGCAACTG
PAZ4_P3.abi         ----------------------------------------------------------------------------------------------------
PAZ4_P4.abi         ----------------------------------------------------------------------------------------------------
PAZ4_P6.abi         ----------------------------------------------------------------------------------------------------
PAZ4_P8.abi         ----------------------------------------------------------------------------------------------------
PAZ4_P10.abi        ----------------------------------------------------------------------------------------------------
PAZ4_P12.abi        ----------------------------------------------------------------------------------------------------
```

```
ref/seq delPAZ_hDcr ACCTGGTGGTCTTAGACAGGTATACTTCTCAGCCATGTGAGATTGTGGTGGATTGTGGACCATTTACTGACAGAAGTGGGCTTTATGAAAGACTGCTGAT
                    D  L  V  V  L  D  R  Y  T  S  Q  P  C  E  I  V  V  D  C  G  P  F  T  D  R  S  G  L  Y  E  R  L  L  M

PAZ4_SeqP2.abi      ----------------------------------------------------------------------------------------------------
PAZ4_1.abi          ACCTGGTGGTCTTAGACAGGTATACTTCTCAGCCATGTGAGATTGTGGTGGATTGTGGACCATTTACTGACAGAAGTGGGCTTTATGAAAGACTGCTGAT
PAZ4_P3.abi         ----------------------------------------------------------------------------------------------------
PAZ4_P4.abi         ----------------------------------------------------------------------------------------------------
PAZ4_P6.abi         ----------------------------------------------------------------------------------------------------
PAZ4_P8.abi         ----------------------------------------------------------------------------------------------------
PAZ4_P10.abi        ----------------------------------------------------------------------------------------------------
PAZ4_P12.abi        ----------------------------------------------------------------------------------------------------
```

```
ref/seq delPAZ_hDcr GGAATTAGAAGAAGCACTTAATTTTATCAATGATTGTAATATATCTGTACATTCAAAAGAAAGAGATTCTACTTTAATTTCGAAACAGATACTATCAGAC
                      E  L  E  E  A  L  N  F  I  N  D  C  N  I  S  V  H  S  K  E  R  D  S  T  L  I  S  K  Q  I  L  S  D

PAZ4_SeqP2.abi      ----------------------------------------------------------------------------------------------------
PAZ4_1.abi          GGAATTAGAAGAAGCACTTAATTTTATCAATGATTGTAATATATCTGTACATTCAAAAGAAAGAGATTCTACTTTAATTTCGAAACAGATACTATCAGAC
PAZ4_P3.abi         ----------------------------------------------------------------------------------------------------
PAZ4_P4.abi         ----------------------------------------------------------------------------------------------------
PAZ4_P6.abi         ----------------------------------------------------------------------------------------------------
PAZ4_P8.abi         ----------------------------------------------------------------------------------------------------
PAZ4_P10.abi        ----------------------------------------------------------------------------------------------------
PAZ4_P12.abi        ----------------------------------------------------------------------------------------------------
```

```
ref/seq delPAZ_hDcr TGTCGTGCCGTATTGGTAGTTCTGGGACCCTGGTGTGCAGATAAAGTAGCTGGAATGATGGTAAGAGAACTACAGAAATACATCAAACATGAGCAAGAGG
                     C  R  A  V  L  V  V  L  G  P  W  C  A  D  K  V  A  G  M  M  V  R  E  L  Q  K  Y  I  K  H  E  Q  E

PAZ4_SeqP2.abi      ----------------------------------------------------------------------------------------------------
PAZ4_1.abi          TGTCGTGCCGTATTGGTAGTTCTGGGACCCTGGTGTGCAGATAAAGTAGCTGGAATGATGGTAAGAGAACTACAGAAATACATCAAACATGAGCAAGAGG
PAZ4_P3.abi         ---------------------------------------------GTAGCTGGAATGATGGTAAGAGAACTACAGAAATACATCAAACATGAGCAAGAGG
PAZ4_P4.abi         ----------------------------------------------------------------------------------------------------
PAZ4_P6.abi         ----------------------------------------------------------------------------------------------------
PAZ4_P8.abi         ----------------------------------------------------------------------------------------------------
PAZ4_P10.abi        ----------------------------------------------------------------------------------------------------
PAZ4_P12.abi        ----------------------------------------------------------------------------------------------------
```

```
                                                                                  DraIII
ref/seq delPAZ_hDcr AGCTGCACAGGAAATTTTTATTGTTTACAGACACTTTCCTAAGGAAAATACATGCACTATGTGAAGAGCACTTCTCACCTGCCTCACTTGACCTGAAATT
                    E  L  H  R  K  F  L  L  F  T  D  T  F  L  R  K  I  H  A  L  C  E  E  H  F  S  P  A  S  L  D  L  K  F

PAZ4_SeqP2.abi      ----------------------------------------------------------------------------------------------------
PAZ4_1.abi          AGCTGCACAGGAAATTTTTATTGTTTACAGACACTTTCCTAAGGAAAATACATGCACTATGTGAAGAGCACTTCTCACCTGCCTCACTTGACCTGAAATT
PAZ4_P3.abi         AGCTGCACAGGAAATTTTTATTGTTTACAGACACTTTCCTAAGGAAAATACATGCACTATGTGAAGAGCACTTCTCACCTGCCTCACTTGACCTGAAATT
PAZ4_P4.abi         ----------------------------------------------------------------------------------------------------
PAZ4_P6.abi         ----------------------------------------------------------------------------------------------------
PAZ4_P8.abi         ----------------------------------------------------------------------------------------------------
PAZ4_P10.abi        ----------------------------------------------------------------------------------------------------
PAZ4_P12.abi        ----------------------------------------------------------------------------------------------------
```

NdeI

```
ref/seq delPAZ_hDcr[illegible] TGTAACTCCTAAAGTAATCAAACTGCTCGAAATCTTACGCAAATATAAACCATATGAGCGACAGCAGTTTGAAAGCGTTGAGTGGTATAATAATAGAAAT
                                V  T  P  K  V  I  K  L  L  E  I  L  R  K  Y  K  P  Y  E  R  Q  Q  F  E  S  V  E  W  Y  N  N  R  N

PAZ4_SeqP2.abi    ------------------------------------------------------------------------------------------------------
PAZ4_1.abi        TGTAACTCCTAAAGTAATCAAACTGCTCGAAATCTTACGCAAATATAAACCATATGAGCGACAGCAGTTTGAAAGCGTTGAGTGGTATAATAATAGAAAT
PAZ4_P3.abi       TGTAACTCCTAAAGTAATCAAACTGCTCGAAATCTTACGCAAATATAAACCATATGAGCGACAGCAGTTTGAAAGCGTTGAGTGGTATAATAATAGAAAT
PAZ4_P4.abi       ------------------------------------------------------------------------------------------------------
PAZ4_P6.abi       ------------------------------------------------------------------------------------------------------
PAZ4_P8.abi       ------------------------------------------------------------------------------------------------------
PAZ4_P10.abi      ------------------------------------------------------------------------------------------------------
PAZ4_P12.abi      ------------------------------------------------------------------------------------------------------
```

```
ref/seq delPAZ_hDcr[illegible] CAGGATAATTATGTGTCATGGAGTGATTCTGAGGATGATGATGAGGATGAAGAAATTGAAGAAAAAGAGAAGCCAGAGACAAATTTTCCTTCTCCTTTTA
                                Q  D  N  Y  V  S  W  S  D  S  E  D  D  D  E  D  E  E  I  E  E  K  E  K  P  E  T  N  F  P  S  P  F

PAZ4_SeqP2.abi    ------------------------------------------------------------------------------------------------------
PAZ4_1.abi        CAGGATAATTATGTGTCATGGAGTGATTCTGAGGATGATGATGAGGATGAAGAA------------------------------------------------
PAZ4_P3.abi       CAGGATAATTATGTGTCATGGAGTGATTCTGAGGATGATGATGAGGATGAAGAAATTGAAGAAAAAGAGAAGCCAGAGACAAATTTTCCTTCTCCTTTTA
PAZ4_P4.abi       ------------------------------------------------------------------------------------------------------
PAZ4_P6.abi       ------------------------------------------------------------------------------------------------------
PAZ4_P8.abi       ------------------------------------------------------------------------------------------------------
PAZ4_P10.abi      ------------------------------------------------------------------------------------------------------
PAZ4_P12.abi      ------------------------------------------------------------------------------------------------------
```

```
ref/seq delPAZ_hDcr CCAACATTTTGTGCGGAATTATTTTTGTGGAAAGAAGATACACAGCAGTTGTCTTAAACAGATTGATAAAGGAAGCTGGCAAACAAGATCCAGAGCTGGC
                    T  N  I  L  C  G  I  I  F  V  E  R  R  Y  T  A  V  V  L  N  R  L  I  K  E  A  G  K  Q  D  P  E  L  A

PAZ4_SeqP2.abi      ---------------------------------------------------------------------------------------------------
PAZ4_1.abi          ---------------------------------------------------------------------------------------------------
PAZ4_P3.abi         CCAACATTTTGTGCGGAATTATTTTTGTGGAAAGAAGATACACAGCAGTTGTCTTAAACAGATTGATAAAGGAAGCTGGCAAACAAGATCCAGAGCTGGC
PAZ4_P4.abi         ----------------------------------------------------------------------------------CAAGATCCAGAGCTGGC
PAZ4_P6.abi         ---------------------------------------------------------------------------------------------------
PAZ4_P8.abi         ---------------------------------------------------------------------------------------------------
PAZ4_P10.abi        ---------------------------------------------------------------------------------------------------
PAZ4_P12.abi        ---------------------------------------------------------------------------------------------------
```

```
                                                                                                      EcoRI
ref/seq delPAZ_hDcr TTATATCAGTAGCAATTTCATAACTGGACATGGCATTGGGAAGAATCAGCCTCGCAACAAACAGATGGAAGCAGAATTCAGAAAACAGGAAGAGGTACTT
                     Y  I  S  S  N  F  I  T  G  H  G  I  G  K  N  Q  P  R  N  K  Q  M  E  A  E  F  R  K  Q  E  E  V  L

PAZ4_SeqP2.abi      ---------------------------------------------------------------------------------------------------
PAZ4_1.abi          ---------------------------------------------------------------------------------------------------
PAZ4_P3.abi         TTATATCAGTAGCAATTTCATAACTGGACATGGCATTGGGAAGAATCAGCCTCGCAACAAACAGATGGAAGCAGAATTCAGAAAACAGGAAGAGGTACTT
PAZ4_P4.abi         TTATATCAGTAGCAATTTCATAACTGGACATGGCATTGGGAAGAATCAGCCTCGCAACAAACAGATGGAAGCAGAATTCAGAAAACAGGAAGAGGTACTT
PAZ4_P6.abi         ---------------------------------------------------------------------------------------------------
PAZ4_P8.abi         ---------------------------------------------------------------------------------------------------
PAZ4_P10.abi        ---------------------------------------------------------------------------------------------------
PAZ4_P12.abi        ---------------------------------------------------------------------------------------------------
```

```
ref/seq delPAZ_hDcr AGGAAATTTCGAGCACATGAGACCAACCTGCTTATTGCAACAAGTATTGTAGAAGAGGGTGTTGATATACCAAAATGCAACTTGGTGGTTCGTTTTGATT
                     R  K  F  R  A  H  E  T  N  L  L  I  A  T  S  I  V  E  E  G  V  D  I  P  K  C  N  L  V  V  R  F  D

PAZ4_SeqP2.abi     ----------------------------------------------------------------------------------------------------
PAZ4_1.abi         ----------------------------------------------------------------------------------------------------
PAZ4_P3.abi        AGGAAATTTCGAGCACATGAGACCAACCTGCTTATTGCAACAAGTATTGTAGAAGAGGGTGTTGATATACCAAAATGCAACTTGGTGGTTCGTTTTGATT
PAZ4_P4.abi        AGGAAATTTCGAGCACATGAGACCAACCTGCTTATTGCAACAAGTATTGTAGAAGAGGGTGTTGATATACCAAAATGCAACTTGGTGGTTCGTTTTGATT
PAZ4_P6.abi        ----------------------------------------------------------------------------------------------------
PAZ4_P8.abi        ----------------------------------------------------------------------------------------------------
PAZ4_P10.abi       ----------------------------------------------------------------------------------------------------
PAZ4_P12.abi       ----------------------------------------------------------------------------------------------------
```

```
                                 ClaI*
ref/seq delPAZ_hDcr TGCCCACAGAATATCGATCCTATGTTCAATCTAAAGGAAGAGCAAGGGCACCCATCTCTAATTATATAATGTTAGCGGATACAGACAAAATAAAAAGTTT
                    L  P  T  E  Y  R  S  Y  V  Q  S  K  G  R  A  R  A  P  I  S  N  Y  I  M  L  A  D  T  D  K  I  K  S  F

PAZ4_SeqP2.abi     ----------------------------------------------------------------------------------------------------
PAZ4_1.abi         ----------------------------------------------------------------------------------------------------
PAZ4_P3.abi        TGCCCACAGAATATCGATCCTATGTTCAATCTAAAGGAAGAGCAAGGGCACCCATCTCTAATTATATAATGTTAGCGGATACAGACAAAATAAAAAGTTT
PAZ4_P4.abi        TGCCCACAGAATATCGATCCTATGTTCAATCTAAAGGAAGAGCAAGGGCACCCATCTCTAATTATATAATGTTAGCGGATACAGACAAAATAAAAAGTTT
PAZ4_P6.abi        ----------------------------------------------------------------------------------------------------
PAZ4_P8.abi        ----------------------------------------------------------------------------------------------------
PAZ4_P10.abi       ----------------------------------------------------------------------------------------------------
PAZ4_P12.abi       ----------------------------------------------------------------------------------------------------
```

```
                                                 BglII
ref/seq delPAZ_hDcrTGAAGAAGACCTTAAAACCTACAAAGCTATTGAAAAGATCTTGAGAAACAAGTGTTCCAAGTCGGTTGATACTGGTGAGACTGACATTGATCCTGTCATG
                     E  E  D  L  K  T  Y  K  A  I  E  K  I  L  R  N  K  C  S  K  S  V  D  T  G  E  T  D  I  D  P  V  M

PAZ4_SeqP2.abi     ----------------------------------------------------------------------------------------------------
PAZ4_1.abi         ----------------------------------------------------------------------------------------------------
PAZ4_P3.abi        TGAAGAAGACCTTAAAACCTACAAAGCTATTGAAAAGATCTTGAGAAACAAGTGTTCCAAGTCGGTTGATACTGGTGAGACTGACATTGATCCTGTCATG
PAZ4_P4.abi        TGAAGAAGACCTTAAAACCTACAAAGCTATTGAAAAGATCTTGAGAAACAAGTGTTCCAAGTCGGTTGATACTGGTGAGACTGACATTGATCCTGTCATG
PAZ4_P6.abi        ----------------------------------------------------------------------------------------------------
PAZ4_P8.abi        ----------------------------------------------------------------------------------------------------
PAZ4_P10.abi       ----------------------------------------------------------------------------------------------------
PAZ4_P12.abi       ----------------------------------------------------------------------------------------------------
```

```
                                       NdeI        StuI
ref/seq delPAZ_hDcrGATGATGATGACGTTTTCCCACCATATGTGTTGAGGCCTGACGATGGTGGTCCACGAGTCACAATCAACACGGCCATTGGACACATCAATAGATACTGTG
                    D  D  D  D  V  F  P  P  Y  V  L  R  P  D  D  G  G  P  R  V  T  I  N  T  A  I  G  H  I  N  R  Y  C

PAZ4_SeqP2.abi     ----------------------------------------------------------------------------------------------------
PAZ4_1.abi         ----------------------------------------------------------------------------------------------------
PAZ4_P3.abi        GATGATGATGACGTTTTCCCACCATATGTGTTGAGGCCTGACGATGGTGGTCCACGAGTCACAATCAACACGGCCATTGGACACATCAATAGATACTGTG
PAZ4_P4.abi        GATGATGATGACGTTTTCCCACCATATGTGTTGAGGCCTGACGATGGTGGTCCACGAGTCACAATCAACACGGCCATTGGACACATCAATAGATACTGTG
PAZ4_P6.abi        ----------------------------------------------------------------------------------------------------
PAZ4_P8.abi        ----------------------------------------------------------------------------------------------------
PAZ4_P10.abi       ----------------------------------------------------------------------------------------------------
PAZ4_P12.abi       ----------------------------------------------------------------------------------------------------
```

```
ref/seq delPAZ_hDcr CTAGATTACCAAGTGATCCGTTTACTCATCTAGCTCCTAAATGCAGAACCCGAGAGTTGCCTGATGGTACATTTTATTCAACTCTTTATCTGCCAATTAA
                    A  R  L  P  S  D  P  F  T  H  L  A  P  K  C  R  T  R  E  L  P  D  G  T  F  Y  S  T  L  Y  L  P  I  N

PAZ4_SeqP2.abi      ----------------------------------------------------------------------------------------------------
PAZ4_1.abi          ----------------------------------------------------------------------------------------------------
PAZ4_P3.abi         CTAGATTACCAAGTGATCCGTTTACTCATCTAGCTCCTAAATGCAGAACCCGAGAGTTGCCTGATGGTACATTTTATTCAACTCTT--------------
PAZ4_P4.abi         CTAGATTACCAAGTGATCCGTTTACTCATCTAGCTCCTAAATGCAGAACCCGAGAGTTGCCTGATGGTACATTTTATTCAACTCTTTATCTGCCAATTAA
PAZ4_P6.abi         ----------------------------------------------------------------------------------------------------
PAZ4_P8.abi         ----------------------------------------------------------------------------------------------------
PAZ4_P10.abi        ----------------------------------------------------------------------------------------------------
PAZ4_P12.abi        ----------------------------------------------------------------------------------------------------
```

```
ref/seq delPAZ_hDcr CTCACCTCTTCGAGCCTCCATTGTTGGTCCACCAATGAGCTGTGTACGATTGGCTGAAAGAGTTGTAGCTCTCATTTGCTGTGAGAAACTGCACAAAATT
                     S  P  L  R  A  S  I  V  G  P  P  M  S  C  V  R  L  A  E  R  V  V  A  L  I  C  C  E  K  L  H  K  I

PAZ4_SeqP2.abi      ----------------------------------------------------------------------------------------------------
PAZ4_1.abi          ----------------------------------------------------------------------------------------------------
PAZ4_P3.abi         ----------------------------------------------------------------------------------------------------
PAZ4_P4.abi         CTCACCTCTTCGAGCCTCCATTGTTGGTCCACCAATGAGCTGTGTACGATTGGCTGAAAGAGTTGTAGCTCTCATTTGCTGTGAGAAACTGCACAAAATT
PAZ4_P6.abi         ----------------------------------------------------------------------------------------------------
PAZ4_P8.abi         ----------------------------------------------------------------------------------------------------
PAZ4_P10.abi        ----------------------------------------------------------------------------------------------------
PAZ4_P12.abi        ----------------------------------------------------------------------------------------------------
```

```
ref/seq delPAZ_hDcr GGCGAACTGGATGACCATTTGATGCCAGTTGGGAAAGAGACTGTTAAATATGAAGAGGAGCTTGATTTGCATGATGAAGAAGAGACCAGTGTTCCAGGAA
                     G  E  L  D  D  H  L  M  P  V  G  K  E  T  V  K  Y  E  E  E  L  D  L  H  D  E  E  E  T  S  V  P  G

PAZ4_SeqP2.abi      ----------------------------------------------------------------------------------------------------
PAZ4_1.abi          ----------------------------------------------------------------------------------------------------
PAZ4_P3.abi         ----------------------------------------------------------------------------------------------------
PAZ4_P4.abi         GGCGAACTGGATGACCATTTGATGCCAGTTGGGAAAGAGACTGTTAAATATGAAGAGGAGCTTGATTTGCATGATGAAGAAGAGACCAGTGTTCCAGGAA
PAZ4_P6.abi         ----------------------------------------------------------------------------------------------------
PAZ4_P8.abi         ----------------------------------------------------------------------------------------------------
PAZ4_P10.abi        ----------------------------------------------------------------------------------------------------
PAZ4_P12.abi        ----------------------------------------------------------------------------------------------------
```

```
                                                                                             BclI*
ref/seq delPAZ_hDcr GACCAGGTTCCACGAAACGAAGGCAGTGCTACCCAAAAGCAATTCCAGAGTGTTTGAGGGATAGTTATCCCAGACCTGATCAGCCCTGTTACCTGTATGT
                    R  P  G  S  T  K  R  R  Q  C  Y  P  K  A  I  P  E  C  L  R  D  S  Y  P  R  P  D  Q  P  C  Y  L  Y  V

PAZ4_SeqP2.abi      ----------------------------------------------------------------------------------------------------
PAZ4_1.abi          ----------------------------------------------------------------------------------------------------
PAZ4_P3.abi         ----------------------------------------------------------------------------------------------------
PAZ4_P4.abi         GACCAGGTTCCACGAAACGAAGGCAGTGCTACCCAAAAGCAATTCCAGAGTGTTTGAGGGATAGTTATCCCAGACCTGATCAGCCCTGTTACCTGTATGT
PAZ4_P6.abi         ----------------------------------------------------------------------------------------------------
PAZ4_P8.abi         ----------------------------------------------------------------------------------------------------
PAZ4_P10.abi        ----------------------------------------------------------------------------------------------------
PAZ4_P12.abi        ----------------------------------------------------------------------------------------------------
```

ref/seq delPAZ_hDcr GATAGGAATGGTTTTAACTACACCTTTACCTGATGAACTCAACTTTAGAAGGCGGAAGCTCTATCCTCCTGAAGATACCACAAGATGCTTTGGAATACTG
I G M V L T T P L P D E L N F R R R K L Y P P E D T T R C F G I L

PAZ4_SeqP2.abi --
PAZ4_1.abi --
PAZ4_P3.abi --
PAZ4_P4.abi GATAGGAATGGTTTTAACTACACCTTTACCTGATGAACTCAACTTTAGAAGGCGGAAGCTCTATCCTCCTGAAGATACCACAAGATGCTTTGGAATACTG
PAZ4_P6.abi --
PAZ4_P8.abi --
PAZ4_P10.abi --
PAZ4_P12.abi --

BstEII
ref/seq delPAZ_hDcr ACGGCCAAACCCATACCTCAGATTCCACACTTTCCTGTGTACACACGCTCTGGAGAGGTTACCATATCCATTGAGTTGAAGAAGTCTGGTTTCATGTTGT
T A K P I P Q I P H F P V Y T R S G E V T I S I E L K K S G F M L

PAZ4_SeqP2.abi --
PAZ4_1.abi --
PAZ4_P3.abi --
PAZ4_P4.abi ACGGCCAAACCCATACCTCAGATTCCACACTTTCCTGTGTACACACGCTCTGGAGAGGTTACCATATCCATTGAGTTGAAGAAGTCTGGTTTCATGTTGT
PAZ4_P6.abi --
PAZ4_P8.abi --
PAZ4_P10.abi --
PAZ4_P12.abi --

```
                                                                                                   DraI
ref/seq delPAZ_hDcr CTCTACAAATGCTTGAGTTGATTACAAGACTTCACCAGTATATATTCTCACATATTCTTCGGCTTGAAAAACCTGCACTAGAATTTAAACCTACAGACGC
                    S  L  Q  M  L  E  L  I  T  R  L  H  Q  Y  I  F  S  H  I  L  R  L  E  K  P  A  L  E  F  K  P  T  D  A

PAZ4_SeqP2.abi      ----------------------------------------------------------------------------------------------------
PAZ4_1.abi          ----------------------------------------------------------------------------------------------------
PAZ4_P3.abi         ----------------------------------------------------------------------------------------------------
PAZ4_P4.abi         CTCTACAAATGCTTGAGTTGATTACAAGACTTCACCAGTATATATTCTCACATATTCTTCGGCTTGAAAAACCTGCACTAGAATTTAAACCTACAGACGC
PAZ4_P6.abi         ----------------------------------------------------------------------------------------------------
PAZ4_P8.abi         ----------------------------------------------------------------------------------------------------
PAZ4_P10.abi        ----------------------------------------------------------------------------------------------------
PAZ4_P12.abi        ----------------------------------------------------------------------------------------------------
```

```
                                                                        BstXI            DraI
ref/seq delPAZ_hDcr TGATTCAGCATACTGTGTTCTACCTCTTAATGTTGTTAATGACTCCAGCACTTTGGATATTGACTTTAAATTCATGGAAGATATTGAGAAGTCTGAAGCT
                     D  S  A  Y  C  V  L  P  L  N  V  V  N  D  S  S  T  L  D  I  D  F  K  F  M  E  D  I  E  K  S  E  A
                                                                          PAZ domain deletion

PAZ4_SeqP2.abi      ----------------------------------------------------------------------------------------------------
PAZ4_1.abi          ----------------------------------------------------------------------------------------------------
PAZ4_P3.abi         ----------------------------------------------------------------------------------------------------
PAZ4_P4.abi         TGATTCAGCATACTGTGTTCTACCTCTTAATGTTGTTAATGACTCCAGCACTTTG---------------------------------------------
PAZ4_P6.abi         ----------------------------------------------------------------------------------------------------
PAZ4_P8.abi         ----------------------------------------------------------------------------------------------------
PAZ4_P10.abi        ----------------------------------------------------------------------------------------------------
PAZ4_P12.abi        ----------------------------------------------------------------------------------------------------
```

```
                 BsmI                                      DraI                                           EcoRV
ref/seq delPAZ_hDcr CGCATAGGCATTCCCAGTACAAAGTATACAAAAGAAACACCCTTTGTTTTTAAATTAGAAGATTACCAAGATGCCGTTATCATTCCAAGATATCGCAATT
                     R  I  G  I  P  S  T  K  Y  T  K  E  T  P  F  V  F  K  L  E  D  Y  Q  D  A  V  I  I  P  R  Y  R  N

PAZ4_SeqP2.abi      -----------------------------------------------------------------------------------------------------
PAZ4_1.abi          -----------------------------------------------------------------------------------------------------
PAZ4_P3.abi         -----------------------------------------------------------------------------------------------------
PAZ4_P4.abi         -----------------------------------------------------------------------------------------------------
PAZ4_P6.abi         -----------------------------------------------------------------------------------------------------
PAZ4_P8.abi         -----------------------------------------------------------------------------------------------------
PAZ4_P10.abi        -----------------------------------------------------------------------------------------------------
PAZ4_P12.abi        -----------------------------------------------------------------------------------------------------
```

```
                   BclI*        ClaI                                                                                SspI
ref/seq delPAZ_hDcr TTGATCAGCCTCATCGATTTTATGTAGCTGATGTGTACACTGATCTTACCCCACTCAGTAAATTTCCTTCCCCTGAGTATGAAACTTTTGCAGAATATTA
                    F  D  Q  P  H  R  F  Y  V  A  D  V  Y  T  D  L  T  P  L  S  K  F  P  S  P  E  Y  E  T  F  A  E  Y  Y

PAZ4_SeqP2.abi      -----------------------------------------------------------------------------------------------------
PAZ4_1.abi          -----------------------------------------------------------------------------------------------------
PAZ4_P3.abi         -----------------------------------------------------------------------------------------------------
PAZ4_P4.abi         -----------------------------------------------------------------------------------------------------
PAZ4_P6.abi         -----------------------------------------------------------------------------------------------------
PAZ4_P8.abi         -----------------------------------------------------------------------------------------------------
PAZ4_P10.abi        -----------------------------------------------------------------------------------------------------
PAZ4_P12.abi        -----------------------------------------------------------------------------------------------------
```

```
ref/seq delPAZ_hDcr TAAAACAAAGTACAACCTTGACCTAACCAATCTCAACCAGCCACTGCTGGATGTGGACCACACATCTTCAAGACTTAATCTTTTGACACCTCGACATTTG
                     K  T  K  Y  N  L  D  L  T  N  L  N  Q  P  L  L  D  V  D  H  T  S  S  R  L  N  L  L  T  P  R  H  L

PAZ4_SeqP2.abi      ---------------------------------------------------------------------------------------------------
PAZ4_1.abi          ---------------------------------------------------------------------------------------------------
PAZ4_P3.abi         ---------------------------------------------------------------------------------------------------
PAZ4_P4.abi         ---------------------------------------------------------------------------------------------------
PAZ4_P6.abi         ---------------------------------------------------------------------------------------------------
PAZ4_P8.abi         ---------------------------------------------------------------------------------------------------
PAZ4_P10.abi        ---------------------------------------------------------------------------------------------------
PAZ4_P12.abi        ---------------------------------------------------------------------------------------------------
```

```
                                          |Eco47III                                              |PstI
ref/seq delPAZ_hDcr AATCAGAAGGGGAAAGCGCTTCCTTTAAGCAGTGCTGAGAAGAGGAAAGCCAAATGGGAAAGTCTGCAGAATAAACAGATACTGGTTCCAGAACTCTGTG
                     N  Q  K  G  K  A  L  P  L  S  S  A  E  K  R  K  A  K  W  E  S  L  Q  N  K  Q  I  L  V  P  E  L  C

PAZ4_SeqP2.abi      ---------------------------------------------------------------------------------------------------
PAZ4_1.abi          ---------------------------------------------------------------------------------------------------
PAZ4_P3.abi         ---------------------------------------------------------------------------------------------------
PAZ4_P4.abi         ---------------------------------------------------------------------------------------------------
PAZ4_P6.abi         ---------------------------------------------------------------------------------------------------
PAZ4_P8.abi         ---------------------------------------------------------------------------------------------------
PAZ4_P10.abi        ---------------------------------------------------------------------------------------------------
PAZ4_P12.abi        ---------------------------------------------------------------------------------------------------
```

```
                                                                                           PstI
ref/seq delPAZ_hDcr CTATACATCCAATTCCAGCATCACTGTGGAGAAAAGCTGTTTGTCTCCCCAGCATACTTTATCGCCTTCACTGCCTTTTGACTGCAGAGGAGCTAAGAGC
                    A  I  H  P  I  P  A  S  L  W  R  K  A  V  C  L  P  S  I  L  Y  R  L  H  C  L  L  T  A  E  E  L  R  A
                         PAZ domain deletion

PAZ4_SeqP2.abi      ----------------------------------------------------------------------------------------------------
PAZ4_1.abi          ----------------------------------------------------------------------------------------------------
PAZ4_P3.abi         ----------------------------------------------------------------------------------------------------
PAZ4_P4.abi         ----------------------------------------------------------------------------------------------------
PAZ4_P6.abi         -----------ATTCCAGCATCACTGTGGAGAAAAGCTGTTTGTCTCCCCAGCATACTTTATCGCCTTCACTGCCTTTTGACTGCAGAGGAGCTAAGAGC
PAZ4_P8.abi         ----------------------------------------------------------------------------------------------------
PAZ4_P10.abi        ----------------------------------------------------------------------------------------------------
PAZ4_P12.abi        ----------------------------------------------------------------------------------------------------
```

```
ref/seq delPAZ_hDcr CCAGACTGCCAGCGATGCTGGCGTGGGAGTCAGATCACTTCCTGCGGATTTTAGATACCCTAACTTAGACTTCGGGTGGAAAAAATCTATTGACAGCAAA
                     Q  T  A  S  D  A  G  V  G  V  R  S  L  P  A  D  F  R  Y  P  N  L  D  F  G  W  K  K  S  I  D  S  K

PAZ4_SeqP2.abi      ----------------------------------------------------------------------------------------------------
PAZ4_1.abi          ----------------------------------------------------------------------------------------------------
PAZ4_P3.abi         ----------------------------------------------------------------------------------------------------
PAZ4_P4.abi         ----------------------------------------------------------------------------------------------------
PAZ4_P6.abi         CCAGACTGCCAGCGATGCTGGCGTGGGAGTCAGATCACTTCCTGCGGATTTTAGATACCCTAACTTAGACTTCGGGTGGAAAAAATCTATTGACAGCAAA
PAZ4_P8.abi         ---------------------------------------------------------------------------------------TCTATTGACAGCAAA
PAZ4_P10.abi        ----------------------------------------------------------------------------------------------------
PAZ4_P12.abi        ----------------------------------------------------------------------------------------------------
```

```
                                  PvuII                         MunI
ref/seq delPAZ_hDcr TCTTTCATCTCAATTTCTAACTCCTCTTCAGCTGAAAATGATAATTACTGTAAGCACAGCACAATTGTCCCTGAAAATGCTGCACATCAAGGTGCTAATA
                     S  F  I  S  I  S  N  S  S  S  A  E  N  D  N  Y  C  K  H  S  T  I  V  P  E  N  A  A  H  Q  G  A  N

PAZ4_SeqP2.abi      ---------------------------------------------------------------------------------------------------
PAZ4_1.abi          ---------------------------------------------------------------------------------------------------
PAZ4_P3.abi         ---------------------------------------------------------------------------------------------------
PAZ4_P4.abi         ---------------------------------------------------------------------------------------------------
PAZ4_P6.abi         TCTTTCATCTCAATTTCTAACTCCTCTTCAGCTGAAAATGATAATTACTGTAAGCACAGCACAATTGTCCCTGAAAATGCTGCACATCAAGGTGCTAATA
PAZ4_P8.abi         TCTTTCATCTCAATTTCTAACTCCTCTTCAGCTGAAAATGATAATTACTGTAAGCACAGCACAATTGTCCCTGAAAATGCTGCACATCAAGGTGCTAATA
PAZ4_P10.abi        ---------------------------------------------------------------------------------------------------
PAZ4_P12.abi        ---------------------------------------------------------------------------------------------------
```

```
                                                            AclI
                               XbaI                     PstI                                                  BglII
ref/seq delPAZ_hDcr GAACCTCCTCTCTAGAAAATCATGACCAAATGTCTGTGAACTGCAGAACGTTGCTCAGCGAGTCCCCTGGTAAGCTCCACGTTGAAGTTTCAGCAGATCT
                    R  T  S  S  L  E  N  H  D  Q  M  S  V  N  C  R  T  L  L  S  E  S  P  G  K  L  H  V  E  V  S  A  D  L

PAZ4_SeqP2.abi      ---------------------------------------------------------------------------------------------------
PAZ4_1.abi          ---------------------------------------------------------------------------------------------------
PAZ4_P3.abi         ---------------------------------------------------------------------------------------------------
PAZ4_P4.abi         ---------------------------------------------------------------------------------------------------
PAZ4_P6.abi         GAACCTCCTCTCTAGAAAATCATGACCAAATGTCTGTGAACTGCAGAACGTTGCTCAGCGAGTCCCCTGGTAAGCTCCACGTTGAAGTTTCAGCAGATCT
PAZ4_P8.abi         GAACCTCCTCTCTAGAAAATCATGACCAAATGTCTGTGAACTGCAGAACGTTGCTCAGCGAGTCCCCTGGTAAGCTCCACGTTGAAGTTTCAGCAGATCT
PAZ4_P10.abi        ---------------------------------------------------------------------------------------------------
PAZ4_P12.abi        ---------------------------------------------------------------------------------------------------
```

```
                   VspI
ref/seq delPAZ_hDcrTACAGCAATTAATGGTCTTTCTTACAATCAAAATCTCGCCAATGGCAGTTATGATTTAGCTAACAGAGACTTTTGCCAAGGAAATCAGCTAAATTACTAC
                    T  A  I  N  G  L  S  Y  N  Q  N  L  A  N  G  S  Y  D  L  A  N  R  D  F  C  Q  G  N  Q  L  N  Y  Y

PAZ4_SeqP2.abi     ----------------------------------------------------------------------------------------------------
PAZ4_1.abi         ----------------------------------------------------------------------------------------------------
PAZ4_P3.abi        ----------------------------------------------------------------------------------------------------
PAZ4_P4.abi        ----------------------------------------------------------------------------------------------------
PAZ4_P6.abi        TACAGCAATTAATGGTCTTTCTTACAATCAAAATCTCGCCAATGGCAGTTATGATTTAGCTAACAGAGACTTTTGCCAAGGAAATCAGCTAAATTACTAC
PAZ4_P8.abi        TACAGCAATTAATGGTCTTTCTTACAATCAAAATCTCGCCAATGGCAGTTATGATTTAGCTAACAGAGACTTTTGCCAAGGAAATCAGCTAAATTACTAC
PAZ4_P10.abi       ----------------------------------------------------------------------------------------------------
PAZ4_P12.abi       ----------------------------------------------------------------------------------------------------
```

```
ref/seq delPAZ_hDcrAAGCAGGAAATACCCGTGCAACCAACTACCTCATATTCCATTCAGAATTTATACAGTTACGAGAACCAGCCCCAGCCCAGCGATGAATGTACTCTCCTGA
                   K  Q  E  I  P  V  Q  P  T  T  S  Y  S  I  Q  N  L  Y  S  Y  E  N  Q  P  Q  P  S  D  E  C  T  L  L

PAZ4_SeqP2.abi     ----------------------------------------------------------------------------------------------------
PAZ4_1.abi         ----------------------------------------------------------------------------------------------------
PAZ4_P3.abi        ----------------------------------------------------------------------------------------------------
PAZ4_P4.abi        ----------------------------------------------------------------------------------------------------
PAZ4_P6.abi        AAGCAGGAAATACCCGTGCAACCAACTACCTCATATTCCATTCAGAATTTATACAGTTACGAGAACCAGCCCCAGCCCAGCGATGAATGTACTCTCCTGA
PAZ4_P8.abi        AAGCAGGAAATACCCGTGCAACCAACTACCTCATATTCCATTCAGAATTTATACAGTTACGAGAACCAGCCCCAGCCCAGCGATGAATGTACTCTCCTGA
PAZ4_P10.abi       ----------------------------------------------------------------------------------------------------
PAZ4_P12.abi       ----------------------------------------------------------------------------------------------------
```

```
ref/seq delPAZ_hDcr GTAATAAATACCTTGATGGAAATGCTAACAAATCTACCTCAGATGGAAGTCCTGTGATGGCCGTAATGCCTGGTACGACAGACACTATTCAAGTGCTCAA
                    S  N  K  Y  L  D  G  N  A  N  K  S  T  S  D  G  S  P  V  M  A  V  M  P  G  T  T  D  T  I  Q  V  L  K

PAZ4_SeqP2.abi      ----------------------------------------------------------------------------------------------------
PAZ4_1.abi          ----------------------------------------------------------------------------------------------------
PAZ4_P3.abi         ----------------------------------------------------------------------------------------------------
PAZ4_P4.abi         ----------------------------------------------------------------------------------------------------
PAZ4_P6.abi         GTAATAAATACCTTGATGGAAATGCTAACAAATCTACCTCAGATGGAAGTCCTGTGATGGCCGTAATGCCTGGTACGACAGACACTATTCAAGTGCTCAA
PAZ4_P8.abi         GTAATAAATACCTTGATGGAAATGCTAACAAATCTACCTCAGATGGAAGTCCTGTGATGGCCGTAATGCCTGGTACGACAGACACTATTCAAGTGCTCAA
PAZ4_P10.abi        ----------------------------------------------------------------------------------------------------
PAZ4_P12.abi        ----------------------------------------------------------------------------------------------------
```

```
                                                                                                 Tth111I
ref/seq delPAZ_hDcr GGGCAGGATGGATTCTGAGCAGAGCCCTTCTATTGGGTACTCCTCAAGGACTCTTGGCCCCAATCCTGGACTTATTCTTCAGGCTTTGACTCTGTCAAAC
                      G  R  M  D  S  E  Q  S  P  S  I  G  Y  S  S  R  T  L  G  P  N  P  G  L  I  L  Q  A  L  T  L  S  N

PAZ4_SeqP2.abi      ----------------------------------------------------------------------------------------------------
PAZ4_1.abi          ----------------------------------------------------------------------------------------------------
PAZ4_P3.abi         ----------------------------------------------------------------------------------------------------
PAZ4_P4.abi         ----------------------------------------------------------------------------------------------------
PAZ4_P6.abi         GGGCAGGATGGATTCTGAGCAGAGCCCTTCTATTGGGTACTCCTCAAGGACTCTTGGCCCCAATCCTGGACTTATTCTTCAG------------------
PAZ4_P8.abi         GGGCAGGATGGATTCTGAGCAGAGCCCTTCTATTGGGTACTCCTCAAGGACTCTTGGCCCCAATCCTGGACTTATTCTTCAGGCTTTGACTCTGTCAAAC
PAZ4_P10.abi        ----------------------------------------------------------------------------------------------------
PAZ4_P12.abi        ----------------------------------------------------------------------------------------------------
```

```
                                                                                   DraI       SphI
ref/seq delPAZ_hDcr GCTAGTGATGGATTTAACCTGGAGCGGCTTGAAATGCTTGGCGACTCCTTTTTAAAGCATGCCATCACCACATATCTATTTTGCACTTACCCTGATGCGC
                     A  S  D  G  F  N  L  E  R  L  E  M  L  G  D  S  F  L  K  H  A  I  T  T  Y  L  F  C  T  Y  P  D  A

PAZ4_SeqP2.abi      ----------------------------------------------------------------------------------------------------
PAZ4_1.abi          ----------------------------------------------------------------------------------------------------
PAZ4_P3.abi         ----------------------------------------------------------------------------------------------------
PAZ4_P4.abi         ----------------------------------------------------------------------------------------------------
PAZ4_P6.abi         ----------------------------------------------------------------------------------------------------
PAZ4_P8.abi         GCTAGTGATGGATTTAACCTGGAGCGGCTTGAAATGCTTGGCGACTCCTTTTTAAAGCATGCCATCACCACATATCTATTTTGCACTTACCCTGATGCGC
PAZ4_P10.abi        ----------------------------------------------------------------------------------------------------
PAZ4_P12.abi        ----------------------------------------------------------------------------------------------------
```

```
                                                                                                      BstXI
ref/seq delPAZ_hDcr ATGAGGGCCGCCTTTCATATATGAGAAGCAAAAAGGTCAGCAACTGTAATCTGTATCGCCTTGGAAAAAAGAAGGGACTACCCAGCCGCATGGTGGTGTC
                    H  E  G  R  L  S  Y  M  R  S  K  K  V  S  N  C  N  L  Y  R  L  G  K  K  K  G  L  P  S  R  M  V  V  S

PAZ4_SeqP2.abi      ----------------------------------------------------------------------------------------------------
PAZ4_1.abi          ----------------------------------------------------------------------------------------------------
PAZ4_P3.abi         ----------------------------------------------------------------------------------------------------
PAZ4_P4.abi         ----------------------------------------------------------------------------------------------------
PAZ4_P6.abi         ----------------------------------------------------------------------------------------------------
PAZ4_P8.abi         ATGAGGGCCGCCTTTCATATATGAGAAGCAAAAAGGTCAGCAACTGTAATCTGTATCGCCTTGGAAAAAAGAAGGGACTACCCAGCCGCATGGTGGTGTC
PAZ4_P10.abi        ----------------------------------------------------------------------------------------------------
PAZ4_P12.abi        ----------------------------------------------------------------------------------------------------
```

```
                       SspI
ref/seq delPAZ_hDcr AATATTTGATCCCCCTGTGAATTGGCTTCCTCCTGGTTATGTAGTAAATCAAGACAAAAGCAACACAGATAAATGGGAAAAAGATGAAATGACAAAAGAC
                     I  F  D  P  P  V  N  W  L  P  P  G  Y  V  V  N  Q  D  K  S  N  T  D  K  W  E  K  D  E  M  T  K  D

PAZ4_SeqP2.abi      ----------------------------------------------------------------------------------------------------
PAZ4_1.abi          ----------------------------------------------------------------------------------------------------
PAZ4_P3.abi         ----------------------------------------------------------------------------------------------------
PAZ4_P4.abi         ----------------------------------------------------------------------------------------------------
PAZ4_P6.abi         ----------------------------------------------------------------------------------------------------
PAZ4_P8.abi         AATATTTGATCCCCCTGTGAATTGGCTTCCTCCTGGTTATGTAGTAAATCAAGACAAAAGCAACACAGATAAATGGGAAAAAGATGAAATGACAAAAGAC
PAZ4_P10.abi        ----------------------------------------------------------------------------------------------------
PAZ4_P12.abi        ----------------------------------------------------------------------------------------------------
```

```
                         SphI
ref/seq delPAZ_hDcr TGCATGCTGGCGAATGGCAAACTGGATGAGGATTACGAGGAGGAGGATGAGGAGGAGGAGAGCCTGATGTGGAGGGCTCCGAAGGAAGAGGCTGACTATG
                     C  M  L  A  N  G  K  L  D  E  D  Y  E  E  E  D  E  E  E  E  S  L  M  W  R  A  P  K  E  E  A  D  Y

PAZ4_SeqP2.abi      ----------------------------------------------------------------------------------------------------
PAZ4_1.abi          ----------------------------------------------------------------------------------------------------
PAZ4_P3.abi         ----------------------------------------------------------------------------------------------------
PAZ4_P4.abi         ----------------------------------------------------------------------------------------------------
PAZ4_P6.abi         ----------------------------------------------------------------------------------------------------
PAZ4_P8.abi         TGCATGCTGGCGAATGGCAAACTGGATGAGGATTACGAGGAGGAGGATGAGGAGGAGGAGAGCCTGATGTGGAGGGCTCCGAAGGAAGAGGCTGACTATG
PAZ4_P10.abi        ---------------------CTGGATGAGGATTACGAGGAGGAGGATGAGGAGGAGGAGAGCCTGATGTGGAGGGCTCCGAAGGAAGAGGCTGACTATG
PAZ4_P12.abi        ----------------------------------------------------------------------------------------------------
```

```
                                  BclI*
ref/seq delPAZ_hDcr AAGATGATTTCCTGGAGTATGATCAGGAACATATCAGATTTATAGATAATATGTTAATGGGGTCAGGAGCTTTTGTAAAGAAAATCTCTCTTTCTCCTTT
                     E  D  D  F  L  E  Y  D  Q  E  H  I  R  F  I  D  N  M  L  M  G  S  G  A  F  V  K  K  I  S  L  S  P  F

PAZ4_SeqP2.abi      ---------------------------------------------------------------------------------------------------
PAZ4_1.abi          ---------------------------------------------------------------------------------------------------
PAZ4_P3.abi         ---------------------------------------------------------------------------------------------------
PAZ4_P4.abi         ---------------------------------------------------------------------------------------------------
PAZ4_P6.abi         ---------------------------------------------------------------------------------------------------
PAZ4_P8.abi         AAGATGATTTCCTGGAGTATGATCAGGAACATATCAGATTTATAGATAATATGTTAATGGGGTCAGGAGCTTTTGTAAAGAAAATCTCTCTTTCTCCTTT
PAZ4_P10.abi        AAGATGATTTCCTGGAGTATGATCAGGAACATATCAGATTTATAGATAATATGTTAATGGGGTCAGGAGCTTTTGTAAAGAAAATCTCTCTTTCTCCTTT
PAZ4_P12.abi        ---------------------------------------------------------------------------------------------------
```

```
                                NdeI
ref/seq delPAZ_hDcr TTCAACCACTGATTCTGCATATGAATGGAAAATGCCCAAAAAATCCTCCTTAGGTAGTATGCCATTTTCATCAGATTTTGAGGATTTTGACTACAGCTCT
                      S  T  T  D  S  A  Y  E  W  K  M  P  K  K  S  S  L  G  S  M  P  F  S  S  D  F  E  D  F  D  Y  S  S

PAZ4_SeqP2.abi      ---------------------------------------------------------------------------------------------------
PAZ4_1.abi          ---------------------------------------------------------------------------------------------------
PAZ4_P3.abi         ---------------------------------------------------------------------------------------------------
PAZ4_P4.abi         ---------------------------------------------------------------------------------------------------
PAZ4_P6.abi         ---------------------------------------------------------------------------------------------------
PAZ4_P8.abi         TTCAACCACTGATTCTGCATATGAATGGAAAATGCCCAAAAAATCCTCCTTAGGTAGTATGCCATTTTCATCAGATTTTGAGGATTTTGACTACAGCTCT
PAZ4_P10.abi        TTCAACCACTGATTCTGCATATGAATGGAAAATGCCCAAAAAATCCTCCTTAGGTAGTATGCCATTTTCATCAGATTTTGAGGATTTTGACTACAGCTCT
PAZ4_P12.abi        ---------------------------------------------------------------------------------------------------
```

```
                             BamHI
ref/seq delPAZ_hDcr TGGGATGCAATGTGCTATCTGGATCCTAGCAAAGCTGTTGAAGAAGATGACTTTGTGGTGGGGTTCTGGAATCCATCAGAAGAAAACTGTGGTGTTGACA
                     W  D  A  M  C  Y  L  D  P  S  K  A  V  E  E  D  D  F  V  V  G  F  W  N  P  S  E  E  N  C  G  V  D

PAZ4_SeqP2.abi      ----------------------------------------------------------------------------------------------------
PAZ4_1.abi          ----------------------------------------------------------------------------------------------------
PAZ4_P3.abi         ----------------------------------------------------------------------------------------------------
PAZ4_P4.abi         ----------------------------------------------------------------------------------------------------
PAZ4_P6.abi         ----------------------------------------------------------------------------------------------------
PAZ4_P8.abi         TGGGATGCAATGTGCTATCTGGATCCTAGCAAAGCTGTTGAAGAA-------------------------------------------------------
PAZ4_P10.abi        TGGGATGCAATGTGCTATCTGGATCCTAGCAAAGCTGTTGAAGAAGATGACTTTGTGGTGGGGTTCTGGAATCCATCAGAAGAAAACTGTGGTGTTGACA
PAZ4_P12.abi        ----------------------------------------------------------------------------------------------------
```

```
ref/seq delPAZ_hDcr CGGGAAAGCAGTCCATTTCTTACGACTTGCACACTGAGCAGTGTATTGCTGACAAAAGCATAGCGGACTGTGTGGAAGCCCTGCTGGGCTGCTATTTAAC
                    T  G  K  Q  S  I  S  Y  D  L  H  T  E  Q  C  I  A  D  K  S  I  A  D  C  V  E  A  L  L  G  C  Y  L  T

PAZ4_SeqP2.abi      ----------------------------------------------------------------------------------------------------
PAZ4_1.abi          ----------------------------------------------------------------------------------------------------
PAZ4_P3.abi         ----------------------------------------------------------------------------------------------------
PAZ4_P4.abi         ----------------------------------------------------------------------------------------------------
PAZ4_P6.abi         ----------------------------------------------------------------------------------------------------
PAZ4_P8.abi         ----------------------------------------------------------------------------------------------------
PAZ4_P10.abi        CGGGAAAGCAGTCCATTTCTTACGACTTGCACACTGAGCAGTGTATTGCTGACAAAAGCATAGCGGACTGTGTGGAAGCCCTGCTGGGCTGCTATTTAAC
PAZ4_P12.abi        ----------------------------------------------------------------------------------------------------
```

```
                            PvuII
ref/seq delPAZ_hDcr CAGCTGTGGGGAGAGGGCTGCTCAGCTTTTCCTCTGTTCACTGGGGCTGAAGGTGCTCCCGGTAATTAAAAGGACTGATCGGGAAAAGGCCCTGTGCCCT
                     S  C  G  E  R  A  A  Q  L  F  L  C  S  L  G  L  K  V  L  P  V  I  K  R  T  D  R  E  K  A  L  C  P

PAZ4_SeqP2.abi      --------------------------------------------------------------------------------------------------
PAZ4_1.abi          --------------------------------------------------------------------------------------------------
PAZ4_P3.abi         --------------------------------------------------------------------------------------------------
PAZ4_P4.abi         --------------------------------------------------------------------------------------------------
PAZ4_P6.abi         --------------------------------------------------------------------------------------------------
PAZ4_P8.abi         --------------------------------------------------------------------------------------------------
PAZ4_P10.abi        CAGCTGTGGGGAGAGGGCTGCTCAGCTTTTCCTCTGTTCACTGGGGCTGAAGGTGCTCCCGGTAATTAAAAGGACTGATCGGGAAAAGGCCCTGTGCCCT
PAZ4_P12.abi        --------------------------------------------------------------------------------------------------
```

```
                                                                                  BalI
ref/seq delPAZ_hDcr ACTCGGGAGAATTTCAACAGCCAACAAAAGAACCTTTCAGTGAGCTGTGCTGCTGCTTCTGTGGCCAGTTCACGCTCTTCTGTATTGAAAGACTCGGAAT
                     T  R  E  N  F  N  S  Q  Q  K  N  L  S  V  S  C  A  A  A  S  V  A  S  S  R  S  S  V  L  K  D  S  E

PAZ4_SeqP2.abi      --------------------------------------------------------------------------------------------------
PAZ4_1.abi          --------------------------------------------------------------------------------------------------
PAZ4_P3.abi         --------------------------------------------------------------------------------------------------
PAZ4_P4.abi         --------------------------------------------------------------------------------------------------
PAZ4_P6.abi         --------------------------------------------------------------------------------------------------
PAZ4_P8.abi         --------------------------------------------------------------------------------------------------
PAZ4_P10.abi        ACTCGGGAGAATTTCAACAGCCAACAAAAGAACCTTTCAGTGAGCTGTGCTGCTGCTTCTGTGGCCAGTTCACGCTCTTCTGTATTGAAAGACTCGGAAT
PAZ4_P12.abi        --------------------------------------------------------------------------------------------------
```

```
                              BclI*
ref/seq delPAZ_hDcr ATGGTTGTTTGAAGATTCCACCAAGATGTATGTTTGATCATCCAGATGCAGATAAAACACTGAATCACCTTATATCGGGGTTTGAAAATTTTGAAAAGAA
                    Y  G  C  L  K  I  P  P  R  C  M  F  D  H  P  D  A  D  K  T  L  N  H  L  I  S  G  F  E  N  F  E  K  K

PAZ4_SeqP2.abi      ----------------------------------------------------------------------------------------------------
PAZ4_1.abi          ----------------------------------------------------------------------------------------------------
PAZ4_P3.abi         ----------------------------------------------------------------------------------------------------
PAZ4_P4.abi         ----------------------------------------------------------------------------------------------------
PAZ4_P6.abi         ----------------------------------------------------------------------------------------------------
PAZ4_P8.abi         ----------------------------------------------------------------------------------------------------
PAZ4_P10.abi        ATGGTTGTTTGAAGATTCCACCAAGATGTATGTTTGATCATCCAGATGCAGATAAAACACTGAATCACCTTATATCGGGGTTTGAAAATTTTGAAAAGAA
PAZ4_P12.abi        ----------------------------------------------------------------------------------------------------
```

```
                                                                                                  Eco47III Ec
ref/seq delPAZ_hDcr AATCAACTACAGATTCAAGAATAAGGCTTACCTTCTCCAGGCTTTTACACATGCCTCCTACCACTACAATACTATCACTGATTGTTACCAGCGCTTAGAA
                     I  N  Y  R  F  K  N  K  A  Y  L  L  Q  A  F  T  H  A  S  Y  H  Y  N  T  I  T  D  C  Y  Q  R  L  E

PAZ4_SeqP2.abi      ----------------------------------------------------------------------------------------------------
PAZ4_1.abi          ----------------------------------------------------------------------------------------------------
PAZ4_P3.abi         ----------------------------------------------------------------------------------------------------
PAZ4_P4.abi         ----------------------------------------------------------------------------------------------------
PAZ4_P6.abi         ----------------------------------------------------------------------------------------------------
PAZ4_P8.abi         ----------------------------------------------------------------------------------------------------
PAZ4_P10.abi        AATCAACTACAGATTCAAGAATAAGGCTTACCTTCTCCAGGCTTTTACACATGCCTCCTACCACTACAATACTATCACTGATTGTTACCAGCGCTTAGAA
PAZ4_P12.abi        ----------------------------------------------------------------------------------------------------
```

```
                   oRI                                                          SacII          XmaI  SmaI
ref/seq delPAZ_hDcr TTCCTGGGAGATGCGATTTTGGACTACCTCATAACCAAGCACCTTTATGAAGACCCGCGGCAGCACTCCCCGGGGGTCCTGACAGACCTGCGGTCTGCCC
                     F  L  G  D  A  I  L  D  Y  L  I  T  K  H  L  Y  E  D  P  R  Q  H  S  P  G  V  L  T  D  L  R  S  A

PAZ4_SeqP2.abi      ---------------------------------------------------------------------------------------------------
PAZ4_1.abi          ---------------------------------------------------------------------------------------------------
PAZ4_P3.abi         ---------------------------------------------------------------------------------------------------
PAZ4_P4.abi         ---------------------------------------------------------------------------------------------------
PAZ4_P6.abi         ---------------------------------------------------------------------------------------------------
PAZ4_P8.abi         ---------------------------------------------------------------------------------------------------
PAZ4_P10.abi        TTCCTGGGAGATGCGATTTTGGACTACCTCATAACCAAGCACCTTTATGAAGACCCGCGGCAGCACTCCCCGGGGGTCCTGACAGACCTGCGGTCTGCCC
PAZ4_P12.abi        ---------------------------------------------------------------------------------------------------
```

```
                                                                                 ScaI                 Bpu10I EcoICRI SacI
ref/seq delPAZ_hDcr TGGTCAACAACACCATCTTTGCATCGCTGGCTGTAAAGTACGACTACCACAAGTACTTCAAAGCTGTCTCTCCTGAGCTCTTCCATGTCATTGATGACTT
                    L  V  N  N  T  I  F  A  S  L  A  V  K  Y  D  Y  H  K  Y  F  K  A  V  S  P  E  L  F  H  V  I  D  D  F

PAZ4_SeqP2.abi      ----------------------------------------------------------------------------------------------------
PAZ4_1.abi          ----------------------------------------------------------------------------------------------------
PAZ4_P3.abi         ----------------------------------------------------------------------------------------------------
PAZ4_P4.abi         ----------------------------------------------------------------------------------------------------
PAZ4_P6.abi         ----------------------------------------------------------------------------------------------------
PAZ4_P8.abi         ----------------------------------------------------------------------------------------------------
PAZ4_P10.abi        TGGTCAACAACACCATCTTTGCATCGCTGGCTGTAAAGTACGACTACCACAAGTACTTCAAAGCTGTCTCTCCTGAGCTCTTCCATGTCATTGATGACTT
PAZ4_P12.abi        --------------CATCTTTGCATCGCTGGCTGTAAAGTACGACTACCACAAGTACTTCAAAGCTGTCTCTCCTGAGCTCTTCCATGTCATTGATGACTT
```

```
                              Bpu10I  BglII
ref/seq delPAZ_hDcr TGTGCAGTTTCAGCTTGAGAAGAATGAAATGCAAGGAATGGATTCTGAGCTTAGGAGATCTGAGGAGGATGAAGAGAAAGAAGAGGATATTGAAGTTCCA
                     V  Q  F  Q  L  E  K  N  E  M  Q  G  M  D  S  E  L  R  R  S  E  E  D  E  E  K  E  E  D  I  E  V  P

PAZ4_SeqP2.abi      ----------------------------------------------------------------------------------------------------
PAZ4_1.abi          ----------------------------------------------------------------------------------------------------
PAZ4_P3.abi         ----------------------------------------------------------------------------------------------------
PAZ4_P4.abi         ----------------------------------------------------------------------------------------------------
PAZ4_P6.abi         ----------------------------------------------------------------------------------------------------
PAZ4_P8.abi         ----------------------------------------------------------------------------------------------------
PAZ4_P10.abi        TGTGCAGTTTCAGCTTGAGAAGAATGAAATGCAAGGAATGGATTCTGAGCTTAGGAGATCTGAGGAGGATGAAGAGA-----------------------
PAZ4_P12.abi        TGTGCAGTTTCAGCTTGAGAAGAATGAAATGCAAGGAATGGATTCTGAGCTTAGGAGATCTGAGGAGGATGAAGAGAAAGAAGAGGATATTGAAGTTCCA
```

```
                    NcoI                                     BstXI
ref/seq delPAZ_hDcr AAGGCCATGGGGGATATTTTTGAGTCGCTTGCTGGTGCCATTTACATGGATAGTGGGATGTCACTGGAGACAGTCTGGCAGGTGTACTATCCCATGATGC
                    K  A  M  G  D  I  F  E  S  L  A  G  A  I  Y  M  D  S  G  M  S  L  E  T  V  W  Q  V  Y  Y  P  M  M

PAZ4_SeqP2.abi      ----------------------------------------------------------------------------------------------------
PAZ4_1.abi          ----------------------------------------------------------------------------------------------------
PAZ4_P3.abi         ----------------------------------------------------------------------------------------------------
PAZ4_P4.abi         ----------------------------------------------------------------------------------------------------
PAZ4_P6.abi         ----------------------------------------------------------------------------------------------------
PAZ4_P8.abi         ----------------------------------------------------------------------------------------------------
PAZ4_P10.abi        ----------------------------------------------------------------------------------------------------
PAZ4_P12.abi        AAGGCCATGGGGGATATTTTTGAGTCGCTTGCTGGTGCCATTTACATGGATAGTGGGATGTCACTGGAGACAGTCTGGCAGGTGTACTATCCCATGATGC
```

```
ref/seq delPAZ_hDcr GGCCACTAATAGAAAAGTTTTCTGCAAATGTACCCCGTTCCCCTGTGCGAGAATTGCTTGAAATGGAACCAGAAACTGCCAAATTTAGCCCGGCTGAGAG
                    R  P  L  I  E  K  F  S  A  N  V  P  R  S  P  V  R  E  L  L  E  M  E  P  E  T  A  K  F  S  P  A  E  R

PAZ4_SeqP2.abi      ----------------------------------------------------------------------------------------------------
PAZ4_1.abi          ----------------------------------------------------------------------------------------------------
PAZ4_P3.abi         ----------------------------------------------------------------------------------------------------
PAZ4_P4.abi         ----------------------------------------------------------------------------------------------------
PAZ4_P6.abi         ----------------------------------------------------------------------------------------------------
PAZ4_P8.abi         ----------------------------------------------------------------------------------------------------
PAZ4_P10.abi        ----------------------------------------------------------------------------------------------------
PAZ4_P12.abi        GGCCACTAATAGAAAAGTTTTCTGCAAATGTACCCCGTTCCCCTGTGCGAGAATTGCTTGAAATGGAACCAGAAACTGCCAAATTTAGCCCGGCTGAGAG
```

```
                                                                                 DraI                                      PstI
ref/seq delPAZ_hDcr AACTTACGACGGGAAGGTCAGAGTCACTGTGGAAGTAGTAGGAAAGGGGAAATTTAAAGGTGTTGGTCGAAGTTACAGGATTGCCAAATCTGCAGCAGCA
                      T  Y  D  G  K  V  R  V  T  V  E  V  V  G  K  G  K  F  K  G  V  G  R  S  Y  R  I  A  K  S  A  A  A

PAZ4_SeqP2.abi      ----------------------------------------------------------------------------------------------------
PAZ4_1.abi          ----------------------------------------------------------------------------------------------------
PAZ4_P3.abi         ----------------------------------------------------------------------------------------------------
PAZ4_P4.abi         ----------------------------------------------------------------------------------------------------
PAZ4_P6.abi         ----------------------------------------------------------------------------------------------------
PAZ4_P8.abi         ----------------------------------------------------------------------------------------------------
PAZ4_P10.abi        ----------------------------------------------------------------------------------------------------
PAZ4_P12.abi        AACTTACGACGGGAAGGTCAGAGTCACTGTGGAAGTAGTAGGAAAGGGGAAATTTAAAGGTGTTGGTCGAAGTTACAGGATTGCCAAATCTGCAGCAGCA
```

```
ref/seq delPAZ_hDcr AGAAGAGCCCTCCGAAGCCTCAAAGCTAATCAACCTCAGGTTCCCAATAGCGGTGGCGGAGGTTCTGGAGGCGGTGGAAGTGACTACAAGGACGACGATG
                     R  R  A  L  R  S  L  K  A  N  Q  P  Q  V  P  N  S  G  G  G  G  S  G  G  G  G  S  D  Y  K  D  D  D

PAZ4_SeqP2.abi      ----------------------------------------------------------------------------------------------------
PAZ4_1.abi          ----------------------------------------------------------------------------------------------------
PAZ4_P3.abi         ----------------------------------------------------------------------------------------------------
PAZ4_P4.abi         ----------------------------------------------------------------------------------------------------
PAZ4_P6.abi         ----------------------------------------------------------------------------------------------------
PAZ4_P8.abi         ----------------------------------------------------------------------------------------------------
PAZ4_P10.abi        ----------------------------------------------------------------------------------------------------
PAZ4_P12.abi        AGAAGAGCCCTCCGAAGCCTCAAAGCTAATCAACCTCAGGTTCCCAATAGCGGTGGCGGAGGTTCTGGAGGCGGTGGAAGTGACTACAAGGACGACGATG
```

```
                                                                                               XhoI
ref/seq delPAZ_hDcr ACAAGGATTACAAAGACGATGATGACAAGGACTATAAGGACGATGACGATAAGTAACATTTGGTTTAGTGTACAATATCTCCTCGAGCATTTGGTTTAGT
                    D  K  D  Y  K  D  D  D  D  K  D  Y  K  D  D  D  D  K  *  H  L  V  *  C  T  I  S  P  R  A  F  G  L  V
                                                                           Stop

PAZ4_SeqP2.abi      ----------------------------------------------------------------------------------------------------
PAZ4_1.abi          ----------------------------------------------------------------------------------------------------
PAZ4_P3.abi         ----------------------------------------------------------------------------------------------------
PAZ4_P4.abi         ----------------------------------------------------------------------------------------------------
PAZ4_P6.abi         ----------------------------------------------------------------------------------------------------
PAZ4_P8.abi         ----------------------------------------------------------------------------------------------------
PAZ4_P10.abi        ----------------------------------------------------------------------------------------------------
PAZ4_P12.abi        ACAAGGATTACAAAGACGATGATGACAAGGACTATAAGGACGATGACGATAAGTAACATTTGGTTTAGTGTACAATATCTCCTCGA--------------
```

```
                          XhoI
ref/seq delPAZ_hDcrGTACAATATCTCCTCGAGGGCTCGTGGCCTCGACTGTGCCTTCTAGTTGCCAGCCATCTGTTGTTTGC
                     Y  N  I  S  S  R  A  R  G  L  D  C  A  F  *  L  P  A  I  C  C  L

PAZ4_SeqP2.abi     ----------------------------------------------------------------------
PAZ4_1.abi         ----------------------------------------------------------------------
PAZ4_P3.abi        ----------------------------------------------------------------------
PAZ4_P4.abi        ----------------------------------------------------------------------
PAZ4_P6.abi        ----------------------------------------------------------------------
PAZ4_P8.abi        ----------------------------------------------------------------------
PAZ4_P10.abi       ----------------------------------------------------------------------
PAZ4_P12.abi       ----------------------------------------------------------------------
```

```
ref/seq delPAZ_hDcr

PAZ4_SeqP2.abi
PAZ4_1.abi
PAZ4_P3.abi
PAZ4_P4.abi
PAZ4_P6.abi
PAZ4_P8.abi
PAZ4_P10.abi
PAZ4_P12.abi
```

```
                                   AatII
                                 ZraI
ref/seq delPPC_hDcr CCAAGTCTCCACCCCATTGACGTCAATGGGAGTTTGTTTTGGCACCAAAATCAACGGGACTTTCCAAAATGTCGTAACAACTCCGCCCCATTGACGCAAA
                     P  S  L  H  P  I  D  V  N  G  S  L  F  W  H  Q  N  Q  R  D  F  P  K  C  R  N  N  S  A  P  L  T  Q

mK2kol10_SqrP2.ab   CCAAGTCTCCACCCCATTGACGTCAATGGGAGTTTGTTTTGGCACCAAAATCAACGGGACTTTCCAAAATGTCGTAACAACTCCGCCCCATTGACGCAAA
mK2kol10_1.abi      ----------------------------------------------------------------------------------------------------
mK2kol10_P3.abi     ----------------------------------------------------------------------------------------------------
mK2kol10II_P3.abi   ----------------------------------------------------------------------------------------------------
mK2kol10_P4.abi     ----------------------------------------------------------------------------------------------------
mK2kol10II_P4.abi   ----------------------------------------------------------------------------------------------------
mK2kol10_P5.abi     ----------------------------------------------------------------------------------------------------
mK2kol10_P8.abi     ----------------------------------------------------------------------------------------------------
mK2kol10_P10.abi    ----------------------------------------------------------------------------------------------------
mK2kol10_P12.abi    ----------------------------------------------------------------------------------------------------
```

```
                                                                                               BglII
ref/seq delPPC_hDcr TGGGCGGTAGGCGTGTACGGTGGGAGGTCTATATAAGCAGAGCTGGTTTAGTGAACCGTCAGATCAGATCTTTGTCGATCCTACCATCCACTCGACACAC
                    M  G  G  R  R  V  R  W  E  V  Y  I  S  R  A  G  L  V  N  R  Q  I  R  S  L  S  I  L  P  S  T  R  H  T
                    Start

mK2kol10_SqrP2.ab   TGGGCGGTAGGCGTGTACGGTGGGAGGTCTATATAAGCAGAGCTGGTTTAGTGAACCGTCAGATCAGATCTTTGTCGATCCTACCATCCACTCGACACAC
mK2kol10_1.abi      ----------------------------------------------------------------------------------------------------
mK2kol10_P3.abi     ----------------------------------------------------------------------------------------------------
mK2kol10II_P3.abi   ----------------------------------------------------------------------------------------------------
mK2kol10_P4.abi     ----------------------------------------------------------------------------------------------------
mK2kol10II_P4.abi   ----------------------------------------------------------------------------------------------------
mK2kol10_P5.abi     ----------------------------------------------------------------------------------------------------
mK2kol10_P8.abi     ----------------------------------------------------------------------------------------------------
mK2kol10_P10.abi    ----------------------------------------------------------------------------------------------------
mK2kol10_P12.abi    ----------------------------------------------------------------------------------------------------
```

```
                   SacI                   KpnI
      NotI    HindIII    EcoICRI EcoRI   Acc65I      DraI
ref/seq delPPC_hDcr CCGCCAGCGGCCGCTGCCAAGCTTCCGAGCTCTCGAATTCAAAGGAGGTACCCACCCTTGTTTAAACTTTAAGAGGAGGGCCACCATGAAAAGCCCTGCT
                     R  Q  R  P  L  P  S  F  R  A  L  E  F  K  G  G  T  H  P  C  L  N  F  K  R  R  A  T  M  K  S  P  A

mK2kol10_SqrP2.ab   CCGCCAGCGGCCGCTGCCAAGCTTCCGAGCTCTCGAATTCAAAGGAGGTACCCACCCTTGTTTAAACTTTAAGAGGAGGGCCACCATGAAAAGCCCTGCT
mK2kol10_1.abi      ----------------------------------------------------------------------------------------------------
mK2kol10_P3.abi     ----------------------------------------------------------------------------------------------------
mK2kol10II_P3.abi   ----------------------------------------------------------------------------------------------------
mK2kol10_P4.abi     ----------------------------------------------------------------------------------------------------
mK2kol10II_P4.abi   ----------------------------------------------------------------------------------------------------
mK2kol10_P5.abi     ----------------------------------------------------------------------------------------------------
mK2kol10_P8.abi     ----------------------------------------------------------------------------------------------------
mK2kol10_P10.abi    ----------------------------------------------------------------------------------------------------
mK2kol10_P12.abi    ----------------------------------------------------------------------------------------------------
```

```
          Bpu10I        StuI  PstI                                           NcoI
ref/seq delPPC_hDcr TTGCAACCCCTCAGCATGGCAGGCCTGCAGCTCATGACCCCTGCTTCCTCACCAATGGGTCCTTTCTTTGGACTGCCATGGCAACAAGAAGCAATTCATG
                     L  Q  P  L  S  M  A  G  L  Q  L  M  T  P  A  S  S  P  M  G  P  F  F  G  L  P  W  Q  Q  E  A  I  H

mK2kol10_SqrP2.ab   TTGCAACCCCTCAGCATGGCAGGCCTGCAGCTCATGACCCCTGCTTCCTCACCAATGGGTCCTTTCTTTGGACTGCCATGGCAACAAGAAGCAATTCATG
mK2kol10_1.abi      ------------------------CTGCAGCTCATGACCCCTGCTTCCTCACCAATGGGTCCTTTCTTTGGACTGCCATGGCAACAAGAAGCAATTCATG
mK2kol10_P3.abi     ----------------------------------------------------------------------------------------------------
mK2kol10II_P3.abi   ----------------------------------------------------------------------------------------------------
mK2kol10_P4.abi     ----------------------------------------------------------------------------------------------------
mK2kol10II_P4.abi   ----------------------------------------------------------------------------------------------------
mK2kol10_P5.abi     ----------------------------------------------------------------------------------------------------
mK2kol10_P8.abi     ----------------------------------------------------------------------------------------------------
mK2kol10_P10.abi    ----------------------------------------------------------------------------------------------------
mK2kol10_P12.abi    ----------------------------------------------------------------------------------------------------
```

```
                                                                                         DraI        Bpu10I
ref/seq delPPC_hDcr ATAACATTTATACGCCAAGAAAATATCAGGTTGAACTGCTTGAAGCAGCTCTGGATCATAATACCATCGTCTGTTTAAACACTGGCTCAGGGAAGACATT
                     D  N  I  Y  T  P  R  K  Y  Q  V  E  L  L  E  A  A  L  D  H  N  T  I  V  C  L  N  T  G  S  G  K  T  F

mK2kol10_SqrP2.ab   ATAACATTTATACGCCAAGAAAATATCAGGTTGAACTGCTTGAAGCAGCTCTGGATCATAATACCATCGTCTGTTTAAACACTGGCTCAGGGAAGACATT
mK2kol10_1.abi      ATAACATTTATACGCCAAGAAAATATCAGGTTGAACTGCTTGAAGCAGCTCTGGATCATAATACCATCGTCTGTTTAAACACTGGCTCAGGGAAGACATT
mK2kol10_P3.abi     ---------------------------------------------------------------------------------------------------
mK2kol10II_P3.abi   ---------------------------------------------------------------------------------------------------
mK2kol10_P4.abi     ---------------------------------------------------------------------------------------------------
mK2kol10II_P4.abi   ---------------------------------------------------------------------------------------------------
mK2kol10_P5.abi     ---------------------------------------------------------------------------------------------------
mK2kol10_P8.abi     ---------------------------------------------------------------------------------------------------
mK2kol10_P10.abi    ---------------------------------------------------------------------------------------------------
mK2kol10_P12.abi    ---------------------------------------------------------------------------------------------------
```

```
                             ScaI
ref/seq delPPC_hDcr TATTGCAGTACTACTCACTAAAGAGCTGTCCTATCAGATCAGGGGAGACTTCAGCAGAAATGGAAAAAGGACGGTGTTCTTGGTCAACTCTGCAAACCAG
                       I  A  V  L  L  T  K  E  L  S  Y  Q  I  R  G  D  F  S  R  N  G  K  R  T  V  F  L  V  N  S  A  N  Q

mK2kol10_SqrP2.ab   TATTGCAGTACTACTCACTAAAGAGCTGTCCTATCAGATCAGGGGAGACTTCAGCAGAAATGGAAAAAGGACGGTGTTCTTGGTCAACTCTGCAAACCAG
mK2kol10_1.abi      TATTGCAGTACTACTCACTAAAGAGCTGTCCTATCAGATCAGGGGAGACTTCAGCAGAAATGGAAAAAGGACGGTGTTCTTGGTCAACTCTGCAAACCAG
mK2kol10_P3.abi     ---------------------------------------------------------------------------------------------------
mK2kol10II_P3.abi   ---------------------------------------------------------------------------------------------------
mK2kol10_P4.abi     ---------------------------------------------------------------------------------------------------
mK2kol10II_P4.abi   ---------------------------------------------------------------------------------------------------
mK2kol10_P5.abi     ---------------------------------------------------------------------------------------------------
mK2kol10_P8.abi     ---------------------------------------------------------------------------------------------------
mK2kol10_P10.abi    ---------------------------------------------------------------------------------------------------
mK2kol10_P12.abi    ---------------------------------------------------------------------------------------------------
```

```
                  PvuII         BglII                                NsiI
ref/seq delPPC_hDcr GTTGCTCAACAAGTGTCAGCTGTCAGAACTCATTCAGATCTCAAGGTTGGGGAATACTCAAACCTAGAAGTAAATGCATCTTGGACAAAAGAGAGATGGA
                     V  A  Q  Q  V  S  A  V  R  T  H  S  D  L  K  V  G  E  Y  S  N  L  E  V  N  A  S  W  T  K  E  R  W

mK2kol10_SqrP2.ab GTTGCTCAACAAGTGTCAGCTGTCAGAACTCATTCAGATCTCAAGGTTGGGGAATACTCAAAC------------------------------------
mK2kol10_1.abi    GTTGCTCAACAAGTGTCAGCTGTCAGAACTCATTCAGATCTCAAGGTTGGGGAATACTCAAACCTAGAAGTAAATGCATCTTGGACAAAAGAGAGATGGA
mK2kol10_P3.abi   ----------------------------------------------------------------------------------------------------
mK2kol10II_P3.abi ----------------------------------------------------------------------------------------------------
mK2kol10_P4.abi   ----------------------------------------------------------------------------------------------------
mK2kol10II_P4.abi ----------------------------------------------------------------------------------------------------
mK2kol10_P5.abi   ----------------------------------------------------------------------------------------------------
mK2kol10_P8.abi   ----------------------------------------------------------------------------------------------------
mK2kol10_P10.abi  ----------------------------------------------------------------------------------------------------
mK2kol10_P12.abi  ----------------------------------------------------------------------------------------------------
```

```
ref/seq delPPC_hDcr ACCAAGAGTTTACTAAGCACCAGGTTCTCATTATGACTTGCTATGTCGCCTTGAATGTTTTGAAAAATGGTTACTTATCACTGTCAGACATTAACCTTTT
                    N  Q  E  F  T  K  H  Q  V  L  I  M  T  C  Y  V  A  L  N  V  L  K  N  G  Y  L  S  L  S  D  I  N  L  L

mK2kol10_SqrP2.ab ----------------------------------------------------------------------------------------------------
mK2kol10_1.abi    ACCAAGAGTTTACTAAGCACCAGGTTCTCATTATGACTTGCTATGTCGCCTTGAATGTTTTGAAAAATGGTTACTTATCACTGTCAGACATTAACCTTTT
mK2kol10_P3.abi   ----------------------------------------------------------------------------------------------------
mK2kol10II_P3.abi ----------------------------------------------------------------------------------------------------
mK2kol10_P4.abi   ----------------------------------------------------------------------------------------------------
mK2kol10II_P4.abi ----------------------------------------------------------------------------------------------------
mK2kol10_P5.abi   ----------------------------------------------------------------------------------------------------
mK2kol10_P8.abi   ----------------------------------------------------------------------------------------------------
mK2kol10_P10.abi  ----------------------------------------------------------------------------------------------------
mK2kol10_P12.abi  ----------------------------------------------------------------------------------------------------
```

```
ref/seq delPPC_hDcr GGTGTTTGATGAGTGTCATCTTGCAATCCTAGACCACCCCTATCGAGAAATTATGAAGCTCTGTGAAAATTGTCCATCATGTCCTCGCATTTTGGGACTA
                      V  F  D  E  C  H  L  A  I  L  D  H  P  Y  R  E  I  M  K  L  C  E  N  C  P  S  C  P  R  I  L  G  L

mK2kol10_SqrP2.ab   ----------
mK2kol10_1.abi      GGTGTTTGATGAGTGTCATCTTGCAATCCTAGACCACCCCTATCGAGAAATTATGAAGCTCTGTGAAAATTGTCCATCATGTCCTCGCATTTTGGGACTA
mK2kol10_P3.abi     ----------
mK2kol10II_P3.abi   ----------
mK2kol10_P4.abi     ----------
mK2kol10II_P4.abi   ----------
mK2kol10_P5.abi     ----------
mK2kol10_P8.abi     ----------
mK2kol10_P10.abi    ----------
mK2kol10_P12.abi    ----------
```

```
                                   DraI                                                 AflII
ref/seq delPPC_hDcr ACTGCTTCCATTTTAAATGGGAAATGTGATCCAGAGGAATTGGAAGAAAAGATTCAGAAACTAGAGAAAATTCTTAAGAGTAATGCTGAAACTGCAACTG
                     T  A  S  I  L  N  G  K  C  D  P  E  E  L  E  E  K  I  Q  K  L  E  K  I  L  K  S  N  A  E  T  A  T

mK2kol10_SqrP2.ab   ----------
mK2kol10_1.abi      ACTGCTTCCATTTTAAATGGGAAATGTGATCCAGAGGAATTGGAAGAAAAGATTCAGAAACTAGAGAAAATTCTTAAGAGTAATGCTGAAACTGCAACTG
mK2kol10_P3.abi     ----------
mK2kol10II_P3.abi   ----------
mK2kol10_P4.abi     ----------
mK2kol10II_P4.abi   ----------
mK2kol10_P5.abi     ----------
mK2kol10_P8.abi     ----------
mK2kol10_P10.abi    ----------
mK2kol10_P12.abi    ----------
```

```
ref/seq delPPC_hDcr ACCTGGTGGTCTTAGACAGGTATACTTCTCAGCCATGTGAGATTGTGGTGGATTGTGGACCATTTACTGACAGAAGTGGGCTTTATGAAAGACTGCTGAT
                    D  L  V  V  L  D  R  Y  T  S  Q  P  C  E  I  V  V  D  C  G  P  F  T  D  R  S  G  L  Y  E  R  L  L  M

mK2kol10_SqrP2.ab   -----------------------------------------------------------------------------------------------------
mK2kol10_1.abi      ACCTGGTGGTCTTAGACAGGTATACTTCTCAGCCATGTGAGATTGTGGTGGATTGTGGACCATTTACTGACAGAAGTGGGCTTTATGAAAGACTGCTGAT
mK2kol10_P3.abi     -----------------------------------------------------------------------------------------------------
mK2kol10II_P3.abi   -----------------------------------------------------------------------------------------------------
mK2kol10_P4.abi     -----------------------------------------------------------------------------------------------------
mK2kol10II_P4.abi   -----------------------------------------------------------------------------------------------------
mK2kol10_P5.abi     -----------------------------------------------------------------------------------------------------
mK2kol10_P8.abi     -----------------------------------------------------------------------------------------------------
mK2kol10_P10.abi    -----------------------------------------------------------------------------------------------------
mK2kol10_P12.abi    -----------------------------------------------------------------------------------------------------
```

```
ref/seq delPPC_hDcr GGAATTAGAAGAAGCACTTAATTTTATCAATGATTGTAATATATCTGTACATTCAAAAGAAAGAGATTCTACTTTAATTTCGAAACAGATACTATCAGAC
                     E  L  E  E  A  L  N  F  I  N  D  C  N  I  S  V  H  S  K  E  R  D  S  T  L  I  S  K  Q  I  L  S  D

mK2kol10_SqrP2.ab   -----------------------------------------------------------------------------------------------------
mK2kol10_1.abi      GGAATTAGAAGAAGCACTTAATTTTATCAATGATTGTAATATATCTGTACATTCAAAAGAAAGAGATTCTACTTTAATTTCGAAACAGATACTATCAGAC
mK2kol10_P3.abi     -----------------------------------------------------------------------------------------------------
mK2kol10II_P3.abi   -----------------------------------------------------------------------------------------------------
mK2kol10_P4.abi     -----------------------------------------------------------------------------------------------------
mK2kol10II_P4.abi   -----------------------------------------------------------------------------------------------------
mK2kol10_P5.abi     -----------------------------------------------------------------------------------------------------
mK2kol10_P8.abi     -----------------------------------------------------------------------------------------------------
mK2kol10_P10.abi    -----------------------------------------------------------------------------------------------------
mK2kol10_P12.abi    -----------------------------------------------------------------------------------------------------
```

```
ref/seq delPPC_hDcr TGTCGTGCCGTATTGGTAGTTCTGGGACCCTGGTGTGCAGATAAAGTAGCTGGAATGATGGTAAGAGAACTACAGAAATACATCAAACATGAGCAAGAGG
                     C  R  A  V  L  V  V  L  G  P  W  C  A  D  K  V  A  G  M  M  V  R  E  L  Q  K  Y  I  K  H  E  Q  E

mK2kol10_SqrP2.ab   ----------------------------------------------------------------------------------------------------
mK2kol10_1.abi      TGTCGTGCCGTATTGGTAGTTCTGGGACCCTGGTGTGCAGATAAAGTAGCTGGAATGATGGTAAGAGAACTACAGAAATACATCAAACATGAGCAAGAGG
mK2kol10_P3.abi     ---------------------------------------------GTAGCTGGAATGATGGTAAGAGAACTACAGAAATACATCAAACATGAGCAAGAGG
mK2kol10II_P3.abi   -------------------------------------------------------TGATGGTAAGAGAACTACAGAAATACATCAAACATGAGCAAGAGG
mK2kol10_P4.abi     ----------------------------------------------------------------------------------------------------
mK2kol10II_P4.abi   ----------------------------------------------------------------------------------------------------
mK2kol10_P5.abi     ----------------------------------------------------------------------------------------------------
mK2kol10_P8.abi     ----------------------------------------------------------------------------------------------------
mK2kol10_P10.abi    ----------------------------------------------------------------------------------------------------
mK2kol10_P12.abi    ----------------------------------------------------------------------------------------------------
```

```
                                                                DraIII
ref/seq delPPC_hDcr AGCTGCACAGGAAATTTTTATTGTTTACAGACACTTTCCTAAGGAAAATACATGCACTATGTGAAGAGCACTTCTCACCTGCCTCACTTGACCTGAAATT
                    E  L  H  R  K  F  L  L  F  T  D  T  F  L  R  K  I  H  A  L  C  E  E  H  F  S  P  A  S  L  D  L  K  F

mK2kol10_SqrP2.ab   ----------------------------------------------------------------------------------------------------
mK2kol10_1.abi      AGCTGCACAGGAAATTTTTATTGTTTACAGACACTTTCCTAAGGAAAATACATGCACTATGTGAAGAGCACTTCTCACCTGCCTCACTTGACCTGAAATT
mK2kol10_P3.abi     AGCTGCACAGGAAATTTTTATTGTTTACAGACACTTTCCTAAGGAAAATACATGCACTATGTGAAGAGCACTTCTCACCTGCCTCACTTGACCTGAAATT
mK2kol10II_P3.abi   AGCTGCACAGGAAATTTTTATTGTTTACAGACACTTTCCTAAGGAAAATACATGCACTATGTGAAGAGCACTTCTCACCTGCCTCACTTGACCTGAAATT
mK2kol10_P4.abi     ----------------------------------------------------------------------------------------------------
mK2kol10II_P4.abi   ----------------------------------------------------------------------------------------------------
mK2kol10_P5.abi     ----------------------------------------------------------------------------------------------------
mK2kol10_P8.abi     ----------------------------------------------------------------------------------------------------
mK2kol10_P10.abi    ----------------------------------------------------------------------------------------------------
mK2kol10_P12.abi    ----------------------------------------------------------------------------------------------------
```

```
                        NdeI
ref/seq delPPC_hDcr TGTAACTCCTAAAGTAATCAAACTGCTCGAAATCTTACGCAAATATAAACCATATGAGCGACAGCAGTTTGAAAGCGTTGAGTGGTATAATAATAGAAAT
                     V  T  P  K  V  I  K  L  L  E  I  L  R  K  Y  K  P  Y  E  R  Q  Q  F  E  S  V  E  W  Y  N  N  R  N

mK2kol10_SqrP2.ab   ----------------------------------------------------------------------------------------------------
mK2kol10_1.abi      TGTAACTCCTAAAGTAATCAAACTGCTCGAAATCTTACGCAA----------------------------------------------------------
mK2kol10_P3.abi     TGTAACTCCTAAAGTAATCAAACTGCTCGAAATCTTACGCAAATATAAACCATATGAGCGACAGCAGTTTGAAAGCGTTGAGTGGTATAATAATAGAAAT
mK2kol10II_P3.abi   TGTAACTCCTAAAGTAATCAAACTGCTCGAAATCTTACGCAAATATAAACCATATGAGCGACAGCAGTTTGAAAGCGTTGAGTGGTATAATAATAGAAAT
mK2kol10_P4.abi     ----------------------------------------------------------------------------------------------------
mK2kol10II_P4.abi   ----------------------------------------------------------------------------------------------------
mK2kol10_P5.abi     ----------------------------------------------------------------------------------------------------
mK2kol10_P8.abi     ----------------------------------------------------------------------------------------------------
mK2kol10_P10.abi    ----------------------------------------------------------------------------------------------------
mK2kol10_P12.abi    ----------------------------------------------------------------------------------------------------
```

```
ref/seq delPPC_hDcr CAGGATAATTATGTGTCATGGAGTGATTCTGAGGATGATGATGAGGATGAAGAAATTGAAGAAAAAGAGAAGCCAGAGACAAATTTTCCTTCTCCTTTTA
                     Q  D  N  Y  V  S  W  S  D  S  E  D  D  D  E  D  E  E  I  E  E  K  E  K  P  E  T  N  F  P  S  P  F

mK2kol10_SqrP2.ab   ----------------------------------------------------------------------------------------------------
mK2kol10_1.abi      ----------------------------------------------------------------------------------------------------
mK2kol10_P3.abi     CAGGATAATTATGTGTCATGGAGTGATTCTGAGGATGATGATGAGGATGAAGAAATTGAAGAAAAAGAGAAGCCAGAGACAAATTTTCCTTCTCCTTTTA
mK2kol10II_P3.abi   CAGGATAATTATGTGTCATGGAGTGATTCTGAGGATGATGATGAGGATGAAGAAATTGAAGAAAAAGAGAAGCCAGAGACAAATTTTCCTTCTCCTTTTA
mK2kol10_P4.abi     ----------------------------------------------------------------------------------------------------
mK2kol10II_P4.abi   ----------------------------------------------------------------------------------------------------
mK2kol10_P5.abi     ----------------------------------------------------------------------------------------------------
mK2kol10_P8.abi     ----------------------------------------------------------------------------------------------------
mK2kol10_P10.abi    ----------------------------------------------------------------------------------------------------
mK2kol10_P12.abi    ----------------------------------------------------------------------------------------------------
```

```
ref/seq delPPC_hDcr  CCAACATTTTGTGCGGAATTATTTTTGTGGAAAGAAGATACACAGCAGTTGTCTTAAACAGATTGATAAAGGAAGCTGGCAAACAAGATCCAGAGCTGGC
                      T  N  I  L  C  G  I  I  F  V  E  R  R  Y  T  A  V  V  L  N  R  L  I  K  E  A  G  K  Q  D  P  E  L  A

mK2kol10_SqrP2.ab    ----------------------------------------------------------------------------------------------------
mK2kol10_1.abi       ----------------------------------------------------------------------------------------------------
mK2kol10_P3.abi      CCAACATTTTGTGCGGAATTATTTTTGTGGAAAGAAGATACACAGCAGTTGTCTTAAACAGATTGATAAAGGAAGCTGGCAAACAAGATCCAGAGCTGGC
mK2kol10II_P3.abi    CCAACATTTTGTGC--------------------------------------------------------------------------------------
mK2kol10_P4.abi      ---------------------------------------------------------------------------------------ATCCAGAGCTGGC
mK2kol10II_P4.abi    -----------------------------------------------------------------------------------CAAGATCCAGAGCTGGC
mK2kol10_P5.abi      ----------------------------------------------------------------------------------------------------
mK2kol10_P8.abi      ----------------------------------------------------------------------------------------------------
mK2kol10_P10.abi     ----------------------------------------------------------------------------------------------------
mK2kol10_P12.abi     ----------------------------------------------------------------------------------------------------
```

```
                                                                                  EcoRI
ref/seq delPPC_hDcr  TTATATCAGTAGCAATTTCATAACTGGACATGGCATTGGGAAGAATCAGCCTCGCAACAAACAGATGGAAGCAGAATTCAGAAAACAGGAAGAGGTACTT
                       Y  I  S  S  N  F  I  T  G  H  G  I  G  K  N  Q  P  R  N  K  Q  M  E  A  E  F  R  K  Q  E  E  V  L

mK2kol10_SqrP2.ab    ----------------------------------------------------------------------------------------------------
mK2kol10_1.abi       ----------------------------------------------------------------------------------------------------
mK2kol10_P3.abi      TTATATCAGTAGCAATTTCATAACTGGACATGGCATTGGGAAGAATCAGCCTCGCAACAAACAGATGGAAGCAGAATTCAGAAAACAGGAAGAGGTACTT
mK2kol10II_P3.abi    ----------------------------------------------------------------------------------------------------
mK2kol10_P4.abi      TTATATCAGTAGCAATTTCATAACTGGACATGGCATTGGGAAGAATCAGCCTCGCAACAAACAGATGGAAGCAGAATTCAGAAAACAGGAAGAGGTACTT
mK2kol10II_P4.abi    TTATATCAGTAGCAATTTCATAACTGGACATGGCATTGGGAAGAATCAGCCTCGCAACAAACAGATGGAAGCAGAATTCAGAAAACAGGAAGAGGTACTT
mK2kol10_P5.abi      ----------------------------------------------------------------------------------------------------
mK2kol10_P8.abi      ----------------------------------------------------------------------------------------------------
mK2kol10_P10.abi     ----------------------------------------------------------------------------------------------------
mK2kol10_P12.abi     ----------------------------------------------------------------------------------------------------
```

```
ref/seq delPPC_hDcr AGGAAATTTCGAGCACATGAGACCAACCTGCTTATTGCAACAAGTATTGTAGAAGAGGGTGTTGATATACCAAAATGCAACTTGGTGGTTCGTTTTGATT
                     R  K  F  R  A  H  E  T  N  L  L  I  A  T  S  I  V  E  E  G  V  D  I  P  K  C  N  L  V  V  R  F  D

mK2kol10_SqrP2.ab   ----------------------------------------------------------------------------------------------------
mK2kol10_1.abi      ----------------------------------------------------------------------------------------------------
mK2kol10_P3.abi     AGGAAATTTCGAGCACATGAGACCAACCTGCTTATTGCAACAAGTATTGTAGAAGAGGGTGTTGATATACCAAAATGCAACTTGGTGGTTCGTTTTGATT
mK2kol10II_P3.abi   ----------------------------------------------------------------------------------------------------
mK2kol10_P4.abi     AGGAAATTTCGAGCACATGAGACCAACCTGCTTATTGCAACAAGTATTGTAGAAGAGGGTGTTGATATACCAAAATGCAACTTGGTGGTTCGTTTTGATT
mK2kol10II_P4.abi   AGGAAATTTCGAGCACATGAGACCAACCTGCTTATTGCAACAAGTATTGTAGAAGAGGGTGTTGATATACCAAAATGCAACTTGGTGGTTCGTTTTGATT
mK2kol10_P5.abi     ----------------------------------------------------------------------------------------------------
mK2kol10_P8.abi     ----------------------------------------------------------------------------------------------------
mK2kol10_P10.abi    ----------------------------------------------------------------------------------------------------
mK2kol10_P12.abi    ----------------------------------------------------------------------------------------------------
```

```
                                  ClaI*
ref/seq delPPC_hDcr TGCCCACAGAATATCGATCCTATGTTCAATCTAAAGGAAGAGCAAGGGCACCCATCTCTAATTATATAATGTTAGCGGATACAGACAAAATAAAAAGTTT
                    L  P  T  E  Y  R  S  Y  V  Q  S  K  G  R  A  R  A  P  I  S  N  Y  I  M  L  A  D  T  D  K  I  K  S  F

mK2kol10_SqrP2.ab   ----------------------------------------------------------------------------------------------------
mK2kol10_1.abi      ----------------------------------------------------------------------------------------------------
mK2kol10_P3.abi     TGCCCACAGAATATCGATCCTATGTTCAATCTAAAGGAAGAGCAAGGGCACCCATCTCTAATTATATAATGTTAGCGGATACAGACAAAATAAAAAGTTT
mK2kol10II_P3.abi   ----------------------------------------------------------------------------------------------------
mK2kol10_P4.abi     TGCCCACAGAATATCGATCCTATGTTCAATCTAAAGGAAGAGCAAGGGCACCCATCTCTAATTATATAATGTTAGCGGATACAGACAAAATAAAAAGTTT
mK2kol10II_P4.abi   TGCCCACAGAATATCGATCCTATGTTCAATCTAAAGGAAGAGCAAGGGCACCCATCTCTAATTATATAATGTTAGCGGATACAGACAAAATAAAAAGTTT
mK2kol10_P5.abi     ----------------------------------------------------------------------------------------------------
mK2kol10_P8.abi     ----------------------------------------------------------------------------------------------------
mK2kol10_P10.abi    ----------------------------------------------------------------------------------------------------
mK2kol10_P12.abi    ----------------------------------------------------------------------------------------------------
```

```
                                                    BglII
ref/seq delPPC_hDcr TGAAGAAGACCTTAAAACCTACAAAGCTATTGAAAAGATCTTGAGAAACAAGTGTTCCAAGTCGGTTGATACTGGTGAGACTGACATTGATCCTGTCATG
                     E  E  D  L  K  T  Y  K  A  I  E  K  I  L  R  N  K  C  S  K  S  V  D  T  G  E  T  D  I  D  P  V  M

mK2kol10_SqrP2.ab   ----------------------------------------------------------------------------------------------------
mK2kol10_1.abi      ----------------------------------------------------------------------------------------------------
mK2kol10_P3.abi     TGAAGAAGACCTTAAAACCTACAAAGCTATTGAAAAGATCTTGAGAAACAAGTGTTCCAAGTCGGTTGATACTGGTGAGACTGACATTGATCCTGTCATG
mK2kol10II_P3.abi   ----------------------------------------------------------------------------------------------------
mK2kol10_P4.abi     TGAAGAAGACCTTAAAACCTACAAAGCTATTGAAAAGATCTTGAGAAACAAGTGTTCCAAGTCGGTTGATACTGGTGAGACTGACATTGATCCTGTCATG
mK2kol10II_P4.abi   TGAAGAAGACCTTAAAACCTACAAAGCTATTGAAAAGATCTTGAGAAACAAGTGTTCCAAGTCGGTTGATACTGGTGAGACTGACATTGATCCTGTCATG
mK2kol10_P5.abi     ----------------------------------------------------------------------------------------------------
mK2kol10_P8.abi     ----------------------------------------------------------------------------------------------------
mK2kol10_P10.abi    ----------------------------------------------------------------------------------------------------
mK2kol10_P12.abi    ----------------------------------------------------------------------------------------------------
```

```
                                            NdeI        StuI
ref/seq delPPC_hDcr GATGATGATGACGTTTTCCCACCATATGTGTTGAGGCCTGACGATGGTGGTCCACGAGTCACAATCAACACGGCCATTGGACACATCAATAGATACTGTG
                     D  D  D  D  V  F  P  P  Y  V  L  R  P  D  D  G  G  P  R  V  T  I  N  T  A  I  G  H  I  N  R  Y  C

mK2kol10_SqrP2.ab   ----------------------------------------------------------------------------------------------------
mK2kol10_1.abi      ----------------------------------------------------------------------------------------------------
mK2kol10_P3.abi     GATGATGATGACGTTTTCCCA-------------------------------------------------------------------------------
mK2kol10II_P3.abi   ----------------------------------------------------------------------------------------------------
mK2kol10_P4.abi     GATGATGATGACGTTTTCCCACCATATGTGTTGAGGCCTGACGATGGTGGTCCACGAGTCACAATCAACACGGCCATTGGACACATCAATAGATACTGTG
mK2kol10II_P4.abi   GATGATGATGACGTTTTCCCACCATATGTGTTGAGGCCTGACGATGGTGGTCCACGAGTCACAATCAACACGGCCATTGGACACATCAATAGATACTGTG
mK2kol10_P5.abi     ----------------------------------------------------------------------------------------------------
mK2kol10_P8.abi     ----------------------------------------------------------------------------------------------------
mK2kol10_P10.abi    ----------------------------------------------------------------------------------------------------
mK2kol10_P12.abi    ----------------------------------------------------------------------------------------------------
```

```
ref/seq delPPC_hDcr CTAGATTACCAAGTGATCCGTTTACTCATCTAGCTCCTAAATGCAGAACCCGAGAGTTGCCTGATGGTACATTTTATTCAACTCTTTATCTGCCAATTAA
                    A  R  L  P  S  D  P  F  T  H  L  A  P  K  C  R  T  R  E  L  P  D  G  T  F  Y  S  T  L  Y  L  P  I  N

mK2kol10_SqrP2.ab   ----------------------------------------------------------------------------------------------------
mK2kol10_1.abi      ----------------------------------------------------------------------------------------------------
mK2kol10_P3.abi     ----------------------------------------------------------------------------------------------------
mK2kol10II_P3.abi   ----------------------------------------------------------------------------------------------------
mK2kol10_P4.abi     CTAGATTACCAAGTGATCCGTTTACTCATCTAGCTCCTAAATGCAGAACCCGAGAGTTGCCTGATGGTACATTTTATTCAACTCTTTATCTGCCAATTAA
mK2kol10II_P4.abi   CTAGATTACCAAGTGATCCGTTTACTCATCTAGCTCCTAAATGCAGAACCCGAGAGTTGCCTGATGGTACATTTTATTCAACTCTTTATCTGCCAATTAA
mK2kol10_P5.abi     ----------------------------------------------------------------------------------------------------
mK2kol10_P8.abi     ----------------------------------------------------------------------------------------------------
mK2kol10_P10.abi    ----------------------------------------------------------------------------------------------------
mK2kol10_P12.abi    ----------------------------------------------------------------------------------------------------
```

```
ref/seq delPPC_hDcr CTCACCTCTTCGAGCCTCCATTGTTGGTCCACCAATGAGCTGTGTACGATTGGCTGAAAGAGTTGTAGCTCTCATTTGCTGTGAGAAACTGCACAAAATT
                     S  P  L  R  A  S  I  V  G  P  P  M  S  C  V  R  L  A  E  R  V  V  A  L  I  C  C  E  K  L  H  K  I

mK2kol10_SqrP2.ab   ----------------------------------------------------------------------------------------------------
mK2kol10_1.abi      ----------------------------------------------------------------------------------------------------
mK2kol10_P3.abi     ----------------------------------------------------------------------------------------------------
mK2kol10II_P3.abi   ----------------------------------------------------------------------------------------------------
mK2kol10_P4.abi     CTCACCTCTTCGAGCCTCCATTGTTGGTCCACCAATGAGCTGTGTACGATTGGCTGAAAGAGTTGTAGCTCTCATTTGCTGTGAGAAACTGCACAAAATT
mK2kol10II_P4.abi   CTCACCTCTTCGAGCCTCCATTGTTGGTCCACCAATGAGCTGTGTACGATTGGCTGAAAGAGTTGTAGCTCTCATTTGCTGTGAGAAACTGCACAAAATT
mK2kol10_P5.abi     ----------------------------------------------------------------------------------------------------
mK2kol10_P8.abi     ----------------------------------------------------------------------------------------------------
mK2kol10_P10.abi    ----------------------------------------------------------------------------------------------------
mK2kol10_P12.abi    ----------------------------------------------------------------------------------------------------
```

```
ref/seq delPPC_hDcr GGCGAACTGGATGACCATTTGATGCCAGTTGGGAAAGAGACTGTTAAATATGAAGAGGAGCTTGATTTGCATGATGAAGAAGAGACCAGTGTTCCAGGAA
                     G  E  L  D  D  H  L  M  P  V  G  K  E  T  V  K  Y  E  E  E  L  D  L  H  D  E  E  E  T  S  V  P  G

mK2kol10_SqrP2.ab   ---------------------------------------------------------------------------------------------------
mK2kol10_1.abi      ---------------------------------------------------------------------------------------------------
mK2kol10_P3.abi     ---------------------------------------------------------------------------------------------------
mK2kol10II_P3.abi   ---------------------------------------------------------------------------------------------------
mK2kol10_P4.abi     GGCGAACTGGATGACCATTTGATGCCAGTTGGGAAAGAGACTGTTAAATATGAAGAGGAGCTTGATTTGCATGATGAAGAAGAGACCAGTGTTCCAGGAA
mK2kol10II_P4.abi   GGCGAACTGGATGACCATTTGATGCCAGTTGGGAAAGAGACTGTTAAATATGAAGAGGAGCTTGATTTGCATGATGAAGAAGAGACCAGTGTTCCAGGAA
mK2kol10_P5.abi     ---------------------------------------------------------------------------------------------------
mK2kol10_P8.abi     ---------------------------------------------------------------------------------------------------
mK2kol10_P10.abi    ---------------------------------------------------------------------------------------------------
mK2kol10_P12.abi    ---------------------------------------------------------------------------------------------------
```

```
                                                                                     BclI*
ref/seq delPPC_hDcr GACCAGGTTCCACGAAACGAAGGCAGTGCTACCCAAAAGCAATTCCAGAGTGTTTGAGGGATAGTTATCCCAGACCTGATCAGCCCTGTTACCTGTATGT
                    R  P  G  S  T  K  R  R  Q  C  Y  P  K  A  I  P  E  C  L  R  D  S  Y  P  R  P  D  Q  P  C  Y  L  Y  V
                                                            PPC domain deletion

mK2kol10_SqrP2.ab   ---------------------------------------------------------------------------------------------------
mK2kol10_1.abi      ---------------------------------------------------------------------------------------------------
mK2kol10_P3.abi     ---------------------------------------------------------------------------------------------------
mK2kol10II_P3.abi   ---------------------------------------------------------------------------------------------------
mK2kol10_P4.abi     GACCAGGTTCCACGAAACGAAGGCAGTGCTACCCAAAAGCA------------------------------------------------------------
mK2kol10II_P4.abi   GACCAGGTTCCACGAAACGAAGGCAGTGCTACCCAAAAGCA------------------------------------------------------------
mK2kol10_P5.abi     ---------------------------------------------------------------------------------------------------
mK2kol10_P8.abi     ---------------------------------------------------------------------------------------------------
mK2kol10_P10.abi    ---------------------------------------------------------------------------------------------------
mK2kol10_P12.abi    ---------------------------------------------------------------------------------------------------
```

```
ref/seq delPPC_hDcr GATAGGAATGGTTTTAACTACACCTTTACCTGATGAACTCAACTTTAGAAGGCGGAAGCTCTATCCTCCTGAAGATACCACAAGATGCTTTGGAATACTG
                     I  G  M  V  L  T  T  P  L  P  D  E  L  N  F  R  R  R  K  L  Y  P  P  E  D  T  T  R  C  F  G  I  L

mK2kol10_SqrP2.ab   ---------------------------------------------------------------------------------------------------
mK2kol10_1.abi      ---------------------------------------------------------------------------------------------------
mK2kol10_P3.abi     ---------------------------------------------------------------------------------------------------
mK2kol10II_P3.abi   ---------------------------------------------------------------------------------------------------
mK2kol10_P4.abi     ---------------------------------------------------------------------------------------------------
mK2kol10II_P4.abi   ---------------------------------------------------------------------------------------------------
mK2kol10_P5.abi     ---------------------------------------------------------------------------------------------------
mK2kol10_P8.abi     ---------------------------------------------------------------------------------------------------
mK2kol10_P10.abi    ---------------------------------------------------------------------------------------------------
mK2kol10_P12.abi    ---------------------------------------------------------------------------------------------------
```

```
                                                                 BstEII
ref/seq delPPC_hDcr ACGGCCAAACCCATACCTCAGATTCCACACTTTCCTGTGTACACACGCTCTGGAGAGGTTACCATATCCATTGAGTTGAAGAAGTCTGGTTTCATGTTGT
                    T  A  K  P  I  P  Q  I  P  H  F  P  V  Y  T  R  S  G  E  V  T  I  S  I  E  L  K  K  S  G  F  M  L

mK2kol10_SqrP2.ab   ---------------------------------------------------------------------------------------------------
mK2kol10_1.abi      ---------------------------------------------------------------------------------------------------
mK2kol10_P3.abi     ---------------------------------------------------------------------------------------------------
mK2kol10II_P3.abi   ---------------------------------------------------------------------------------------------------
mK2kol10_P4.abi     ---------------------------------------------------------------------------------------------------
mK2kol10II_P4.abi   ---------------------------------------------------------------------------------------------------
mK2kol10_P5.abi     ---------------------------------------------------------------------------------------------------
mK2kol10_P8.abi     ---------------------------------------------------------------------------------------------------
mK2kol10_P10.abi    ---------------------------------------------------------------------------------------------------
mK2kol10_P12.abi    ---------------------------------------------------------------------------------------------------
```

DraI

```
ref/seq delPPC_hDcr CTCTACAAATGCTTGAGTTGATTACAAGACTTCACCAGTATATATTCTCACATATTCTTCGGCTTGAAAAACCTGCACTAGAATTTAAACCTACAGACGC
                    S  L  Q  M  L  E  L  I  T  R  L  H  Q  Y  I  F  S  H  I  L  R  L  E  K  P  A  L  E  F  K  P  T  D  A

mK2kol10_SqrP2.ab   ------------------------------------------------------------------------------------------------------
mK2kol10_1.abi      ------------------------------------------------------------------------------------------------------
mK2kol10_P3.abi     ------------------------------------------------------------------------------------------------------
mK2kol10II_P3.abi   ------------------------------------------------------------------------------------------------------
mK2kol10_P4.abi     ------------------------------------------------------------------------------------------------------
mK2kol10II_P4.abi   ------------------------------------------------------------------------------------------------------
mK2kol10_P5.abi     ------------------------------------------------------------------------------------------------------
mK2kol10_P8.abi     ------------------------------------------------------------------------------------------------------
mK2kol10_P10.abi    ------------------------------------------------------------------------------------------------------
mK2kol10_P12.abi    ------------------------------------------------------------------------------------------------------
```

BstXI DraI

```
ref/seq delPPC_hDcr TGATTCAGCATACTGTGTTCTACCTCTTAATGTTGTTAATGACTCCAGCACTTTGGATATTGACTTTAAATTCATGGAAGATATTGAGAAGTCTGAAGCT
                     D  S  A  Y  C  V  L  P  L  N  V  V  N  D  S  S  T  L  D  I  D  F  K  F  M  E  D  I  E  K  S  E  A

mK2kol10_SqrP2.ab   ------------------------------------------------------------------------------------------------------
mK2kol10_1.abi      ------------------------------------------------------------------------------------------------------
mK2kol10_P3.abi     ------------------------------------------------------------------------------------------------------
mK2kol10II_P3.abi   ------------------------------------------------------------------------------------------------------
mK2kol10_P4.abi     ------------------------------------------------------------------------------------------------------
mK2kol10II_P4.abi   ------------------------------------------------------------------------------------------------------
mK2kol10_P5.abi     ------------------------------------------------------------------------------------------------------
mK2kol10_P8.abi     ------------------------------------------------------------------------------------------------------
mK2kol10_P10.abi    ------------------------------------------------------------------------------------------------------
mK2kol10_P12.abi    ------------------------------------------------------------------------------------------------------
```

```
                        |BsmI                                      |DraI                                     |EcoRV
ref/seq delPPC_hDcr CGCATAGGCATTCCCAGTACAAAGTATACAAAAGAAACACCCTTTGTTTTTAAATTAGAAGATTACCAAGATGCCGTTATCATTCCAAGATATCGCAATT
                     R  I  G  I  P  S  T  K  Y  T  K  E  T  P  F  V  F  K  L  E  D  Y  Q  D  A  V  I  I  P  R  Y  R  N

mK2kol10_SqrP2.ab  -----------------------------------------------------------------------------------------------------
mK2kol10_1.abi     -----------------------------------------------------------------------------------------------------
mK2kol10_P3.abi    -----------------------------------------------------------------------------------------------------
mK2kol10II_P3.abi  -----------------------------------------------------------------------------------------------------
mK2kol10_P4.abi    -----------------------------------------------------------------------------------------------------
mK2kol10II_P4.abi  -----------------------------------------------------------------------------------------------------
mK2kol10_P5.abi    -----------------------------------------------------------------------------------------------------
mK2kol10_P8.abi    -----------------------------------------------------------------------------------------------------
mK2kol10_P10.abi   -----------------------------------------------------------------------------------------------------
mK2kol10_P12.abi   -----------------------------------------------------------------------------------------------------
```

```
                     |BclI*       |ClaI                                                                                    |SspI
ref/seq delPPC_hDcr TTGATCAGCCTCATCGATTTTATGTAGCTGATGTGTACACTGATCTTACCCCACTCAGTAAATTTCCTTCCCCTGAGTATGAAACTTTTGCAGAATATTA
                    F  D  Q  P  H  R  F  Y  V  A  D  V  Y  T  D  L  T  P  L  S  K  F  P  S  P  E  Y  E  T  F  A  E  Y  Y

mK2kol10_SqrP2.ab  -----------------------------------------------------------------------------------------------------
mK2kol10_1.abi     -----------------------------------------------------------------------------------------------------
mK2kol10_P3.abi    -----------------------------------------------------------------------------------------------------
mK2kol10II_P3.abi  -----------------------------------------------------------------------------------------------------
mK2kol10_P4.abi    -----------------------------------------------------------------------------------------------------
mK2kol10II_P4.abi  -----------------------------------------------------------------------------------------------------
mK2kol10_P5.abi    -----------------------------------------------------------------------------------------------------
mK2kol10_P8.abi    -----------------------------------------------------------------------------------------------------
mK2kol10_P10.abi   -----------------------------------------------------------------------------------------------------
mK2kol10_P12.abi   -----------------------------------------------------------------------------------------------------
```

```
ref/seq delPPC_hDcr TAAAACAAAGTACAACCTTGACCTAACCAATCTCAACCAGCCACTGCTGGATGTGGACCACACATCTTCAAGACTTAATCTTTTGACACCTCGACATTTG
                     K  T  K  Y  N  L  D  L  T  N  L  N  Q  P  L  L  D  V  D  H  T  S  S  R  L  N  L  L  T  P  R  H  L

mK2kol10_SqrP2.ab ------------------------------------------------------------------------------------------------------
mK2kol10_1.abi    ------------------------------------------------------------------------------------------------------
mK2kol10_P3.abi   ------------------------------------------------------------------------------------------------------
mK2kol10II_P3.abi ------------------------------------------------------------------------------------------------------
mK2kol10_P4.abi   ------------------------------------------------------------------------------------------------------
mK2kol10II_P4.abi ------------------------------------------------------------------------------------------------------
mK2kol10_P5.abi   ------------------------------------------------------------------------------------------------------
mK2kol10_P8.abi   ------------------------------------------------------------------------------------------------------
mK2kol10_P10.abi  ------------------------------------------------------------------------------------------------------
mK2kol10_P12.abi  ------------------------------------------------------------------------------------------------------
```

```
                                  |Eco47III                                              |PstI
ref/seq delPPC_hDcr AATCAGAAGGGGAAAGCGCTTCCTTTAAGCAGTGCTGAGAAGAGGAAAGCCAAATGGGAAAGTCTGCAGAATAAACAGATACTGGTTCCAGAACTCTGTG
                     N  Q  K  G  K  A  L  P  L  S  S  A  E  K  R  K  A  K  W  E  S  L  Q  N  K  Q  I  L  V  P  E  L  C

mK2kol10_SqrP2.ab ------------------------------------------------------------------------------------------------------
mK2kol10_1.abi    ------------------------------------------------------------------------------------------------------
mK2kol10_P3.abi   ------------------------------------------------------------------------------------------------------
mK2kol10II_P3.abi ------------------------------------------------------------------------------------------------------
mK2kol10_P4.abi   ------------------------------------------------------------------------------------------------------
mK2kol10II_P4.abi ------------------------------------------------------------------------------------------------------
mK2kol10_P5.abi   ------------------------------------------------------------------------------------------------------
mK2kol10_P8.abi   ------------------------------------------------------------------------------------------------------
mK2kol10_P10.abi  ------------------------------------------------------------------------------------------------------
mK2kol10_P12.abi  ------------------------------------------------------------------------------------------------------
```

```
                                                                                                   PstI
ref/seq delPPC_hDcr CTATACATCCAATTCCAGCATCACTGTGGAGAAAAGCTGTTTGTCTCCCCAGCATACTTTATCGCCTTCACTGCCTTTTGACTGCAGAGGAGCTAAGAGC
                     A  I  H  P  I  P  A  S  L  W  R  K  A  V  C  L  P  S  I  L  Y  R  L  H  C  L  L  T  A  E  E  L  R  A
                                                                                                          PPC do
mK2kol10_SqrP2.ab   ----------------------------------------------------------------------------------------------------
mK2kol10_1.abi      ----------------------------------------------------------------------------------------------------
mK2kol10_P3.abi     ----------------------------------------------------------------------------------------------------
mK2kol10II_P3.abi   ----------------------------------------------------------------------------------------------------
mK2kol10_P4.abi     ----------------------------------------------------------------------------------------------------
mK2kol10II_P4.abi   ----------------------------------------------------------------------------------------------------
mK2kol10_P5.abi     ------------------------------------------------------------------------------------------GAGCTAAGAGC
mK2kol10_P8.abi     ----------------------------------------------------------------------------------------------------
mK2kol10_P10.abi    ----------------------------------------------------------------------------------------------------
mK2kol10_P12.abi    ----------------------------------------------------------------------------------------------------
```

```
ref/seq delPPC_hDcr CCAGACTGCCAGCGATGCTGGCGTGGGAGTCAGATCACTTCCTGCGGATTTTAGATACCCTAACTTAGACTTCGGGTGGAAAAAATCTATTGACAGCAAA
                      Q  T  A  S  D  A  G  V  G  V  R  S  L  P  A  D  F  R  Y  P  N  L  D  F  G  W  K  K  S  I  D  S  K
                    main deletion
mK2kol10_SqrP2.ab   ----------------------------------------------------------------------------------------------------
mK2kol10_1.abi      ----------------------------------------------------------------------------------------------------
mK2kol10_P3.abi     ----------------------------------------------------------------------------------------------------
mK2kol10II_P3.abi   ----------------------------------------------------------------------------------------------------
mK2kol10_P4.abi     ----------------------------------------------------------------------------------------------------
mK2kol10II_P4.abi   ----------------------------------------------------------------------------------------------------
mK2kol10_P5.abi     CCAGACTGCCAGCGATGCTGGCGTGGGAGTCAGATCACTTCCTGCGGATTTTAGATACCCTAACTTAGACTTCGGGTGGAAAAAATCTATTGACAGCAAA
mK2kol10_P8.abi     --------------------------------------------------------------------------------------TCTATTGACAGCAAA
mK2kol10_P10.abi    ----------------------------------------------------------------------------------------------------
mK2kol10_P12.abi    ----------------------------------------------------------------------------------------------------
```

```
                   PvuII                    MunI
ref/seq delPPC_hDcr TCTTTCATCTCAATTTCTAACTCCTCTTCAGCTGAAAATGATAATTACTGTAAGCACAGCACAATTGTCCCTGAAAATGCTGCACATCAAGGTGCTAATA
                    S  F  I  S  I  S  N  S  S  S  A  E  N  D  N  Y  C  K  H  S  T  I  V  P  E  N  A  A  H  Q  G  A  N

mK2kol10_SqrP2.ab   ----------------------------------------------------------------------------------------------------
mK2kol10_1.abi      ----------------------------------------------------------------------------------------------------
mK2kol10_P3.abi     ----------------------------------------------------------------------------------------------------
mK2kol10II_P3.abi   ----------------------------------------------------------------------------------------------------
mK2kol10_P4.abi     ----------------------------------------------------------------------------------------------------
mK2kol10II_P4.abi   ----------------------------------------------------------------------------------------------------
mK2kol10_P5.abi     TCTTTCATCTCAATTTCTAACTCCTCTTCAGCTGAAAATGATAATTACTGTAAGCACAGCACAATTGTCCCTGAAAATGCTGCACATCAAGGTGCTAATA
mK2kol10_P8.abi     TCTTTCATCTCAATTTCTAACTCCTCTTCAGCTGAAAATGATAATTACTGTAAGCACAGCACAATTGTCCCTGAAAATGCTGCACATCAAGGTGCTAATA
mK2kol10_P10.abi    ----------------------------------------------------------------------------------------------------
mK2kol10_P12.abi    ----------------------------------------------------------------------------------------------------
```

```
                                                  AclI
                         XbaI                 PstI                                                BglII
ref/seq delPPC_hDcr GAACCTCCTCTCTAGAAAATCATGACCAAATGTCTGTGAACTGCAGAACGTTGCTCAGCGAGTCCCCTGGTAAGCTCCACGTTGAAGTTTCAGCAGATCT
                    R  T  S  S  L  E  N  H  D  Q  M  S  V  N  C  R  T  L  L  S  E  S  P  G  K  L  H  V  E  V  S  A  D  L

mK2kol10_SqrP2.ab   ----------------------------------------------------------------------------------------------------
mK2kol10_1.abi      ----------------------------------------------------------------------------------------------------
mK2kol10_P3.abi     ----------------------------------------------------------------------------------------------------
mK2kol10II_P3.abi   ----------------------------------------------------------------------------------------------------
mK2kol10_P4.abi     ----------------------------------------------------------------------------------------------------
mK2kol10II_P4.abi   ----------------------------------------------------------------------------------------------------
mK2kol10_P5.abi     GAACCTCCTCTCTAGAAAATCATGACCAAATGTCTGTGAACTGCAGAACGTTGCTCAGCGAGTCCCCTGGTAAGCTCCACGTTGAAGTTTCAGCAGATCT
mK2kol10_P8.abi     GAACCTCCTCTCTAGAAAATCATGACCAAATGTCTGTGAACTGCAGAACGTTGCTCAGCGAGTCCCCTGGTAAGCTCCACGTTGAAGTTTCAGCAGATCT
mK2kol10_P10.abi    ----------------------------------------------------------------------------------------------------
mK2kol10_P12.abi    ----------------------------------------------------------------------------------------------------
```

```
                     VspI
ref/seq delPPC_hDcr TACAGCAATTAATGGTCTTTCTTACAATCAAAATCTCGCCAATGGCAGTTATGATTTAGCTAACAGAGACTTTTGCCAAGGAAATCAGCTAAATTACTAC
                     T  A  I  N  G  L  S  Y  N  Q  N  L  A  N  G  S  Y  D  L  A  N  R  D  F  C  Q  G  N  Q  L  N  Y  Y

mK2kol10_SqrP2.ab  - - - - - - - - - - - - - - - - - - - - - - - - - - - - - - - - - - - - - - - - - - - - - - - - - - -
mK2kol10_1.abi     - - - - - - - - - - - - - - - - - - - - - - - - - - - - - - - - - - - - - - - - - - - - - - - - - - -
mK2kol10_P3.abi    - - - - - - - - - - - - - - - - - - - - - - - - - - - - - - - - - - - - - - - - - - - - - - - - - - -
mK2kol10II_P3.abi  - - - - - - - - - - - - - - - - - - - - - - - - - - - - - - - - - - - - - - - - - - - - - - - - - - -
mK2kol10_P4.abi    - - - - - - - - - - - - - - - - - - - - - - - - - - - - - - - - - - - - - - - - - - - - - - - - - - -
mK2kol10II_P4.abi  - - - - - - - - - - - - - - - - - - - - - - - - - - - - - - - - - - - - - - - - - - - - - - - - - - -
mK2kol10_P5.abi    TACAGCAATTAATGGTCTTTCTTACAATCAAAATCTCGCCAATGGCAGTTATGATTTAGCTAACAGAGACTTTTGCCAAGGAAATCAGCTAAATTACTAC
mK2kol10_P8.abi    TACAGCAATTAATGGTCTTTCTTACAATCAAAATCTCGCCAATGGCAGTTATGATTTAGCTAACAGAGACTTTTGCCAAGGAAATCAGCTAAATTACTAC
mK2kol10_P10.abi   - - - - - - - - - - - - - - - - - - - - - - - - - - - - - - - - - - - - - - - - - - - - - - - - - - -
mK2kol10_P12.abi   - - - - - - - - - - - - - - - - - - - - - - - - - - - - - - - - - - - - - - - - - - - - - - - - - - -
```

```
ref/seq delPPC_hDcr AAGCAGGAAATACCCGTGCAACCAACTACCTCATATTCCATTCAGAATTTATACAGTTACGAGAACCAGCCCCAGCCCAGCGATGAATGTACTCTCCTGA
                     K  Q  E  I  P  V  Q  P  T  T  S  Y  S  I  Q  N  L  Y  S  Y  E  N  Q  P  Q  P  S  D  E  C  T  L  L

mK2kol10_SqrP2.ab  - - - - - - - - - - - - - - - - - - - - - - - - - - - - - - - - - - - - - - - - - - - - - - - - - - -
mK2kol10_1.abi     - - - - - - - - - - - - - - - - - - - - - - - - - - - - - - - - - - - - - - - - - - - - - - - - - - -
mK2kol10_P3.abi    - - - - - - - - - - - - - - - - - - - - - - - - - - - - - - - - - - - - - - - - - - - - - - - - - - -
mK2kol10II_P3.abi  - - - - - - - - - - - - - - - - - - - - - - - - - - - - - - - - - - - - - - - - - - - - - - - - - - -
mK2kol10_P4.abi    - - - - - - - - - - - - - - - - - - - - - - - - - - - - - - - - - - - - - - - - - - - - - - - - - - -
mK2kol10II_P4.abi  - - - - - - - - - - - - - - - - - - - - - - - - - - - - - - - - - - - - - - - - - - - - - - - - - - -
mK2kol10_P5.abi    AAGCAGGAAATACCCGTGCAACCAACTACCTCATATTCCATTCAGAATTTATACAGTTACGAGAACCAGCCCCAGCCCAGCGATGAATGTACTCTCCTGA
mK2kol10_P8.abi    AAGCAGGAAATACCCGTGCAACCAACTACCTCATATTCCATTCAGAATTTATACAGTTACGAGAACCAGCCCCAGCCCAGCGATGAATGTACTCTCCTGA
mK2kol10_P10.abi   - - - - - - - - - - - - - - - - - - - - - - - - - - - - - - - - - - - - - - - - - - - - - - - - - - -
mK2kol10_P12.abi   - - - - - - - - - - - - - - - - - - - - - - - - - - - - - - - - - - - - - - - - - - - - - - - - - - -
```

```
ref/seq delPPC_hDcr GTAATAAATACCTTGATGGAAATGCTAACAAATCTACCTCAGATGGAAGTCCTGTGATGGCCGTAATGCCTGGTACGACAGACACTATTCAAGTGCTCAA
                    S  N  K  Y  L  D  G  N  A  N  K  S  T  S  D  G  S  P  V  M  A  V  M  P  G  T  T  D  T  I  Q  V  L  K

mK2kol10_SqrP2.ab   ---------------------------------------------------------------------------------------------------
mK2kol10_1.abi      ---------------------------------------------------------------------------------------------------
mK2kol10_P3.abi     ---------------------------------------------------------------------------------------------------
mK2kol10II_P3.abi   ---------------------------------------------------------------------------------------------------
mK2kol10_P4.abi     ---------------------------------------------------------------------------------------------------
mK2kol10II_P4.abi   ---------------------------------------------------------------------------------------------------
mK2kol10_P5.abi     GTAATAAATACCTTGATGGAAATGCTAACAAATCTACCTCAGATGGAAGTCCTGTGATGGCCGTAATGCCTGGTACGACAGACACTATTCAAGTGCTCAA
mK2kol10_P8.abi     GTAATAAATACCTTGATGGAAATGCTAACAAATCTACCTCAGATGGAAGTCCTGTGATGGCCGTAATGCCTGGTACGACAGACACTATTCAAGTGCTCAA
mK2kol10_P10.abi    ---------------------------------------------------------------------------------------------------
mK2kol10_P12.abi    ---------------------------------------------------------------------------------------------------
```

```
                                                                                                    Tth111I
ref/seq delPPC_hDcr GGGCAGGATGGATTCTGAGCAGAGCCCTTCTATTGGGTACTCCTCAAGGACTCTTGGCCCCAATCCTGGACTTATTCTTCAGGCTTTGACTCTGTCAAAC
                      G  R  M  D  S  E  Q  S  P  S  I  G  Y  S  S  R  T  L  G  P  N  P  G  L  I  L  Q  A  L  T  L  S  N

mK2kol10_SqrP2.ab   ---------------------------------------------------------------------------------------------------
mK2kol10_1.abi      ---------------------------------------------------------------------------------------------------
mK2kol10_P3.abi     ---------------------------------------------------------------------------------------------------
mK2kol10II_P3.abi   ---------------------------------------------------------------------------------------------------
mK2kol10_P4.abi     ---------------------------------------------------------------------------------------------------
mK2kol10II_P4.abi   ---------------------------------------------------------------------------------------------------
mK2kol10_P5.abi     GGGCAGGATGGATTCTGAGCAGAGCCCTTCTATTGGGTACTCCTCAAGGAC--------------------------------------------------
mK2kol10_P8.abi     GGGCAGGATGGATTCTGAGCAGAGCCCTTCTATTGGGTACTCCTCAAGGACTCTTGGCCCCAATCCTGGACTTATTCTTCAGGCTTTGACTCTGTCAAAC
mK2kol10_P10.abi    ---------------------------------------------------------------------------------------------------
mK2kol10_P12.abi    ---------------------------------------------------------------------------------------------------
```

```
                                                                            |DraI     |SphI
ref/seq delPPC_hDcr GCTAGTGATGGATTTAACCTGGAGCGGCTTGAAATGCTTGGCGACTCCTTTTTAAAGCATGCCATCACCACATATCTATTTTGCACTTACCCTGATGCGC
                     A  S  D  G  F  N  L  E  R  L  E  M  L  G  D  S  F  L  K  H  A  I  T  T  Y  L  F  C  T  Y  P  D  A

mK2kol10_SqrP2.ab   ----------------------------------------------------------------------------------------------------
mK2kol10_1.abi      ----------------------------------------------------------------------------------------------------
mK2kol10_P3.abi     ----------------------------------------------------------------------------------------------------
mK2kol10II_P3.abi   ----------------------------------------------------------------------------------------------------
mK2kol10_P4.abi     ----------------------------------------------------------------------------------------------------
mK2kol10II_P4.abi   ----------------------------------------------------------------------------------------------------
mK2kol10_P5.abi     ----------------------------------------------------------------------------------------------------
mK2kol10_P8.abi     GCTAGTGATGGATTTAACCTGGAGCGGCTTGAAATGCTTGGCGACTCCTTTTTAAAGCATGCCATCACCACATATCTATTTTGCACTTACCCTGATGCGC
mK2kol10_P10.abi    ----------------------------------------------------------------------------------------------------
mK2kol10_P12.abi    ----------------------------------------------------------------------------------------------------
```

```
                                                                                                   |BstXI
ref/seq delPPC_hDcr ATGAGGGCCGCCTTTCATATATGAGAAGCAAAAAGGTCAGCAACTGTAATCTGTATCGCCTTGGAAAAAAGAAGGGACTACCCAGCCGCATGGTGGTGTC
                    H  E  G  R  L  S  Y  M  R  S  K  K  V  S  N  C  N  L  Y  R  L  G  K  K  K  G  L  P  S  R  M  V  V  S

mK2kol10_SqrP2.ab   ----------------------------------------------------------------------------------------------------
mK2kol10_1.abi      ----------------------------------------------------------------------------------------------------
mK2kol10_P3.abi     ----------------------------------------------------------------------------------------------------
mK2kol10II_P3.abi   ----------------------------------------------------------------------------------------------------
mK2kol10_P4.abi     ----------------------------------------------------------------------------------------------------
mK2kol10II_P4.abi   ----------------------------------------------------------------------------------------------------
mK2kol10_P5.abi     ----------------------------------------------------------------------------------------------------
mK2kol10_P8.abi     ATGAGGGCCGCCTTTCATATATGAGAAGCAAAAAGGTCAGCAACTGTAATCTGTATCGCCTTGGAAAAAAGAAGGGACTACCCAGCCGCATGGTGGTGTC
mK2kol10_P10.abi    ----------------------------------------------------------------------------------------------------
mK2kol10_P12.abi    ----------------------------------------------------------------------------------------------------
```

```
                          SspI
ref/seq delPPC_hDcr       AATATTTGATCCCCCTGTGAATTGGCTTCCTCCTGGTTATGTAGTAAATCAAGACAAAAGCAACACAGATAAATGGGAAAAAGATGAAATGACAAAAGAC
                           I  F  D  P  P  V  N  W  L  P  P  G  Y  V  V  N  Q  D  K  S  N  T  D  K  W  E  K  D  E  M  T  K  D

mK2kol10_SqrP2.ab         ----------------------------------------------------------------------------------------------------
mK2kol10_1.abi            ----------------------------------------------------------------------------------------------------
mK2kol10_P3.abi           ----------------------------------------------------------------------------------------------------
mK2kol10II_P3.abi         ----------------------------------------------------------------------------------------------------
mK2kol10_P4.abi           ----------------------------------------------------------------------------------------------------
mK2kol10II_P4.abi         ----------------------------------------------------------------------------------------------------
mK2kol10_P5.abi           ----------------------------------------------------------------------------------------------------
mK2kol10_P8.abi           AATATTTGATCCCCCTGTGAATTGGCTTCCTCCTGGTTATGTAGTAAATCAAGACAAAAGCAACACAGATAAATGGGAAAAAGATGAAATGACAAAAGAC
mK2kol10_P10.abi          ----------------------------------------------------------------------------------------------------
mK2kol10_P12.abi          ----------------------------------------------------------------------------------------------------
```

```
                              SphI
ref/seq delPPC_hDcr       TGCATGCTGGCGAATGGCAAACTGGATGAGGATTACGAGGAGGAGGATGAGGAGGAGGAGAGCCTGATGTGGAGGGCTCCGAAGGAAGAGGCTGACTATG
                          C  M  L  A  N  G  K  L  D  E  D  Y  E  E  E  D  E  E  E  E  S  L  M  W  R  A  P  K  E  E  A  D  Y

mK2kol10_SqrP2.ab         ----------------------------------------------------------------------------------------------------
mK2kol10_1.abi            ----------------------------------------------------------------------------------------------------
mK2kol10_P3.abi           ----------------------------------------------------------------------------------------------------
mK2kol10II_P3.abi         ----------------------------------------------------------------------------------------------------
mK2kol10_P4.abi           ----------------------------------------------------------------------------------------------------
mK2kol10II_P4.abi         ----------------------------------------------------------------------------------------------------
mK2kol10_P5.abi           ----------------------------------------------------------------------------------------------------
mK2kol10_P8.abi           TGCATGCTGGCGAATGGCAAACTGGATGAGGATTACGAGGAGGAGGATGAGGAGGAGGAGAGCCTGATGTGGAGGGCTCCGAAGGAAGAGGCTGACTATG
mK2kol10_P10.abi          ---------------------CTGGATGAGGATTACGAGGAGGAGGATGAGGAGGAGGAGAGCCTGATGTGGAGGGCTCCGAAGGAAGAGGCTGACTATG
mK2kol10_P12.abi          ----------------------------------------------------------------------------------------------------
```

```
                     |BclI*
ref/seq delPPC_hDcr AAGATGATTTCCTGGAGTATGATCAGGAACATATCAGATTTATAGATAATATGTTAATGGGGTCAGGAGCTTTTGTAAAGAAAATCTCTCTTTCTCCTTT
                     E  D  D  F  L  E  Y  D  Q  E  H  I  R  F  I  D  N  M  L  M  G  S  G  A  F  V  K  K  I  S  L  S  P  F

mK2kol10_SqrP2.ab   ----------------------------------------------------------------------------------------------------
mK2kol10_1.abi      ----------------------------------------------------------------------------------------------------
mK2kol10_P3.abi     ----------------------------------------------------------------------------------------------------
mK2kol10II_P3.abi   ----------------------------------------------------------------------------------------------------
mK2kol10_P4.abi     ----------------------------------------------------------------------------------------------------
mK2kol10II_P4.abi   ----------------------------------------------------------------------------------------------------
mK2kol10_P5.abi     ----------------------------------------------------------------------------------------------------
mK2kol10_P8.abi     AAGATGATTTCCTGGA------------------------------------------------------------------------------------
mK2kol10_P10.abi    AAGATGATTTCCTGGAGTATGATCAGGAACATATCAGATTTATAGATAATATGTTAATGGGGTCAGGAGCTTTTGTAAAGAAAATCTCTCTTTCTCCTTT
mK2kol10_P12.abi    ----------------------------------------------------------------------------------------------------
```

```
                     |NdeI
ref/seq delPPC_hDcr TTCAACCACTGATTCTGCATATGAATGGAAAATGCCCAAAAAATCCTCCTTAGGTAGTATGCCATTTTCATCAGATTTTGAGGATTTTGACTACAGCTCT
                      S  T  T  D  S  A  Y  E  W  K  M  P  K  K  S  S  L  G  S  M  P  F  S  S  D  F  E  D  F  D  Y  S  S

mK2kol10_SqrP2.ab   ----------------------------------------------------------------------------------------------------
mK2kol10_1.abi      ----------------------------------------------------------------------------------------------------
mK2kol10_P3.abi     ----------------------------------------------------------------------------------------------------
mK2kol10II_P3.abi   ----------------------------------------------------------------------------------------------------
mK2kol10_P4.abi     ----------------------------------------------------------------------------------------------------
mK2kol10II_P4.abi   ----------------------------------------------------------------------------------------------------
mK2kol10_P5.abi     ----------------------------------------------------------------------------------------------------
mK2kol10_P8.abi     ----------------------------------------------------------------------------------------------------
mK2kol10_P10.abi    TTCAACCACTGATTCTGCATATGAATGGAAAATGCCCAAAAAATCCTCCTTAGGTAGTATGCCATTTTCATCAGATTTTGAGGATTTTGACTACAGCTCT
mK2kol10_P12.abi    ----------------------------------------------------------------------------------------------------
```

```
                       BamHI
ref/seq delPPC_hDcr TGGGATGCAATGTGCTATCTGGATCCTAGCAAAGCTGTTGAAGAAGATGACTTTGTGGTGGGGTTCTGGAATCCATCAGAAGAAAACTGTGGTGTTGACA
                     W  D  A  M  C  Y  L  D  P  S  K  A  V  E  E  D  D  F  V  V  G  F  W  N  P  S  E  E  N  C  G  V  D

mK2kol10_SqrP2.ab   ----------------------------------------------------------------------------------------------------
mK2kol10_1.abi      ----------------------------------------------------------------------------------------------------
mK2kol10_P3.abi     ----------------------------------------------------------------------------------------------------
mK2kol10II_P3.abi   ----------------------------------------------------------------------------------------------------
mK2kol10_P4.abi     ----------------------------------------------------------------------------------------------------
mK2kol10II_P4.abi   ----------------------------------------------------------------------------------------------------
mK2kol10_P5.abi     ----------------------------------------------------------------------------------------------------
mK2kol10_P8.abi     ----------------------------------------------------------------------------------------------------
mK2kol10_P10.abi    TGGGATGCAATGTGCTATCTGGATCCTAGCAAAGCTGTTGAAGAAGATGACTTTGTGGTGGGGTTCTGGAATCCATCAGAAGAAAACTGTGGTGTTGACA
mK2kol10_P12.abi    ----------------------------------------------------------------------------------------------------
```

```
ref/seq delPPC_hDcr CGGGAAAGCAGTCCATTTCTTACGACTTGCACACTGAGCAGTGTATTGCTGACAAAAGCATAGCGGACTGTGTGGAAGCCCTGCTGGGCTGCTATTTAAC
                    T  G  K  Q  S  I  S  Y  D  L  H  T  E  Q  C  I  A  D  K  S  I  A  D  C  V  E  A  L  L  G  C  Y  L  T

mK2kol10_SqrP2.ab   ----------------------------------------------------------------------------------------------------
mK2kol10_1.abi      ----------------------------------------------------------------------------------------------------
mK2kol10_P3.abi     ----------------------------------------------------------------------------------------------------
mK2kol10II_P3.abi   ----------------------------------------------------------------------------------------------------
mK2kol10_P4.abi     ----------------------------------------------------------------------------------------------------
mK2kol10II_P4.abi   ----------------------------------------------------------------------------------------------------
mK2kol10_P5.abi     ----------------------------------------------------------------------------------------------------
mK2kol10_P8.abi     ----------------------------------------------------------------------------------------------------
mK2kol10_P10.abi    CGGGAAAGCAGTCCATTTCTTACGACTTGCACACTGAGCAGTGTATTGCTGACAAAAGCATAGCGGACTGTGTGGAAGCCCTGCTGGGCTGCTATTTAAC
mK2kol10_P12.abi    ----------------------------------------------------------------------------------------------------
```

```
                    PvuII
ref/seq delPPC_hDcr CAGCTGTGGGGAGAGGGCTGCTCAGCTTTTCCTCTGTTCACTGGGGCTGAAGGTGCTCCCGGTAATTAAAAGGACTGATCGGGAAAAGGCCCTGTGCCCT
                     S  C  G  E  R  A  A  Q  L  F  L  C  S  L  G  L  K  V  L  P  V  I  K  R  T  D  R  E  K  A  L  C  P

mK2kol10_SqrP2.ab   ----------------------------------------------------------------------------------------------------
mK2kol10_1.abi      ----------------------------------------------------------------------------------------------------
mK2kol10_P3.abi     ----------------------------------------------------------------------------------------------------
mK2kol10II_P3.abi   ----------------------------------------------------------------------------------------------------
mK2kol10_P4.abi     ----------------------------------------------------------------------------------------------------
mK2kol10II_P4.abi   ----------------------------------------------------------------------------------------------------
mK2kol10_P5.abi     ----------------------------------------------------------------------------------------------------
mK2kol10_P8.abi     ----------------------------------------------------------------------------------------------------
mK2kol10_P10.abi    CAGCTGTGGGGAGAGGGCTGCTCAGCTTTTCCTCTGTTCACTGGGGCTGAAGGTGCTCCCGGTAATTAAAAGGACTGATCGGGAAAAGGCCCTGTGCCCT
mK2kol10_P12.abi    ----------------------------------------------------------------------------------------------------
```

```
                                                                          BalI
ref/seq delPPC_hDcr ACTCGGGAGAATTTCAACAGCCAACAAAAGAACCTTTCAGTGAGCTGTGCTGCTGCTTCTGTGGCCAGTTCACGCTCTTCTGTATTGAAAGACTCGGAAT
                     T  R  E  N  F  N  S  Q  Q  K  N  L  S  V  S  C  A  A  A  S  V  A  S  S  R  S  S  V  L  K  D  S  E

mK2kol10_SqrP2.ab   ----------------------------------------------------------------------------------------------------
mK2kol10_1.abi      ----------------------------------------------------------------------------------------------------
mK2kol10_P3.abi     ----------------------------------------------------------------------------------------------------
mK2kol10II_P3.abi   ----------------------------------------------------------------------------------------------------
mK2kol10_P4.abi     ----------------------------------------------------------------------------------------------------
mK2kol10II_P4.abi   ----------------------------------------------------------------------------------------------------
mK2kol10_P5.abi     ----------------------------------------------------------------------------------------------------
mK2kol10_P8.abi     ----------------------------------------------------------------------------------------------------
mK2kol10_P10.abi    ACTCGGGAGAATTTCAACAGCCAACAAAAGAACCTTTCAGTGAGCTGTGCTGCTGCTTCTGTGGCCAGTTCACGCTCTTCTGTATTGAAAGACTCGGAAT
mK2kol10_P12.abi    ----------------------------------------------------------------------------------------------------
```

```
                                                   BclI*
ref/seq delPPC_hDcr ATGGTTGTTTGAAGATTCCACCAAGATGTATGTTTGATCATCCAGATGCAGATAAAACACTGAATCACCTTATATCGGGGTTTGAAAATTTTGAAAAGAA
                    Y  G  C  L  K  I  P  P  R  C  M  F  D  H  P  D  A  D  K  T  L  N  H  L  I  S  G  F  E  N  F  E  K  K

mK2kol10_SqrP2.ab   -----------------------------------------------------------------------------------------------------
mK2kol10_1.abi      -----------------------------------------------------------------------------------------------------
mK2kol10_P3.abi     -----------------------------------------------------------------------------------------------------
mK2kol10II_P3.abi   -----------------------------------------------------------------------------------------------------
mK2kol10_P4.abi     -----------------------------------------------------------------------------------------------------
mK2kol10II_P4.abi   -----------------------------------------------------------------------------------------------------
mK2kol10_P5.abi     -----------------------------------------------------------------------------------------------------
mK2kol10_P8.abi     -----------------------------------------------------------------------------------------------------
mK2kol10_P10.abi    ATGGTTGTTTGAAGATTCCACCAAGATGTATGTTTGATCATCCAGATGCAGATAAAACACTGAATCACCTTATATCGGGGTTTGAAAATTTTGAAAAGAA
mK2kol10_P12.abi    -----------------------------------------------------------------------------------------------------
```

```
                                                                                                  Eco47III Ec
ref/seq delPPC_hDcr AATCAACTACAGATTCAAGAATAAGGCTTACCTTCTCCAGGCTTTTACACATGCCTCCTACCACTACAATACTATCACTGATTGTTACCAGCGCTTAGAA
                      I  N  Y  R  F  K  N  K  A  Y  L  L  Q  A  F  T  H  A  S  Y  H  Y  N  T  I  T  D  C  Y  Q  R  L  E

mK2kol10_SqrP2.ab   -----------------------------------------------------------------------------------------------------
mK2kol10_1.abi      -----------------------------------------------------------------------------------------------------
mK2kol10_P3.abi     -----------------------------------------------------------------------------------------------------
mK2kol10II_P3.abi   -----------------------------------------------------------------------------------------------------
mK2kol10_P4.abi     -----------------------------------------------------------------------------------------------------
mK2kol10II_P4.abi   -----------------------------------------------------------------------------------------------------
mK2kol10_P5.abi     -----------------------------------------------------------------------------------------------------
mK2kol10_P8.abi     -----------------------------------------------------------------------------------------------------
mK2kol10_P10.abi    AATCAACTACAGATTCAAGAATAAGGCTTACCTTCTCCAGGCTTTTACACATGCCTCCTACCACTACAATACTATCACTGATTGTTACCAGCGCTTAGAA
mK2kol10_P12.abi    -----------------------------------------------------------------------------------------------------
```

```
                     oRI                                                      SacII          XmaI SmaI
ref/seq delPPC_hDcr  TTCCTGGGAGATGCGATTTTGGACTACCTCATAACCAAGCACCTTTATGAAGACCCGCGGCAGCACTCCCCGGGGGTCCTGACAGACCTGCGGTCTGCCC
                      F  L  G  D  A  I  L  D  Y  L  I  T  K  H  L  Y  E  D  P  R  Q  H  S  P  G  V  L  T  D  L  R  S  A

mK2kol10_SqrP2.ab    ------------------------------------------------------------------------------------------------------
mK2kol10_1.abi       ------------------------------------------------------------------------------------------------------
mK2kol10_P3.abi      ------------------------------------------------------------------------------------------------------
mK2kol10II_P3.abi    ------------------------------------------------------------------------------------------------------
mK2kol10_P4.abi      ------------------------------------------------------------------------------------------------------
mK2kol10II_P4.abi    ------------------------------------------------------------------------------------------------------
mK2kol10_P5.abi      ------------------------------------------------------------------------------------------------------
mK2kol10_P8.abi      ------------------------------------------------------------------------------------------------------
mK2kol10_P10.abi     TTCCTGGGAGATGCGATTTTGGACTACCTCATAACCAAGCACCTTTATGAAGACCCGCGGCAGCACTCCCCGGGGGTCCTGACAGACCTGCGGTCTGCCC
mK2kol10_P12.abi     ---------------------------------------------------GACCCGCGGCAGCACTCCCCGGGGGTCCTGACAGACCTGCGGTCTGCCC
```

```
                                                                                ScaI                 Bpu10I EcoICRI SacI
ref/seq delPPC_hDcr  TGGTCAACAACACCATCTTTGCATCGCTGGCTGTAAAGTACGACTACCACAAGTACTTCAAAGCTGTCTCTCCTGAGCTCTTCCATGTCATTGATGACTT
                     L  V  N  N  T  I  F  A  S  L  A  V  K  Y  D  Y  H  K  Y  F  K  A  V  S  P  E  L  F  H  V  I  D  D  F

mK2kol10_SqrP2.ab    ------------------------------------------------------------------------------------------------------
mK2kol10_1.abi       ------------------------------------------------------------------------------------------------------
mK2kol10_P3.abi      ------------------------------------------------------------------------------------------------------
mK2kol10II_P3.abi    ------------------------------------------------------------------------------------------------------
mK2kol10_P4.abi      ------------------------------------------------------------------------------------------------------
mK2kol10II_P4.abi    ------------------------------------------------------------------------------------------------------
mK2kol10_P5.abi      ------------------------------------------------------------------------------------------------------
mK2kol10_P8.abi      ------------------------------------------------------------------------------------------------------
mK2kol10_P10.abi     TGGTCAACAACACCATCTTTGCATCGCTGGCTGTAAAGTACGACTACCACAAGTACTTCAAAGCTGTC-----------------------------------
mK2kol10_P12.abi     TGGTCAACAACACCATCTTTGCATCGCTGGCTGTAAAGTACGACTACCACAAGTACTTCAAAGCTGTCTCTCCTGAGCTCTTCCATGTCATTGATGACTT
```

```
                                                          Bpu10I  BglII
ref/seq delPPC_hDcr TGTGCAGTTTCAGCTTGAGAAGAATGAAATGCAAGGAATGGATTCTGAGCTTAGGAGATCTGAGGAGGATGAAGAGAAAGAAGAGGATATTGAAGTTCCA
                      V  Q  F  Q  L  E  K  N  E  M  Q  G  M  D  S  E  L  R  R  S  E  E  D  E  E  K  E  E  D  I  E  V  P

mK2kol10_SqrP2.ab   ----------------------------------------------------------------------------------------------------
mK2kol10_1.abi      ----------------------------------------------------------------------------------------------------
mK2kol10_P3.abi     ----------------------------------------------------------------------------------------------------
mK2kol10II_P3.abi   ----------------------------------------------------------------------------------------------------
mK2kol10_P4.abi     ----------------------------------------------------------------------------------------------------
mK2kol10II_P4.abi   ----------------------------------------------------------------------------------------------------
mK2kol10_P5.abi     ----------------------------------------------------------------------------------------------------
mK2kol10_P8.abi     ----------------------------------------------------------------------------------------------------
mK2kol10_P10.abi    ----------------------------------------------------------------------------------------------------
mK2kol10_P12.abi    TGTGCAGTTTCAGCTTGAGAAGAATGAAATGCAAGGAATGGATTCTGAGCTTAGGAGATCTGAGGAGGATGAAGAGAAAGAAGAGGATATTGAAGTTCCA
```

```
                        NcoI                                      BstXI
ref/seq delPPC_hDcr AAGGCCATGGGGGATATTTTTGAGTCGCTTGCTGGTGCCATTTACATGGATAGTGGGATGTCACTGGAGACAGTCTGGCAGGTGTACTATCCCATGATGC
                     K  A  M  G  D  I  F  E  S  L  A  G  A  I  Y  M  D  S  G  M  S  L  E  T  V  W  Q  V  Y  Y  P  M  M

mK2kol10_SqrP2.ab   ----------------------------------------------------------------------------------------------------
mK2kol10_1.abi      ----------------------------------------------------------------------------------------------------
mK2kol10_P3.abi     ----------------------------------------------------------------------------------------------------
mK2kol10II_P3.abi   ----------------------------------------------------------------------------------------------------
mK2kol10_P4.abi     ----------------------------------------------------------------------------------------------------
mK2kol10II_P4.abi   ----------------------------------------------------------------------------------------------------
mK2kol10_P5.abi     ----------------------------------------------------------------------------------------------------
mK2kol10_P8.abi     ----------------------------------------------------------------------------------------------------
mK2kol10_P10.abi    ----------------------------------------------------------------------------------------------------
mK2kol10_P12.abi    AAGGCCATGGGGGATATTTTTGAGTCGCTTGCTGGTGCCATTTACATGGATAGTGGGATGTCACTGGAGACAGTCTGGCAGGTGTACTATCCCATGATGC
```

```
ref/seq delPPC_hDcr GGCCACTAATAGAAAAGTTTTCTGCAAATGTACCCCGTTCCCCTGTGCGAGAATTGCTTGAAATGGAACCAGAAACTGCCAAATTTAGCCCGGCTGAGAG
                    R  P  L  I  E  K  F  S  A  N  V  P  R  S  P  V  R  E  L  L  E  M  E  P  E  T  A  K  F  S  P  A  E  R

mK2kol10_SqrP2.ab   ----------------------------------------------------------------------------------------------------
mK2kol10_1.abi      ----------------------------------------------------------------------------------------------------
mK2kol10_P3.abi     ----------------------------------------------------------------------------------------------------
mK2kol10II_P3.abi   ----------------------------------------------------------------------------------------------------
mK2kol10_P4.abi     ----------------------------------------------------------------------------------------------------
mK2kol10II_P4.abi   ----------------------------------------------------------------------------------------------------
mK2kol10_P5.abi     ----------------------------------------------------------------------------------------------------
mK2kol10_P8.abi     ----------------------------------------------------------------------------------------------------
mK2kol10_P10.abi    ----------------------------------------------------------------------------------------------------
mK2kol10_P12.abi    GGCCACTAATAGAAAAGTTTTCTGCAAATGTACCCCGTTCCCCTGTGCGAGAATTGCTTGAAATGGAACCAGAAACTGCCAAATTTAGCCCGGCTGAGAG
```

```
                                                                DraI                                         PstI
ref/seq delPPC_hDcr AACTTACGACGGGAAGGTCAGAGTCACTGTGGAAGTAGTAGGAAAGGGGAAATTTAAAGGTGTTGGTCGAAGTTACAGGATTGCCAAATCTGCAGCAGCA
                      T  Y  D  G  K  V  R  V  T  V  E  V  V  G  K  G  K  F  K  G  V  G  R  S  Y  R  I  A  K  S  A  A  A

mK2kol10_SqrP2.ab   ----------------------------------------------------------------------------------------------------
mK2kol10_1.abi      ----------------------------------------------------------------------------------------------------
mK2kol10_P3.abi     ----------------------------------------------------------------------------------------------------
mK2kol10II_P3.abi   ----------------------------------------------------------------------------------------------------
mK2kol10_P4.abi     ----------------------------------------------------------------------------------------------------
mK2kol10II_P4.abi   ----------------------------------------------------------------------------------------------------
mK2kol10_P5.abi     ----------------------------------------------------------------------------------------------------
mK2kol10_P8.abi     ----------------------------------------------------------------------------------------------------
mK2kol10_P10.abi    ----------------------------------------------------------------------------------------------------
mK2kol10_P12.abi    AACTTACGACGGGAAGGTCAGAGTCACTGTGGAAGTAGTAGGAAAGGGGAAATTTAAAGGTGTTGGTCGAAGTTACAGGATTGCCAAATCTGCAGCAGCA
```

```
ref/seq delPPC_hDcr AGAAGAGCCCTCCGAAGCCTCAAAGCTAATCAACCTCAGGTTCCCAATAGCGGTGGCGGAGGTTCTGGAGGCGGTGGAAGTGACTACAAGGACGACGATG
                    R  R  A  L  R  S  L  K  A  N  Q  P  Q  V  P  N  S  G  G  G  G  S  G  G  G  G  S  D  Y  K  D  D  D

mK2kol10_SqrP2.ab   ----------------------------------------------------------------------------------------------------
mK2kol10_1.abi      ----------------------------------------------------------------------------------------------------
mK2kol10_P3.abi     ----------------------------------------------------------------------------------------------------
mK2kol10II_P3.abi   ----------------------------------------------------------------------------------------------------
mK2kol10_P4.abi     ----------------------------------------------------------------------------------------------------
mK2kol10II_P4.abi   ----------------------------------------------------------------------------------------------------
mK2kol10_P5.abi     ----------------------------------------------------------------------------------------------------
mK2kol10_P8.abi     ----------------------------------------------------------------------------------------------------
mK2kol10_P10.abi    ----------------------------------------------------------------------------------------------------
mK2kol10_P12.abi    AGAAGAGCCCTCCGAAGCCTCAAAGCTAATCAACCTCAGGTTCCCAATAGCGGTGGCGGAGGTTCTGGAGGCGGTGGAAGTGACTACAAGGACGACGATG
```

```
                                                                                              XhoI
ref/seq delPPC_hDcr ACAAGGATTACAAAGACGATGATGACAAGGACTATAAGGACGATGACGATAAGTAACATTTGGTTTAGTGTACAATATCTCCTCGAGCATTTGGTTTAGT
                    D  K  D  Y  K  D  D  D  D  K  D  Y  K  D  D  D  D  K  *  H  L  V  *  C  T  I  S  P  R  A  F  G  L  V
                                                                          Stop

mK2kol10_SqrP2.ab   ----------------------------------------------------------------------------------------------------
mK2kol10_1.abi      ----------------------------------------------------------------------------------------------------
mK2kol10_P3.abi     ----------------------------------------------------------------------------------------------------
mK2kol10II_P3.abi   ----------------------------------------------------------------------------------------------------
mK2kol10_P4.abi     ----------------------------------------------------------------------------------------------------
mK2kol10II_P4.abi   ----------------------------------------------------------------------------------------------------
mK2kol10_P5.abi     ----------------------------------------------------------------------------------------------------
mK2kol10_P8.abi     ----------------------------------------------------------------------------------------------------
mK2kol10_P10.abi    ----------------------------------------------------------------------------------------------------
mK2kol10_P12.abi    ACAAGGATTACAAAGACGATGATGACAAGGACTATAAGGACGATGACGATAAGTAACATTTGGTTTAGTGTACAATATCTCCTCGA--------------
```

XhoI

```
ref/seq delPPC_hDcr GTACAATATCTCCTCGAGGGCTCGTGGCCTCGACTGTGCCTTCTAGTTGCCAGCCATCTGTTGTTTGC
                      Y  N  I  S  S  R  A  R  G  L  D  C  A  F  *  L  P  A  I  C  C  L

mK2kol10_SqrP2.ab   ---------------------------------------------------------------------
mK2kol10_1.abi      ---------------------------------------------------------------------
mK2kol10_P3.abi     ---------------------------------------------------------------------
mK2kol10II_P3.abi   ---------------------------------------------------------------------
mK2kol10_P4.abi     ---------------------------------------------------------------------
mK2kol10II_P4.abi   ---------------------------------------------------------------------
mK2kol10_P5.abi     ---------------------------------------------------------------------
mK2kol10_P8.abi     ---------------------------------------------------------------------
mK2kol10_P10.abi    ---------------------------------------------------------------------
mK2kol10_P12.abi    ---------------------------------------------------------------------
```

```
ref/seq delPPC_hDcr

mK2kol10_SqrP2.ab
mK2kol10_1.abi
mK2kol10_P3.abi
mK2kol10II_P3.abi
mK2kol10_P4.abi
mK2kol10II_P4.abi
mK2kol10_P5.abi
mK2kol10_P8.abi
mK2kol10_P10.abi
mK2kol10_P12.abi
```
